# Frailty prevalence in 42 European countries by age and gender: development of the SHARE Frailty Atlas for Europe

**DOI:** 10.1007/s11357-023-00975-3

**Published:** 2023-10-19

**Authors:** János G. Pitter, Antal Zemplényi, Balázs Babarczy, Bertalan Németh, Zoltán Kaló, Zoltán Vokó

**Affiliations:** 1 Syreon Research Institute, Budapest, Hungary; 2 Faculty of Pharmacy, Center for Health Technology Assessment and Pharmacoeconomic Research, University of Pécs, Pécs, Hungary; 3 Center for Health Technology Assessment, Semmelweis University, Budapest, Hungary

**Keywords:** Frailty prevalence, Europe, Gross domestic product, Purchasing power parity, Survey of Health, Ageing and Retirement in Europe (SHARE), SHARE Frailty Atlas for Europe

## Abstract

Comparative frailty prevalence data across European countries is sparse due to heterogeneous measurement methods. The Survey of Health, Ageing and Retirement (SHARE) initiative conducted interviews with probability sampling of non-institutionalized elderly people in several European countries. Previous frailty analyses of SHARE datasets were limited to initial SHARE countries and did not provide age- and gender-stratified frailty prevalence. Our aim was to provide age- and gender-stratified frailty prevalence estimates in all European countries, with predictions where necessary. From 29 SHARE participating countries, 311,915 individual surveys were analyzed. Frailty prevalence was estimated by country and gender in 5-year age bands using the SHARE Frailty Instrument and a frailty index. Association of frailty prevalence with age, gender, and GDP per capita (country-specific economic indicator for predictions) was investigated in multivariate mixed logistic regression models with or without multiple imputation. Female gender and increasing age were significantly associated with higher frailty prevalence. Higher GDP per capita, with or without purchasing power parity adjustment, was significantly associated with lower frailty prevalence in the 65–79 age groups in all analyses. Observed and predicted data on frailty rates by country are provided in the interactive SHARE Frailty Atlas for Europe. Our study provides age- and gender-stratified frailty prevalence estimates for all European countries, revealing remarkable between-country heterogeneity. Higher frailty prevalence is strongly associated with lower GDP per capita, underlining the importance of investigating transferability of evidence across countries at different developmental levels and calling for improved policies to reduce inequity in risk of developing frailty across European countries.

**Supplementary Information:**

The online version contains supplementary material available at 10.1007/s11357-023-00975-3.

## Introduction

### Sparse comparative data on frailty prevalence in European countries

Frailty is an age-related condition developing on the grounds of gradual physiological deteriorations in multiple organ systems, undermining biological resilience and hence, predisposing for large health decline upon minor illnesses or stressors [1–3]. Classification rules for frailty include the Fried phenotype paradigm [4] (where an older individual is frail if he/she meets at least three of the following five criteria: unintentional weight loss, weakness, poor self-reported endurance, slow walking speed, and low physical activity), calculation of accumulated deficits using the frailty index approach [5, 6] , tailored clinical scales including the Clinical Frailty Scale [7] , or assessment methods based on healthcare payer records like the Hospital Frailty Risk Score [8] . There are important conceptual nuances behind these classification approaches, with an ongoing debate on whether frailty is a pre-disability phase as suggested by the frailty phenotype definition, or comprises severe deficits and disability as supposed in the frailty index approach. Inclusion of psychological and social deficit criteria in more holistic frailty definitions comes with additional complexity and heterogeneity across studies [9] . Hence, albeit the various operative definitions of frailty may have comparable ability to predict all-cause mortality [10] , these are not interchangeable but should rather be considered complementary [11, 12] .

Importantly, the risk of becoming frail is increasing with age in the elderly independently of the assessment instrument and is more common in women [13, 14] . Screening for frailty in clinical routine confers benefits with regards to identification of target subjects for personalized integrated care interventions [14] and to the best allocation of scarce healthcare resources [15] , although the ethical considerations on frailty-based triage need further research and clarification [16–19]. Frailty research is in the forefront of WHO efforts to better understand ‘intrinsic capacity’ of patients so that it can be utilized in clinical settings, including prevention and health-promotion programs [20]. Besides the patient-level potential benefits of frailty assessment, epidemiology of frailty is also of public health interest. Prevalence rate of frailty in age-standardized populations or within specific age bands is an overall indicator of population health status, integrating various dimensions of deficits and disabilities into a single aggregated metric which is an independent predictor of hospitalization and overall survival [2, 3, 21, 22]. Multivariate analyses in selected community-dwelling elderly populations allowed the identification of risk factors for developing frailty, including low education and socioeconomic status [13, 23–25], nutritional factors [26–28], smoking [9, 29], or physical inactivity [9, 30, 31]. However, most of the published studies focused on single-country analyses, and the differences between study designs, especially in frailty measurement methodology, limit the comparability of frailty epidemiology across countries [13]. The Survey of Health, Ageing and Retirement (SHARE) initiative is a unique development in this respect, conducting 530,000 structured interviews with anthropometric measurements and performance tests (e.g., body mass index and grip strength tests) with 140,000 non-institutionalized people aged 50 or older from 28 European countries and Israel, in eight consecutive waves from 2004 to 2020 [32, 33].

### Prior between-country comparisons of frailty epidemiology using the SHARE database

Many alternative frailty assessment approaches have been implemented and validated using the SHARE database. The Fried phenotype criteria were mapped to SHARE survey items by Santos-Eggimann et al., and frailty prevalence in the 65 years and older community-dwelling population was compared across ten EU-15 countries. A geographical gradient was observed from North to South with highest frailty rates in Italy and Spain. Controlling for age, gender and educational years in multivariate regression analyses diminished this geographical difference [34]. Romero-Ortuno et al. further developed this frailty phenotype definition into the SHARE Frailty Instrument where the mapped SHARE survey items were weighted based on discrete factor model findings in latent class analysis, proposing separate weights and frailty thresholds for males and females [35]. The SHARE Frailty Instrument score correlates with individual sociodemographic, physical, functional, psychological, and cognitive characteristics [35], and is a significant predictor of incident disability [36] and overall survival [35, 37, 38]. Notably, the SHARE Frailty Instrument was shown to have similar performance in mortality prediction to a frailty index that requires more complex data collection via comprehensive geriatric assessment [38]. Unfortunately, no between-country frailty prevalence comparisons using the Frailty Instrument could be retrieved from the scientific literature.

Various frailty index approaches have also been proposed using the SHARE datasets. The frailty index approach is intentionally flexible regarding the number and the selection of the covered deficits, as far as the general principles of item selection are met [5, 6]. In theory, the more variables included in a frailty index, the more precise frailty estimates can be derived with the higher predictive value for mortality [39]. However, a frailty index with 30–40 variables has been shown to be sufficiently accurate and the estimates become unstable only when the number of deficits was small—about 15 or less [6, 39]. Based on the datasets collected in the first and second SHARE waves in 15 EU countries, Theou et al. proposed a 70-item frailty index and showed that it was associated with increased risk of mortality after adjusting for age and sex [40]. In this study, a significant negative correlation was described between the mean frailty index and gross domestic product (GDP) per capita at purchasing power parity (PPP) in between-country comparisons of 15 EU countries. Although this analysis was clearly adequate to illustrate that the comparison of frailty prevalence rates between European countries is feasible and may reveal important differences, the statistical approach applied did not fully exploit the high number of unique observations, as only the country averages were compared. Romero-Ortuno et al. proposed another, 40-item SHARE frailty index based on SHARE Wave 1 datasets that was a stronger predictor of mortality than chronological age [41] and showed that its ability to predict mortality was similar to that of the SHARE Frailty Instrument [38]. Unfortunately, no between-country analyses could be revealed in the literature using this frailty index tool. Another SHARE frailty index encompassing 39 SHARE deficit items was proposed by Harttgen et al., which was designed to harmonize with a similar frailty index construct used on datasets from the Study on Global Ageing and Adult Health (SAGE) [42]. Using these indices, frailty index distributions of 14 European SHARE countries and 6 lower income SAGE countries (China, Mexico, Ghana, South Africa, India, and Russia) were compared after weighting the survey respondents to match the WHO world standard population distribution in each country. In this comparison, the mean frailty index tended to be lower in lower income SAGE countries than in the analyzed SHARE countries. The authors raised that this trend could reflect survival bias, i.e., longer survival of frail people in higher income countries where more developed social support and health services were available [42]. Recently, a 63-item SHARE frailty index was also described and analyzed using data from the first, second, and third waves of SHARE in 4 European countries [25].

The SHARE data collection efforts have already accomplished eight survey waves, gradually involving more and more European countries. However, following the initial flourishing of between-country frailty comparisons based the first waves of SHARE, subsequent waves of SHARE data collection remained almost untouched in this respect. The association between the economic wealth of a country and the prevalence of frailty has already been demonstrated [40]. Since SHARE contains frailty data for many countries and GDP per capita is available for all countries, it is worth examining whether GDP per capita is a good predictor of frailty prevalence. If this is the case, that would allow estimating frailty prevalence in countries without observed data.

### Study aims

The primary aim of our analysis was to provide stratified estimates of frailty prevalence rates by narrow, 5-year age bands and gender in each SHARE participating country, based on all available survey waves of SHARE and adopting two alternative frailty assessment methods: the SHARE Frailty Instrument [30], and a well-documented SHARE frailty index with reproducible methodology [38, 41]. Furthermore, an additional study aim was to characterize the association of national GDP/capita with frailty in a multivariate mixed general linear regression model and to provide predictions for age- and gender-stratified frailty prevalence rates in European countries not covered by the SHARE data collection, including Western Balkan countries, Moldova, and Ukraine. Finally, development of an interactive SHARE Frailty Atlas for Europe with user-friendly presentation of observed and model-predicted frailty prevalence rates by country was also an important research aim of our team to facilitate the exploitation of these results by researchers and other health policy stakeholders. An example for possible exploitation of these findings in the IMI2 VITAL project is described in the “Discussion” section.

## Methods

### Data source

Our research comprised of a secondary analysis of previously collected and anonymized data from Waves 1 to 8 of the Survey of Health, Ageing and Retirement in Europe (SHARE) [43–50]. The methodological details on data collection have been published elsewhere [32, 33]. SHARE is a research infrastructure for studying the effects of health, social, economic, and environmental policies over the life-course of European citizens and beyond. From 2004 until 2022, 530,000 in-depth interviews with 140,000 people aged 50 or older from 28 European countries and Israel have been conducted. Thus, SHARE is the largest pan-European social science panel study providing internationally comparable longitudinal micro data which allow insights in the fields of public health and socio-economic living conditions of European individuals. Due to probability sampling in participating countries, SHARE participants represent the non-institutionalized country populations aged 50 and older.

### Study population

**Fig. 1:**
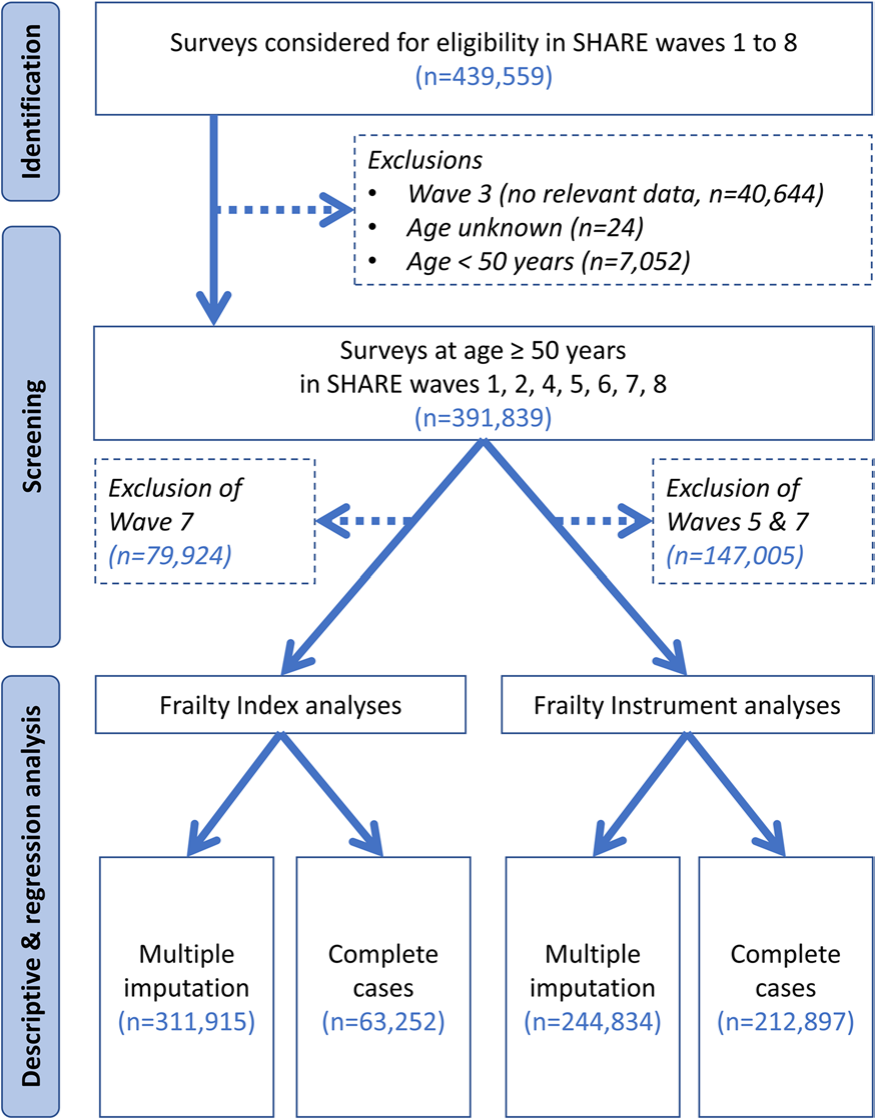
Study population flowchart

All surveys with participants aged 50 years or older at the time of their survey in SHARE waves 1, 2, 4, 5, 6, and 8 were selected for inclusion. Waves 3 and 7 were excluded from our analyses due to almost complete missingness of multiple key study variables. Wave 5 was also excluded from the Frailty Instrument analyses, due to > 80% missingness of grip strength data which is a core component of the Frailty Instrument. On the contrary, Wave 5 data were not excluded from frailty index analyses where grip strength was only one of the 40 index components. Within the included SHARE waves, surveys with incomplete data on frailty-related variables were included in the main analysis using multiple imputation and were excluded from a complete-case sensitivity analysis via listwise deletion. For a study population flowchart, please see Fig. 1.

### Frailty assessment

The SHARE Frailty Instrument was applied as described previously by Romero et al. [35]. In brief, first a discrete factor score was calculated based on grip strength measurements and self-reported survey items on fatigue; loss of appetite and/or eating less than usual; difficulties climbing stairs and/or walking 100 m; and low level of physical activity. To calculate the discrete factor score, the published equations and weights were applied [35]. Study subjects were classified as frail or non-frail based on their scores and the published gender-specific frailty thresholds [35]. The 40-item SHARE frailty index was applied as previously described by [41], with minor modifications. This tool covers difficulties in activities of daily living (16 items), prevalence of chronic diseases (10 items), and other deficits in physical and mental health (Online Resource Table S1). Notably, some of the originally described deficit variables required adjustments to reflect changes in survey items and field codes across SHARE waves. Some survey variables were renamed from Wave 5 without content change, while another original survey item (“Doctor told you had: arthritis”) have been dichotomized from Wave 5 (“Doctor told you had: rheumatoid arthritis” and “Doctor told you had: osteoarthritis/ other rheumatism”). For this deficit, a positive answer to any of the dichotomized items were considered a positive answer to the original survey item. Another adaptation was necessary for the European version of self-perceived health variable which was captured only in the first wave of SHARE. Accordingly, the US version of self-perceived health which was collected in all included SHARE waves was used in our analyses. The US version was scored similarly to the European version (best category = 0, worst category = 1, and 0.25, 0.5, and 0.75 for the intermediate ordinal options). Finally, two frailty index components have been deprecated from Wave 5 without replacement: “Doctor told you had: osteoporosis” and “For the past six months at least, have you been bothered by: breathlessness, difficulty breathing?”. Accordingly, SHARE waves 5, 6, and 8 were excluded from the complete case frailty index analyses and were included in the full sample analyses via multiple imputation of missing variables as described in the statistical methods section. After these adjustments, all deficit variables were scored in the 0 to 1 range as originally described [41], and the deficit scores were averaged to produce a frailty index between 0 (no deficit) to 1 (all deficits are fully present). Based on this frailty index, subjects were classified as frail (frailty index ≥ 0.25) or non-frail, adopting the previously established frailty threshold for the SHARE frailty index [25, 40, 41]. To ensure the reproducibility of the applied methods, annotated data management and statistical analysis scripts are available as online supplementary materials (see the Online Resource).

### Other study variables

The age of subjects at the time of the survey was determined based on either the reported subject age at interview, or age in the year of survey, or the difference of survey date and birth date. Surveys with unknown patient age were not analyzed. To allow for non-linear association of age with frailty rates, age of subjects at the time of the survey was analyzed as a categorical variable by 5-year age bands (Table 1).

**Table 1:** Study population characteristics

	Frailty instrument analyses		Frailty index analyses	
	Full sample	Complete cases	Full sample	Complete cases
Number of countries*	29	29	29	15
Number of subjects	122,458	112,749	128,328	46,342
Number of surveys	244,834	212,897	311,915	63,252
Gender: male	44.46%	44.69%	44.55%	46.42%
Age: median (quartiles) years	67 (59–75)	66 (59–73)	66 (59–75)	63 (56–71)
Age 50–54 years	10.62%	11.47%	10.76%	18.13%
Age 55–59 years	15.50%	16.57%	15.62%	19.69%
Age 60–64 years	17.14%	18.21%	17.22%	17.99%
Age 65–69 years	16.47%	17.24%	16.51%	15.14%
Age 70–74 years	14.17%	14.46%	14.07%	12.47%
Age 75–79 years	11.34%	10.95%	11.24%	8.80%
Age 80–84 years	8.10%	7.01%	8.02%	5.29%
Age 85 + years	6.66%	4.09%	6.55%	2.49%
Education years: median (quartiles)	11 (8–14)	12 (8–14)	11 (8–14)	12 (8–14)
Marital status: living alone	14.07%	14.18%	11.04%	20.21%
Marital status: with spouse/partner	30.84%	32.53%	24.21%	52.90%
Marital status: unknown	55.09%	53.29%	64.75%	26.89%
Self-perceived health: good	54.20%	59.13%	42.55%	54.02%
Self-perceived health: not good	35.71%	34.58%	28.03%	25.33%
Self-perceived health: unknown	10.09%	6.29%	29.42%	20.65%

*No data collection in Albania, Belarus, Bosnia and Herzegovina, Iceland, Kosovo, Moldova, Montenegro, North Macedonia, Norway, Russia, Serbia, Ukraine, and the United Kingdom

Auxiliary variables for multiple imputations included education years, self-perceived health status (good or not good) and living situation (with or without a spouse/partner). Missing information on education years was overwritten by available information on the same subject from other survey waves, and education years were categorized as quartiles. For multiple imputation in the frailty index analyses, drugs taken for osteoporosis was an additional auxiliary variable.

Data on annual Gross Domestic Product (GDP) per capita was collected from Eurostat and averaged in the last five years preceding the first wave of SHARE (years 2000–2004) to reduce noise due to between-year fluctuations in GDP per capita, assuming that the observed modest time-dependent changes within the study period were not relevant from the perspective of the long-term process of developing frailty. For countries without Eurostat data (e.g., Belarus, Israel, Moldova, Russian Federation, Ukraine), annual GDP per capita data was captured from World Bank and converted to Euro using European Central Bank annual mean exchange rates in the corresponding years. Gross domestic product per capita was also analyzed after adjusting for purchasing power parity (PPP). For detailed data sources and methods of purchasing power parity adjustment, please see the Online Resource.

### Handling of missing data

Exclusion of surveys with incomplete data for frailty assessment results in biased estimation of frailty prevalence if missingness is not at random. For multiple imputation, the mice R package (version 3.16.0) was applied [51], deriving ten imputed datasets based on predictive mean matching and logistic regression for numeric and binary variables, respectively. For the Frailty Instrument analyses, the imputations considered age band, gender, the five Frailty Instrument components, education years, self-perceived health, and living with or without spouse/partner as predictive factors. The frailty index dataset imputations considered age band, gender, the 40 index components, exposure to osteoporosis drugs, education years, self-perceived health, and living with or without spouse/partner as predictive factors. Incomplete imputation of frailty index components occurred in several cases with combined missingness of multiple frailty index components. In these cases, the frailty index was imputed directly as a single continuous variable based on age band, gender, education years, self-perceived health, and living with or without spouse/partner as predictive factors. Besides the multiple imputation analyses, complete case analyses were also conducted to check the robustness of findings. In the complete case analyses, only surveys with available data on all frailty estimation survey items were included.

### Statistical analyses

Statistical analyses were conducted in R version 4.3.0 [52]. Data visualization was supported by the naniar (version 1.0.0) and the ggplot2 (version 3.4.2) packages [53, 54]. For the descriptive analysis of data after multiple imputation, the average of results in the imputed datasets were calculated. Exact confidence intervals for proportions were calculated using the PropCIs package, version 03–0 [55]. To predict frailty prevalence based on age, gender, and country GDP/capita, logistic regression analyses were conducted with fixed and random effects using the lme4 package, version 1.1–34 [56]. Gender and GDP per capita effects were investigated in interaction with age bands, since these effects were clearly age-dependent in descriptive analyses (see the “Results” section). The unique personal ID code of survey respondents was included in the regression models as a random clustering variable, to take into account the correlation between repeated measurements within subjects. Similarly, country effect was also included as a random factor, acknowledging that other country characteristics beyond the effect of GDP/capita could also influence the odds of developing frailty. GDP/capita was represented in the model as continuous variable expressed in thousand EUR, with or without purchasing power parity adjustment.

In the multiple imputation analyses, separate models were fitted for each imputed dataset and the model results were pooled as recommended by Rubin et al. [57]. The statistical analysis scripts are available in the Online Resource. Observed and model-predicted frailty rates in European countries are tabulated and visualized in the interactive SHARE Frailty Atlas for Europe (available at: https://bb-sri.shinyapps.io/share-vitalo/). The SHARE Frailty Atlas was developed using the R packages shiny (version 1.7.5) and sp (version 2.0–0) [58, 59].

## Results

Altogether 311,915 and 244,834 SHARE surveys could be included in frailty index and frailty instrument analyses, respectively (Fig. 1). The available data came from 29 SHARE participating countries spread across geographical Europe (Online Resource Table S2). However, data was not available for many other European countries including Albania, Belarus, Bosnia and Herzegovina, Iceland, Kosovo, Moldova, Montenegro, North Macedonia, Norway, Russia, Serbia, Ukraine, and the United Kingdom. The demographic characteristics of the study population are summarized in Table 1.

**Fig. 2:**
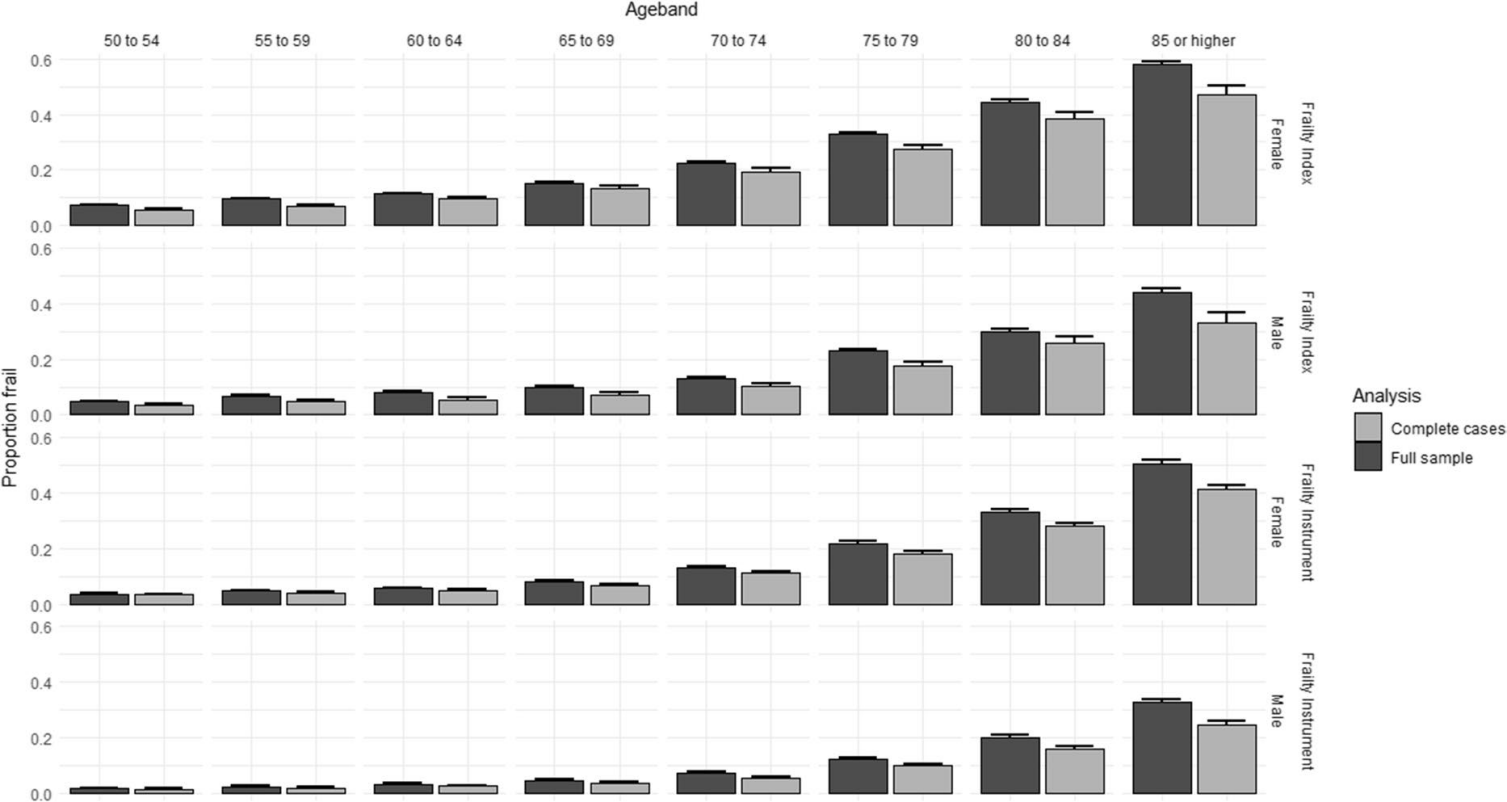
Observed frailty prevalence stratified by age, gender, frailty assessment method, and approach to deal with missing information (complete cases only, or full sample with multiple imputation)

**Fig. 3:**
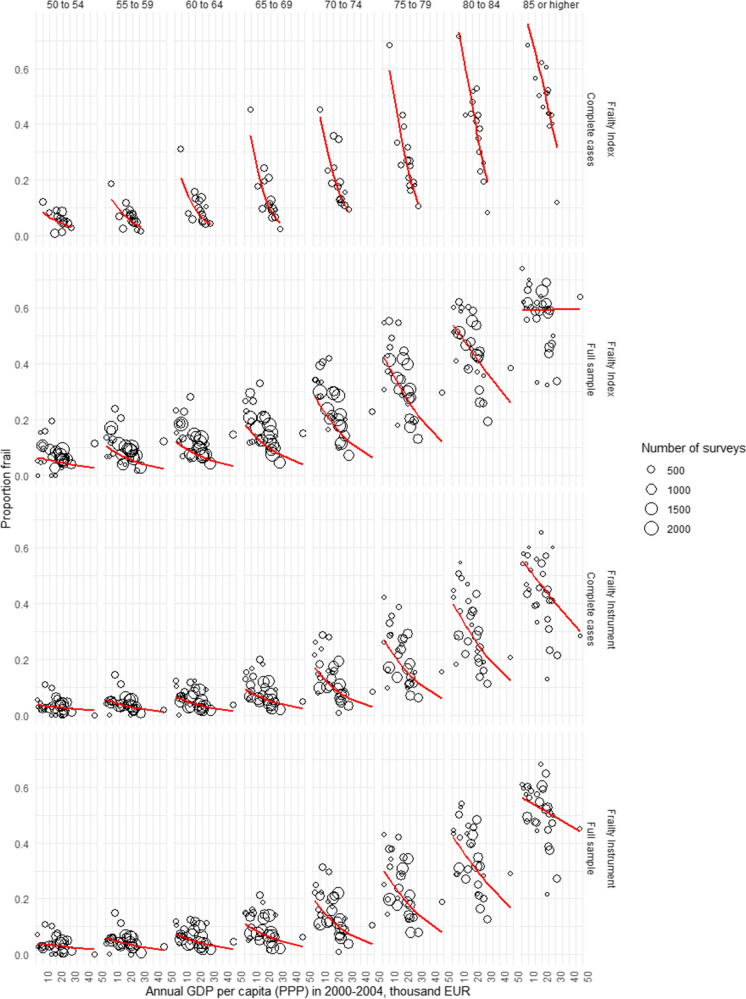
Frailty prevalence in females by age, country GDP per capita, frailty assessment method, and approach to deal with missing data. Circles indicate observed data, and model-fitted predictions are indicated by the red curve

**Fig. 4:**
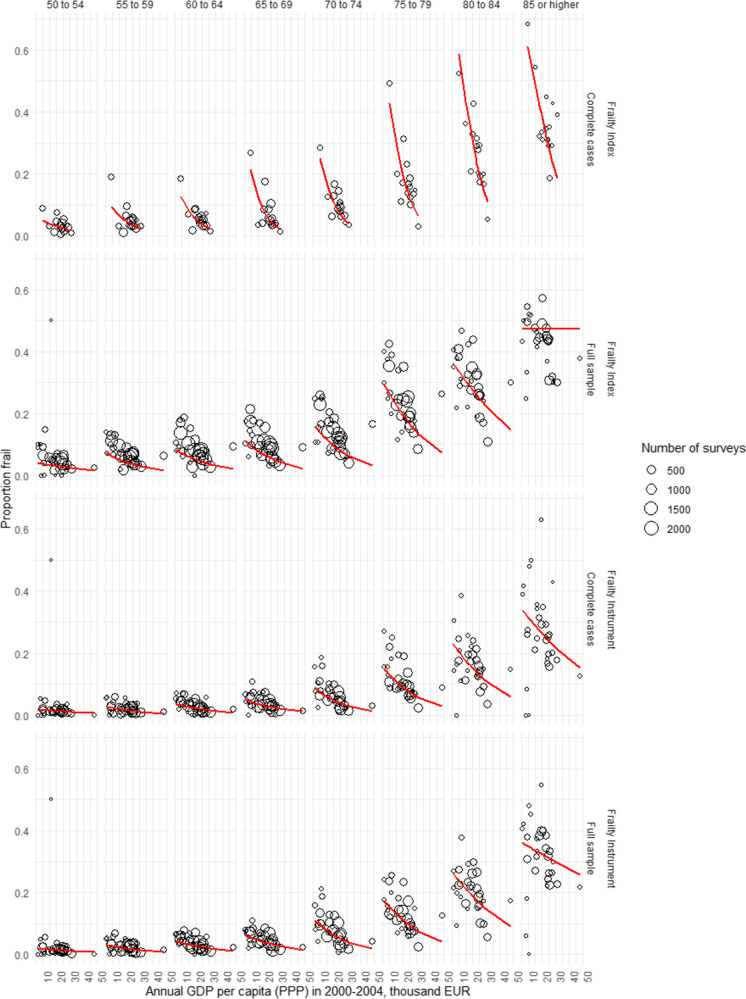
Frailty prevalence in males by age, country GDP per capita in thousands EUR, frailty assessment method, and approach to teal with missing data. Circles indicate observed data, and model-fitted predictions are indicated by the red curve

Missing data for frailty assessment occurred at significantly higher rates in higher age groups, and the rate of complete cases was significantly lower in survey respondents with positive response to a frailty instrument or frailty index variable (*p* < 0.00001 for all variables in chi^2^-test tests, Online Resource Table S1). Multiple imputation of missing variables allowed the inclusion of all eligible surveys with incomplete data for frailty assessment in our analyses. As expected, age- and gender-specific frailty rates tended to be higher in the full sample than in complete case analyses (Fig. 2). Results on age- and gender-specific frailty rates by country are tabulated and visualized on interactive maps in the SHARE Frailty Atlas for Europe. The association of observed age- and gender-specific frailty rates in SHARE participating countries with their GDP/capita with purchasing power parity adjustment is visualized in Figs. 3 and 4, indicating an interaction between age and gender. Gender difference in frailty rates was negligible at lower ages but became noticeable from age 65–70, with higher frailty rates in women. This interaction reached statistical significance in some age bands in multivariate regression models, as shown in Table 2. Interaction between age and the effect of GDP/capita was also noticed in the descriptive analyses, as frailty rates were very similar across all SHARE countries at lower ages (e.g., 50 to 54 years) but became more and more dependent on country GDP/capita at higher ages, reaching two to three times higher frailty rates in countries with lower GDP/capita than in countries with higher GDP/capita within the same age and gender strata (Figs. 3 and 4). Results on regression model coefficients are summarized in Table 2, and model-predicted frailty rates by age band, gender, and GDP/capita PPP values are shown in Figs. 3 and 4. The association of GDP/capita with frailty rates was statistically significant in multiple age bands, either in the frailty index or in the frailty instrument analyses and both in complete case and multiple imputation analyses, with strongest protective effect of higher GDP/capita against frailty in the eighth decade of life (Table 2). Very similar results were found when using GDP per capita data without PPP adjustment in the analyses (Online Resource Table S3 and Figure S1). For country-specific observed and predicted proportions, please visit the SHARE Frailty Atlas for Europe at https://bb-sri.shinyapps.io/share-vitalo/.

**Table 2:** Regression analysis results: effect of age, gender, and GDP/capita PPP on frailty prevalence in Europe

Multivariate model Fixed parameters	Frailty instrument, multiple imputation		Frailty instrument, complete cases		Frailty index, multiple imputation		Frailty index, complete cases	
	Coefficient	SE	Coefficient	SE	Coefficient	SE	Coefficient	SE
Intercept	− 3.0769***	0.2629	− 3.1681***	0.3055	− 2.5470***	0.2514	− 1.9103***	0.5348
Male gender	− 0.7756***	0.0906	− 0.7593***	0.1014	− 0.4979***	0.0619	− 0.5368***	0.1055
Age 55 to 59	0.5233**	0.1670	0.5335**	0.1868	0.6116***	0.1106	0.7235*	0.3055
Age 60 to 64	0.7197***	0.1609	0.7104***	0.1804	0.7450***	0.1107	1,3843***	0.3106
Age 65 to 69	1.1642***	0.1571	1.0823***	0.1768	1.2524***	0.1111	2.4387***	0.3106
Age 70 to 74	1.9076***	0.1545	1.8505***	0.0.1737	1.8574***	0.1112	2.5534***	0.3139
Age 75 to 79	2.4500***	0.1530	2.4107***	0.0.1728	2.4623***	0.1122	3.3674***	0.3254
Age 80 to 84	2.9310***	0.1544	2.9635***	0.1765	2.8603***	0.1165	4.0023***	0.3757
Age 85 +	3.3877***	0.1575	3.5119***	0.1873	2.9169***	0.1252	3.9215***	0.4798
Age 55 to 59: male	0.0879	0.1099	0.0234	0.1243	0.0918	0.0728	0.1620	0.1363
Age 60 to 64: male	0.2333*	0.1045	0.1464	0.1182	0.0741	0.0721	− 0.0562	0.1358
Age 65 to 69: male	0.1750	0.1017	0.0853	0.1153	− 0.0598	0.0719	− 0.1820	0.1351
Age 70 to 74: male	0.1192	0.1000	0.0087	0.1135	− 0.2329**	0.0719	− 0.2660*	0.1330
Age 75 to 79: male	0.0641	0.0988	0.0249	0.1122	− 0.0607	0.0717	− 0.1222	0.1355
Age 80 to 84: male	0.0627	0.0993	− 0.0331	0.1141	− 0.2213**	0.0741	− 0.1105	0.1458
Age 85 + : male	− 0.0456	0.1005	− 0.1148	0.1194	0.0174	0.0778	− 0.1670	0.1777
GDP/cap^#^	− 0.0160	0.0123	− 0.0195	0.0143	− 0.0200	0.0117	− 0.0483*	0.0229
Age 55 to 59: (GDP/cap^#^)	− 0.0153*	0.0077	− 0.0175*	0.0087	− 0.0172***	0.0050	− 0.0226	0.0132
Age 60 to 64: (GDP/cap)	− 0.0150*	0.0074	− 0.0158	0.0083	− 0.0131**	0.0049	− 0.0360**	0.0134
Age 65 to 69: (GDP/cap^#^)	− 0.0169*	0.0072	− 0.0152	0.0082	− 0.0189***	0.0050	− 0.0657***	0.0134
Age 70 to 74: (GDP/cap^#^)	− 0.0273***	0.0071	− 0.0264**	0.0080	− 0.0212***	0.0050	− 0.0488***	0.0135
Age 75 to 79: (GDP/cap^#^)	− 0.0211**	0.0070	− 0.0220**	0.0080	− 0.0190***	0.0050	− 0.0633***	0.0139
Age 80 to 84: (GDP/cap^#^)	− 0.0138	0.0071	− 0.0170*	0.0081	− 0.0078	0.0052	− 0.0644***	0.0160
Age 85 + : (GDP/cap^#^)	0.0048	0.0072	− 0.0052	0.0085	0.0202***	0.0056	− 0.0402*	0.0200

^#^Annual average in 2000–2004, in thousand EUR, adjusted for purchasing power parity; *p < 0.05; **p < 0.01; ***p < 0.001

## Discussion

### Comparison to previous data on frailty prevalence

Previous multi-country analyses of frailty prevalence in community-dwelling older adults in Europe were focusing on more developed EU Member States. Santos-Eggimann et al. [34] and Theou et al. [40] investigated SHARE Wave 1 datasets from 10 and 15 EU-15 countries, respectively. Both analyses described significant between-country heterogeneity within the investigated, more developed EU Member States with increasing frailty prevalence from North to South or by decreasing gross domestic product per capita, respectively. Interestingly, Harttgen et al. [42] found higher prevalence in higher income European countries when compared to non-European low-income countries, but within Europe, an increasing trend in mean frailty index was observed in less-developed countries, with lowest and highest mean frailty index in Denmark and Poland, respectively. A more recent analysis by Manfredi et al. presented frailty prevalence data from 18 European countries based on SHARE wave 6 data analysis, confirming the previously observed North to South gradient in Europe, with Poland as an outlier where frailty prevalence was clearly higher than expected based solely on its geographical position [60]. This study provided frailty prevalence data from a SHARE wave 6 analysis using the Fried phenotype definition, separately for the 50–64, 65–74, 75–84, and 85 + age bands. Notably, neither of the previous analyses did perform imputation exercise for missing frailty assessment items [34, 40, 60]. In the light of previous studies, the main contribution of our analysis is the provision of country-specific frailty prevalence estimates by narrow, 5-year age bands and by gender for all European countries, adopting both the Fried phenotype definition and the frailty index approach, and reporting frailty prevalence estimates either in complete cases analysis or after multiple imputation of missing data. The latter aspect is important because a higher rate of missing data was observed in higher age groups and in subjects with a positive response to another frailty-related survey items (Online Resource Table S1). Hence, we found that restricting the analyses to complete cases would result in biased estimates toward lower frailty prevalence. On the other hand, our analyses are based on previously established and operationalized frailty definitions that makes our frailty prevalence estimates in the complete cases analyses comparable and similar to previous SHARE data analysis findings on frailty prevalence by analysis design. Our results confirmed the previous country-level observation on the association of GDP per capita and frailty prevalence within Europe [40], and extended this analysis to a broader range of countries and to more up-to-date data from all relevant waves of SHARE, applying mixed logistic regression based on individual surveys, and analyzing this association by age bands and gender strata. The results were consistent when adopting two different frailty assessment methods, independently from the removal or multiple imputation of missing data. The association of frailty prevalence with GDP per capita was evident either when estimated the GDP using purchasing power parity (PPP) adjustment (Table 2, Figs. 3 and 4) or when estimated the GDP at market prices (Online Resource, Table S3 and Figure S1). PPP adjustment for GDP per capita is useful as a proxy for wealth in different countries since it reveals the actual volume of economic output (products and services) in any given country after corrections for price level differences between countries. Eurostat explicitly takes health service costs into account when estimating national PPP accounts [61]; hence, our findings with PPP adjustment may be more relevant. Analysis results without PPP adjustment were similar and are also provided as supplementary Online Resource (Table S3 and Figure S1).

The strongest association with GDP per capita was observed at the age of 65 to 79 years, showing significantly higher frailty prevalence in countries with lower GDP per capita in the reference years [42].

Notably, the analysis of the association of GDP per capita and frailty prevalence included country also as a random factor, ensuring that country characteristics other than GDP per capita resulting in lower within-country variance (e.g., population education status, health system characteristics) are taken into account in the analyses implicitly. The developed regression models allow for age- and gender-stratified frailty prevalence estimates in countries not covered by SHARE data collection. The advantage of developing a predictive regression model based on a low number of readily available predictors (age, gender, country GDP per capita) instead of building more precise but also more data intensive statistical models is pragmatic, to facilitate the use of the predictive tool in less-developed EU countries with lower research capacity and data infrastructure. The developed models allow for age band and gender specific prediction of frailty prevalence in European countries where local data is unavailable. The derived predicted frailty prevalence estimates are available in the online SHARE Frailty Atlas for Europe.

### Implications of frailty prevalence heterogeneity within Europe

From the geriatric research perspective, the better understanding of potential national differences in frailty patterns allows for the identification of countries with highest burden of frailty in the young-old population in Europe. Due to higher baseline risk of developing frailty, these countries are ideal candidates for hosting etiology and intervention studies on frailty which is not an inevitable fate but is a preventable and potentially treatable condition [62, 63]. Our analysis found that age- and gender-stratified frailty prevalence is particularly high in EU countries with low GDP per capita. Besides research efficiency considerations, these countries would deserve more attention also due to the relatively poor health status of their population. Better understanding of persisting health system deficits and failures in lower income European countries could be a logical research priority for the European Commission. Surprisingly, healthcare research funds allocated by the EU were found to be almost exclusively concentrated in former EU Member States (EU-15) during a recent 10-year period, while only 3.1% of the total grant amount was allocated to research participants from Central and Eastern European (CEE) Member States [64]. For comparison, about 20% of EU citizens reside in the latter countries. The under-representation of less-developed EU member States in EU funded health research projects is particularly alarming in the view of poorer health status, less local funds, and less human capacities for research in these countries. Accordingly, knowledge transfer from health research projects conducted in more mature health systems to lower income, less-developed EU countries should be increasingly encouraged and facilitated [64, 65]. This knowledge transfer exercise, also known as transferability research, needs to reflect on the dissimilarity of country population characteristics including age, sex, disease incidence/prevalence, disease severity, case-mix, education, socioeconomic status, co-morbidities, medical history, concurrent medications, susceptibility, lifestyle, risk factors, and life expectancy, among others [66]. Stratification of long-term outcomes and health economic modeling conclusions by age, gender, and frailty as a single surrogate of the above factors is a promising approach in this respect, decomposing complex differences in health status and risk factors across country populations to a limited number of measurable parameters. Hence, frailty is not solely a matter for geriatricians but should become a key factor in transferability research in all realms of medicine dealing with adult and elderly patients [67]. As an example, the Vaccines and Infectious disease in the Ageing Populations (VITAL) IMI2 project [68] investigates the burden of vaccine preventable diseases and the cost-effectiveness of various vaccination program scenarios in the elderly, stratifying the clinical study results and health economic modeling conclusions by age groups, gender, and frailty categories in exemplary countries or regions where good quality data is available with high granularity [69]. The aggregated, population-level results from these analyses have no direct relevance for other European countries with different population structure and characteristics. However, results for a specific stratum, e.g., for women who are frail and aged 80–84 years are more transferable between health systems, subject to availability of age- and gender-stratified data on frailty prevalence in the target countries and careful consideration of additional country heterogeneity factors including healthcare system characteristics (e.g., technology availability, practice variation, unit costs) and local decision frameworks [66]. Lack of comparable age- and gender-stratified data on frailty prevalence in European countries was an important bottleneck in this regard, and our study empowers researchers and health policy makers to have a more detailed understanding of the frailty burden within Europe, and to take it into consideration in the transferability assessment of cutting-edge research findings delivered in most developed countries to less-developed European regions.

### Strengths and limitations of our research

Strengths of our analysis include the exploitation of established standard frailty assessment methods and accumulated multinational data from several SHARE survey waves up to Wave 8; the parallel adoption of two alternative frailty assessment methods (the SHARE frailty instrument and a SHARE frailty index); the comparative analysis of complete cases only and all eligible surveys via multiple imputation; the adopted mixed model regression modeling approach of unique surveys, carefully controlling for lower variance within survey respondent and within countries; the derived country-level results on observed and predicted frailty rates, stratified by 5-year age bands and gender; and finally, the development of the SHARE Frailty Atlas for Europe which can become a valuable tool to provide researchers and health policymakers with stratified and locally relevant data on frailty rates in a user-friendly and interactive manner. Our findings are consistent across frailty assessment methods and approaches to handle missing data regarding the strong association of economic development of countries with frailty rates, confirming previous findings in a narrower country range using a 70-item frailty index [40]. The provided stratified frailty estimates may guide research policies and transferability research to close the health gap between more developed and less-developed European countries.

Key weaknesses of our research include the inherent limitations of frailty assessment due to the heterogeneity of operative definitions for frailty in the scientific literature, as overviewed in the Background section. Accordingly, two different, well-established operative definitions have been adopted in our analyses and the similarity of results using the frailty instrument and the frailty index approaches was reinforcing regarding the robustness of findings. However, it is emphasized that these frailty assessment approaches are not interchangeable and are considered complementary [11, 12]. Hence, numerical differences between frailty rates calculated using these approaches may reflect conceptual differences between these methods. User preferences among these methods may differ, subject to the specific research or policy context. Hence, results using both frailty assessment approaches are presented in parallel, without suggestions on their prioritization.

An additional limitation of our analysis was the relatively high rate of surveys with incomplete data for frailty assessment. The rate of complete cases was particularly low in the frailty index analyses, reflecting that in contrast to Frailty Instrument analyses where only seven SHARE variables were necessary for frailty assessment, more than 40 SHARE variables were necessary to calculate the frailty index (see Online Resource Table S1 for the included survey items). High rate of incomplete cases in the frailty index analyses was also due to the abandonment of two related SHARE survey items in Waves 5–8. Exclusion of incomplete cases from the analysis is a technically simple step but may generate selection bias when missingness occurs not at random [70, 71]. Missing data did not occur at random in our study, as incomplete frailty data was significantly more common in subgroups with higher risk of frailty: in subjects at higher chronological age, or with positive response to any frailty-related survey item (Online Resource Table S1). Hence, efforts were made to keep all eligible surveys in the analysis via multiple imputation. On the other hand, exclusion of complete survey waves with very high rate of missing data does neither remove nor enrich the surveys of high-risk respondents selectively and is not considered to introduce selection bias. Accordingly, SHARE waves 3, 5, and 7 were excluded from some or all analyses as indicated in Fig. 1. Recommendations in the literature on the optimal number of imputed datasets range from 3 to 20 or even more, with preference for increasing the number of imputed datasets when missing information in the data is higher [70, 72]. We decided to set the number of multiple imputations to 10, taking into account the size of the dataset and time constraints of computer calculations. In theory, a higher number of imputed datasets would provide even more precise parameter estimates—however, the statistical significance of several model parameters was already far beyond the significance threshold in our multiple imputation exercise (Table 2). Analysis results on complete cases and the multiple imputation datasets were consistent, confirming the robustness of results. The reader is advised to rely on the multiple imputation results, since these are based on a more comprehensive analysis of the available evidence and are confirmed by similar findings in complete case sensitivity analyses. Nevertheless, the complete case analysis results may be preferred in research or policy settings where the additional uncertainty introduced by the imputation process is strongly undesirable or unacceptable.

Developing frailty is a gradual process involving pre-frailty states that can already be differentiated from the healthy state. Intermediate health states between being healthy and frail can be identified using either the pre-frailty thresholds for the Frailty Instrument [34] or lower deficit accumulation thresholds when using a frailty index. Our analyses were exclusively focusing on the distinction between non-frail and frail states because binary assessment of frailty is known to be strongly associated with clinical outcomes [2, 3, 21, 22]. Exploration of epidemiology patterns of pre-frail health states within Europe was beyond our research aims.

An additional limitation of our research is that the developed predictive model was intentionally kept simple, to minimize input data requirements in countries without observed frailty epidemiology data. Reflecting the low number of independent variables considered in the fitted predictive models, a considerable variation around the predicted values was found in the observed values that potentially could be reduced by the inclusion of additional explanatory variables. Besides, there are many possible effect mediators between national economic development and age- and gender-stratified frailty rates, including lower education [13, 23–25], nutritional factors [26–28], physical inactivity [9, 30, 31], or smoking [9, 29]. For many of these factors, there are available SHARE survey items to be included in more complex analyses. However, the aim of our research was not to explore the complex interplay of potential effect mediators but to estimate frailty rate as an overall health status indicator in the elderly and to propose a simple but robust prediction approach with limited input data requirements that are consistently available for all European countries. More detailed analysis of frailty patterns along additional characteristics of survey respondents may be part of further research exploiting the SHARE database. Beside the comprehensive descriptive analysis of the available data, an important achievement of our research is the development of predictive models for age- and gender-specific frailty rates as a function of national economic development, surrogated by the GDP per capita. Development of age- and gender-stratified frailty rate predictions for countries with lack of local data is certainly only a second-best option, and the inclusion of currently not participating European countries into subsequent SHARE waves is strongly encouraged. Nevertheless, other priority settings and the shortage of financial and research capacities in countries with lower GDP/capita within Europe may remain a critical barrier to local data collection on frailty rates, and the developed prediction approach may guide research and policy assumptions on frailty prevalence in these countries until locally relevant observed data become available. Predictions for frailty rates in Ukraine and Russia have regrettably additional uncertainty due to the ongoing war between the two countries. Nevertheless, based on the low GDP/capita in Ukraine, development of frailty may start particularly early and may affect a remarkably large proportion in this country. European policies allocating more adequate resources via research grants and/or structural funds to better understand and improve health status and/or health system deficits in Eastern European countries within and beyond the European Union could start closing the apparently frozen health gap in the middle of the continent.

## Conclusions

Frailty is an aggregate health status indicator in the elderly, and the lack of comparative frailty prevalence data in several European countries, particularly in the economically less-developed ones, was an important blind spot in European frailty research. Our study provides age- and gender-stratified frailty prevalence estimates for all European countries, including observed values (where available from SHARE data collection) and multivariate model-based predictions that also take into account national economic development and other country characteristics (as a random clustering variable) besides age, gender, and repeated measurements within subjects. The synthetized evidence is accessible both in visual and tabular format in the SHARE Frailty Atlas for Europe. Age- and gender-specific frailty prevalence in Europe shows remarkable between-country heterogeneity. Higher frailty prevalence is strongly associated with lower GDP per capita, especially in the 65–79 age group. This finding was consistent across frailty index and SHARE frailty instrument based frailty assessment methods, both in complete cases and multiple imputation analyses and with or without purchasing power parity adjustment of GDP per capita values. Our findings will empower transferability researchers to adopt frailty as a single measurable surrogate of dissimilarity of country population characteristics, and aim to support the refinement of European policies on health system development, research grants and structural funds to start closing the insistent health gap within European countries of higher and lower economic development. Focusing on less-developed European countries with higher prevalence and earlier onset of frailty can be a low-hanging fruit for geriatric research groups to investigate risk factors and preventive interventions for frailty in high-risk populations.

### Supplementary Information

Below is the link to the electronic supplementary material.

Supplementary file1 (PDF 975 KB)

## Data Availability

Access to SHARE data is subject to SHARE user requirements and eligibility rules (https://www.share-eric.eu/data/data-access/conditions-of-use). All other data came from public sources as described in the “Methods” section.

## Abbreviations

*CEE*: Central and Eastern European
*EU*: European Union
*GDP*: Gross domestic product
*PPP*: Purchasing power parity
