# Supplementary material for: Frailty prevalence in 42 European countries by age and gender: development of the SHARE Frailty Atlas for Europe
Source: GeroScience. 2023 Oct 19;46(2):1807–24. doi: 10.1007/s11357-023-00975-3 (PMC10828249; doi:10.1007/s11357-023-00975-3)
Supplement: Supplementary file 1 — Supplementary file1 (PDF 975 KB) [file 11357_2023_975_MOESM1_ESM.pdf]

Frailty prevalence in 42 European countries by age and gender: development of the SHARE Frailty Atlas for Europe

Online resource as supplementary material to manuscript in

Geroscience

János G. Pitter[1,2], Antal Zemplényi[1,2], Balázs Babarczy[1], Bertalan Németh1, Zoltán Kaló1,3, Zoltán Vokó1,3*

1 Syreon Research Institute, Budapest, Hungary; 2 University of Pécs, Faculty of Pharmacy, Center for Health Technology Assessment and Pharmacoeconomic Research, Pécs, Hungary; 3 Semmelweis University, Center for Health Technology Assessment, Budapest, Hungary.

*Corresponding author; voko.zoltan@semmelweis.univ.hu

Table of contents

- **Table S1. Frailty assessment variables and data missingness** **2**
- **Table S2. Study population by country** **8**
- **Table S3. Regression analysis results: effect of age, gender, and GDP/capita PPP on frailty prevalence in Europe** **9**
- **Figure S1A. Frailty prevalence in females by age, country GDP per capita PPP (in thousands EUR), frailty assessment method, and approach to deal with missing data.** **10**
- **Figure S1B. Frailty prevalence in males by age, country GDP per capita PPP in thousands EUR, frailty assessment method, and approach to teal with missing data.** **11**
- **Detailed data sources and methods of purchasing power parity adjustment** **12**
- **Statistical analysis script for the Frailty Index analyses** **17**
- **Statistical analysis script for the Frailty Instrument analyses** **46**

Table S1. Frailty assessment variables and data missingness

Frailty Instrument analyses					
Data source	Parameter	Value	Number (% total)	Surveys with missing data in (other) frailty items: Number (%)	Surveys with missing data in (other) frailty items: Chi-squared test p
age_int, age_2004, age_2007, age_2011 age_2013, age_2015 age_2017, age_2020 yrbirth, mobirth int_year, int_month	Ageband	50-54	26012 (10.62%)	1590 (6.11%)	< 2.2 E-16
		55-59	37950 (15.50%)	2678 (7.06%)	
		60-64	41956 (17.14%)	3181 (7.58%)	
		65-69	40326 (16.47%)	3624 (8.99%)	
		70-74	34695 (14.17%)	3914 (11.28%)	
		75-79	27770 (11.34%)	4460 (16.06%)	
		80-84	19827 (8.10%)	4909 (24.76%)	
		85-89	10786 (4.41%)	4135 (38.34%)	
		90+	5512 (2.25%)	3446 (62.52%)	
		Missing	0		
gender	Gender	Male	108850 (44.46%)	13716 (12.60%)	5.75 E-9
		Female	135984 (55.54%)	18221 (13.40%)	
		Missing	0		
mh013_	Fatigue	No	147580 (60.28%)	7624 (5.20%)	< 2.2 E-16
		Yes	81606 (33.33%)	8615 (10.56%)	
		Missing	15648 (6.39%)		
mh011_ mh012_	Loss of appetite	No	208568 (85.19%)	13371 (6.41%)	< 2.2 E-16
		Yes	21043 (8.59%)	3343 (15.89%)	
		Missing	15223 (6.22%)		
maxgrip	Gripstregth	Data available	214122 (87.46%)	1225 (0.57%)	NA
		Missing	30712 (12.54%)		
ph048d1 ph048d5	Slowness	No	192395 (78.58%)	11699 (6.08%)	< 2.2 E-16
		Yes	42195 (17.23%)	9994 (23.69%)	
		Missing	10244 (4.18%)		

br016_	Low / moderate physical activity	More than once a week	155704 (63.6%)	8036 (5.16%)	< 2.2 E-16
		Once a week	31906 (13.03%)	2474 (7.75%)	
		One to three times a month	14181 (5.79%)	1529 (10.78%)	
		Hardly ever or never	32139 (13.13%)	8994 (27.98%)	
		Missing	10904 (4.45%)		

**Frailty Index analyses**					
**Data source**	**Parameter**	**Value**	**Number (% total)**	**Surveys with missing data in (other) frailty items**	
				**Number (%)**	**Chi-squared test p**
age_int, age_2004, age_2007, age_2011 age_2013, age_2015 age_2017, age_2020 yrbirth, mobirth int_year, int_month	Ageband	50-54	33562 (10.76%)	22093 (65.83%)	< 2.2 E-16
		55-59	48710 (15.62%)	36257 (74.43%)	
		60-64	53715 (17.22%)	42334 (78.81%)	
		65-69	51493 (16.51%)	41914 (81.40%)	
		70-74	43882 (14.07%)	35997 (82.03%)	
		75-79	35073 (11.24%)	29508 (84.13%)	
		80-84	25029 (8.02%)	21681 (86.62%)	
		85-89	13669 (4.38%)	12468 (91.21%)	
		90+	6782 (2.17%)	6411 (94.53%)	
		Missing	0	0	
gender	Gender	Male	138966 (44.55%)	109606 (78.87%)	< 2.2 E-16
		Female	172949 (55.45%)	139057 (80.40%)	
		Missing	0	0	
ph049d3	Index 01	No	280063 (89.79%)	219044 (78.21%)	< 2.2 E-16
		Yes	19206 (6.16%)	16973 (88.37%)	
		Missing	12646 (4.05%)		
ph049d1	Index 02	No	274386 (87.97%)	214599 (78.21%)	< 2.2 E-16
		Yes	24883 (7.98%)	21418 (86.07%)	
		Missing	12646 (4.05%)		

ph048d3	Index 03	No	240050 (76.96%)	187297 (78.02%)	< 2.2 E-16
		Yes	59248 (18.99%)	48749 (82.28%)	
		Missing	12617 (4.05%)		
ph049d3	Index 04	No	291302 (93.39%)	228742 (78.52%)	< 2.2 E-16
		Yes	7967 (2.55%)	7275 (91.31%)	
		Missing	12646 (4.05%)		
ph049d2	Index 05	No	292497 (93.77%)	229776 (78.56%)	< 2.2 E-16
		Yes	6772 (2.17%)	6241 (92.16%)	
		Missing	12646 (4.05%)		
ph049d4	Index 06	No	269672 (86.46%)	210693 (78.13%)	< 2.2 E-16
		Yes	29626 (9.50%)	25353 (85.58%)	
		Missing	12617 (4.05%)		
ph048d7	Index 07	No	290822 (93.24%)	228311 (78.51%)	< 2.2 E-16
		Yes	8447 (2.71%)	7706 (91.23%)	
		Missing	12646 (4.05%)		
ph049d6	Index 08	No	258560 (82.89%)	201371 (77.88%)	< 2.2 E-16
		Yes	40738 (13.06%)	34675 (85.12%)	
		Missing	12617 (4.05%)		
ph048d5	Index 09	No	233668 (74.91%)	181354 (77.61%)	< 2.2 E-16
		Yes	65630 (21.04%)	54692 (83.33%)	
		Missing	12617 (4.05%)		
ph048d9	Index 10	No	277189 (88.87%)	216500 (78.11%)	< 2.2 E-16
		Yes	22080 (7.08%)	19517 (88.39%)	
		Missing	12646 (4.05%)		
ph049d12	Index 11	No	262254 (84.08%)	204350 (77.92%)	< 2.2 E-16
		Yes	37015 (11.87%)	31667 (85.55%)	
		Missing	12646 (4.05%)		
ph049d8	Index 12	No	285071 (91.39%)	223209 (78.30%)	< 2.2 E-16
		Yes	14198 (4.55%)	12808 (90.21%)	
		Missing	12646 (4.05%)		
ph049d11	Index 13	No	291709 (93.52%)	228963 (78.49%)	< 2.2 E-16

		Yes	7560 (2.42%)	7054 (93.31%)	
		Missing	12646 (4.05%)		
ph049d13	Index 14	No	284186 (91.11%)	222437 (78.27%)	< 2.2 E-16
		Yes	15083 (4.84%)	13580 (90.04%)	
		Missing	12646 (4.05%)		
ph048d1	Index 15	No	264696 (84.86%)	206264 (77.92%)	< 2.2 E-16
		Yes	34602 (11.09%)	29782 (86.07%)	
		Missing	12617 (4.05%)		
ph049d5	Index 16	No	286262 (91.78%)	224562 (78.45%)	< 2.2 E-16
		Yes	13007 (4.17%)	11455 (88.07%)	
		Missing	12646 (4.05%)		
br015_ br016_	Index 17	No	261620 (83.88%)	203878 (77.93%)	< 2.2 E-16
		Yes	36930 (11.84%)	5510 (85.08%)	
		Missing	13365 (4.28%)		
mh011_ mh012_	Index 18	No	267064 (85.62%)	208580 (78.10%)	< 2.2 E-16
		Yes	26343 (8.45%)	21575 (81.90%)	
		Missing	18508 (5.93%)		
sphus	Index 19	Excellent	22135 (7.10%)	15764 (71.22%)	< 2.2 E-16
		Very good	51987 (16.67%)	38814 (74.66%)	
		Good	109140 (34.99%)	84762 (77.66%)	
		Fair	82893 (26.58%)	68163 (82.23%)	
		Poor	33129 (10.62%)	28529 (86.11%)	
		Missing	12631 (4.05%)		
ph004_	Index 20	No	142686 (45.75%)	109221 (76.55%)	< 2.2 E-16
		Yes	156696 (50.24%)	126909 (80.99%)	
		Missing	12533 (4.02%)		
mh013_	Index 21	No	188319 (60.38%)	144526 (76.75%)	< 2.2 E-16
		Yes	104498 (33.50%)	85039 (81.38%)	
		Missing	19098 (6.12%)		

mh002_	Index 22	No	176862 (56.70%)	136482 (77.17%)	< 2.2 E-16
		Yes	116056 (37.21%)	93184 (80.29%)	
		Missing	18997 (6.09%)		
mh016_	Index 23	No	254028 (81.44%)	198758 (78.24%)	4.181 E-06
		Yes	38523 (12.35%)	30541 (79.28%)	
		Missing	19364 (6.21%)		
mh003_	Index 24	No	240971 (77.26%)	186154 (77.25%)	< 2.2 E-16
		Yes	51260 (16.43%)	42825 (83.54%)	
		Missing	19684 (6.31%)		
ph006d2	Index 25	No	179709 (57.61%)	137884 (76.73%)	< 2.2 E-16
		Yes	119500 (38.31%)	98073 (82.07%)	
		Missing	12706 (4.07%)		
ph006d1	Index 26	No	261583 (83.86%)	205891 (78.71%)	1.071 E-07
		Yes	37626 (12.06%)	30066 (79.91%)	
		Missing	12706 (4.07%)		
ph006d4	Index 27	No	286951 (92.00%)	225719 (78.66%)	< 2.2 E-16
		Yes	12258 (3.93%)	10238 (83.52%)	
		Missing	12706 (4.07%)		
ph006d10	Index 28	No	283812 (90.99%)	223564 (78.77%)	3.887 E-07
		Yes	15397 (4.94%)	12393 (80.49%)	
		Missing	12706 (4.07%)		
ph006d5	Index 29	No	260428 (83.49%)	203747 (78.24%)	< 2.2 E-16
		Yes	38781 (12.43%)	32210 (83.06%)	
		Missing	12706 (4.07%)		
ph006d8 (Waves 1-4); ph006d19 & ph006d20 (Waves 5-8)	Index 30	No	227832 (73.04%)	176687 (77.55%)	< 2.2 E-16
		Yes	71377 (22.88%)	59270 (83.04%)	
		Missing	12706 (4.07%)		
ph006d6	Index 31	No	280826 (90.03%)	220746 (78.61%)	< 2.2 E-16
		Yes	18383 (5.89%)	15211 (82.74%)	

		Missing	12706 (4.07%)		
ph006d9	Index 32	No	68961 (22.11%)	10499 (15.22%)	< 2.2 E-16
		Yes	6152 (1.97%)	1362 (22.14%)	
		Missing	236802 (75.92%)		
ph006d14	Index 33	No	293181 (93.99%)	231086 (78.82%)	0.0001974
		Yes	6028 (1.93%)	4871 (80.81%)	
		Missing	12706 (4.07%)		
orienti	Index 34	No	200353 (64.23%)	145345 (72.54%)	< 2.2 E-16
		Yes	32999 (10.58%)	24755 (75.02%)	
		Missing	78563 (25.19%)		
bmi	Index 35	0 (18.5 to 24.999)	104136 (33.39%)	80367 (77.18%)	< 2.2 E-16
		0.5 (25 to 29.999)	120696 (38.70%)	93569 (77.52%)	
		1 (<18.5 or 30+)	65364 (20.96%)	53008 (81.10%)	
		Missing	21719 (6.96%)		
ph010d3	Index 36	No	104993 (33.66%)	49070 (46.74%)	< 2.2 E-16
		Yes	16298 (5.23%)	8969 (55.03%)	
		Missing	190624 (61.11%)		
ph010d7 (Waves 1-4); ph089d1 (Waves 5-8)	Index 37	No	279216 (89.52%)	218139 (78.13%)	< 2.2 E-16
		Yes	20025 (6.42%)	17850 (89.14%)	
		Missing	12674 (4.06%)		
ph010d8 (Waves 1-4); ph089d2 (Waves 5-8)	Index 38	No	263880 (84.60%)	205057 (77.71%)	< 2.2 E-16
		Yes	35361 (11.34%)	30932 (87.47%)	
		Missing	12674 (4.06%)		
ph010d9 (Waves 1-4); ph089d3 (Waves 5-8)	Index 39	No	258555 (82.89%)	200872 (77.69%)	< 2.2 E-16
		Yes	40686 (13.04%)	35117 (86.31%)	
		Missing	12674 (4.06%)		
maxgrip	Index 40	No	233773 (74.95%)	178552 (76.38%)	< 2.2 E-16
		Yes	38858 (12.46%)	30827 (79.33%)	
		Missing	39284 (12.59%)		

Table S2. Study population by country

Country*	Frailty Instrument analyses (No. of surveys / No. of subjects)		Frailty Index analyses (No. of surveys / No. of subjects)	
	full sample	complete cases	full sample	complete cases
Austria	12,911 / 6,075	10,883 / 5,503	17,279 / 6,137	5,879 / 5,003
Belgium	20,220 / 9,157	18,406 / 8,744	25,900 / 9,569	7,818 / 5,317
Bulgaria	986 / 986	835 / 835	986 / 986	0
Croatia	3,782 / 2,604	3,291 / 2,371	3,782 / 2,604	0
Cyprus	574 / 574	478 / 478	574 / 574	0
Czech Republic	16,164 / 8,116	14,182 / 7,567	21,915 / 8,423	2,416 / 2,416
Denmark	12,683 / 5,381	11,664 / 5187	16,872 / 5,713	3,855 / 2,786
Estonia	16,127 / 7,624	13,493 / 7,038	22,145 / 7,674	0
Finland	11,84 / 1,184	1,115 / 1,115	1,184 / 1,184	0
France	18,226 / 8,051	15,963 / 7,498	22,793 / 8,137	4,988 / 3,439
Germany	14,662 / 7,538	13,214 / 7,010	20,275 / 8,617	4,935 / 3,575
Greece	14,319 / 6,468	12,128 / 5,978	14,319 / 6,468	5,126 / 3,235
Hungary	3,946 / 3,030	3,411 / 2,825	3,946 / 3,030	0
Ireland	1,007 / 1,007	791 / 791	1,007 / 1,007	778 / 778
Israel	8,172 / 3,629	6,245 / 3,248	10,928 / 3,842	3,622 / 2,508
Italy	16,860 / 7,853	14,074 / 7,031	21,661 / 8,358	4,676 / 3,266
Latvia	823 / 823	684 / 684	823 / 823	0
Lithuania	1,523 / 1,523	1,358 / 1,358	1,523 / 1,523	0
Luxembourg	2,534 / 1,708	2,238 / 1,545	4,121 / 2,116	0
Malta	839 / 839	723 / 723	839 / 839	0
Netherlands	10,406 / 5,454	9,591 / 5,159	14,607 / 6,491	5,774 / 4,185
Poland	8,598 / 4,474	7,221 / 4,001	8,598 / 4,474	2,151 / 2,151
Portugal	3,804 / 2,144	3,150 / 1,970	3,804 / 2,144	0
Romania	1,384 / 1,384	1,177 / 1,177	1,384 / 1,384	0
Slovakia	1,029 / 1,029	878 / 878	1,029 / 1,029	0
Slovenia	9,739 / 5,219	8,439 / 4,835	12,705 / 5,384	0
Spain	16,836 / 8,012	13,850 / 7,043	23,667 / 8,668	3,785 / 2,670
Sweden	14,524 / 6,084	13,118 / 5,781	19,226 / 6,615	5,203 / 3,397
Switzerland	10,972 / 4,488	10,297 / 4,376	14,023 / 4,515	2,246 / 1,616
Total	244,834 / 122,458	212,897 / 112,749	311,915 / 128,328	63,252 / 46,342

*No data collection in Albania, Belarus, Bosnia and Herzegovina, Iceland, Kosovo, Moldova, Montenegro, North Macedonia, Norway, Russia, Serbia, Ukraine, and the United Kingdom.

Table S3. Regression analysis results: effect of age, gender, and GDP/capita on frailty prevalence in Europe

Model parameter	Frailty Instrument, multiple imputation		Frailty Instrument, complete cases		Frailty Index, multiple imputation		Frailty Index, complete cases	
	Coefficient	SE	Coefficient	SE	Coefficient	SE	Coefficient	SE
Intercept	-3.0769 ***	0.2629	-3.2068 ***	0.2154	-2.6289 ***	0.1745	-2.4057 ***	0.3493
Male gender	-0.7756 ***	0.0906	-0.7607 ***	0.1014	-0.4989 ***	0.0619	-0.5396 ***	0.1055
Age 55 to 59	0.5233 **	0.1670	0.3930 **	0.1265	0.4780 ***	0.0784	0.3726	0.1970
Age 60 to 64	0.7197 ***	0.1609	0.5866 ***	0.1222	0.6528 ***	0.0783	0.9896 ***	0.1980
Age 65 to 69	1.1642 ***	0.1571	1.9633 ***	0.1195	1.1370 ***	0.0784	1.8390 ***	0.1979
Age 70 to 74	1.9076 ***	0.1545	1.6656 ***	0.1174	1.7222 ***	0.0784	2.1003 ***	0.1981
Age 75 to 79	2.4499 ***	0.1530	2.2415 ***	0.1168	2.3364 ***	0.0790	2.7415 ***	0.2046
Age 80 to 84	2.9310 ***	0.1544	2.8286 ***	0.1193	2.8495 ***	0.0819	3.3793 ***	0.2293
Age 85+	3.3877 ***	0.575	3.4619 ***	0.1271	3.1178 ***	0.0874	3.6074 ***	0.2883
Age 55 to 59: male	0.0879	0.1099	0.0248	0.1243	0.0930	0.0728	0.1635	0.1363
Age 60 to 64: male	0.2333 *	0.1045	0.1477	0.1182	0.0751	0.0721	-0.0533	0.1358
Age 65 to 69: male	0.1750	0.1017	0.0866	0.1153	-0.0588	0.0719	-0.1784	0.1351
Age 70 to 74: male	0.1192	0.1000	0.0104	0.1135	-0.2317 **	0.0719	-0.2634 *	0.1330
Age 75 to 79: male	0.0641	0.0988	0.0259	0.1122	-0.0598	0.0717	-0.1205	0.1355
Age 80 to 84: male	0.0627	0.0993	-0.0318	0.1140	-0.2193 **	0.0741	-0.1080	0.1458
Age 85+: male	-0.0456	0.1005	-0.1109	0.1194	0.0212	0.0777	-0.1678	0.1779
GDP/cap PPP#	-0.0160	0.0123	-0.0173	0.0092	-0.0158 *	0.0074	-0.0243	0.0131
Age 55 to 59: (GDP/cap PPP#)	-0.0153 *	0.0077	-0.0106 *	0.0054	-0.0150 ***	0.0032	-0.0064	0.0076
Age 60 to 64: (GDP/cap PPP#)	-0.0150 *	0.0074	-0.0097	0.0052	-0.0085 **	0.0031	-0.0173 *	0.0076
Age 65 to 69: (GDP/cap PPP#)	-0.0169 *	0.0072	-0.0093	0.0051	-0.0131 ***	0.0031	-0.0369 ***	0.0077
Age 70 to 74: (GDP/cap PPP#)	-0.0273 ***	0.0071	-0.0175 ***	0.0050	-0.0145 ***	0.0032	-0.0269 ***	0.0076
Age 75 to 79: (GDP/cap PPP#)	-0.0211 **	0.0070	-0.0137 **	0.0050	-0.0127 ***	0.0032	-0.0333 ***	0.0078
Age 80 to 84: (GDP/cap PPP#)	-0.0138	0.0071	-0.0104 *	0.0050	-0.0072 *	0.0033	-0.0345 ***	0.0086
Age 85+: (GDP/cap PPP#)	0.0048	0.0072	-0.0030	0.0052	0.0101 **	0.0035	-0.0243 *	0.0105

annual average in 2000-2004, in thousand EUR; *p<0.05; **p<0.01; ***p<0.001

Figure S1A. Frailty prevalence in **females** by age, country GDP per capita (in thousands EUR), frailty assessment method, and approach to deal with missing data.

Circles indicate observed data, and model-fitted predictions are indicated by the red curve.

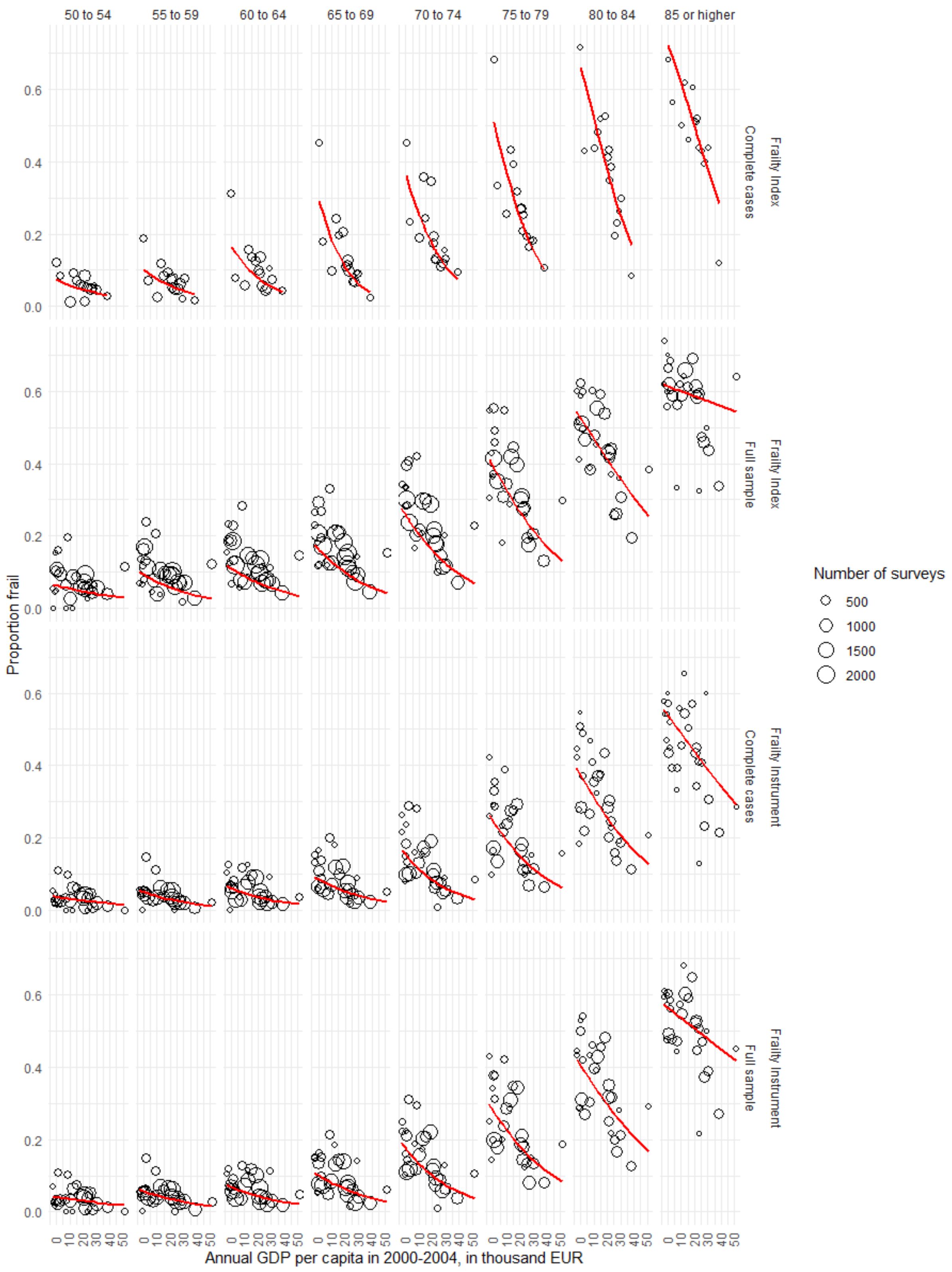

Figure S1B. Frailty prevalence in **males** by age, country GDP per capita in thousands EUR, frailty assessment method, and approach to teal with missing data. *Circles indicate observed data, and model-fitted predictions are indicated by the red curve.*

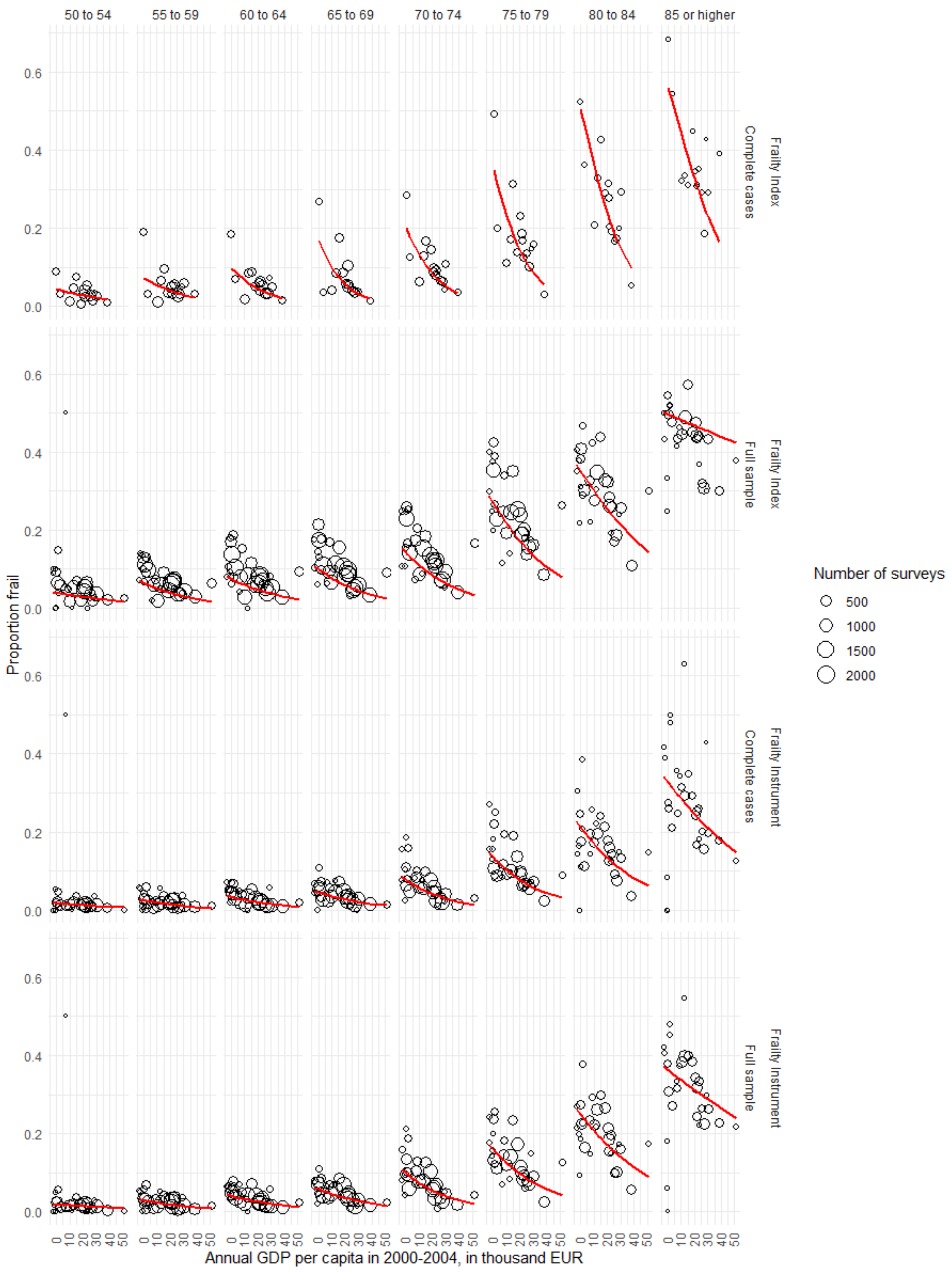

Detailed data sources and methods of purchasing power parity adjustment

Purchasing power adjusted GDP per capita data was downloaded from EUROSTAT [variable SDG_10_10] in the form of annual volume indices of real expenditure per capita, expressed as percentages of average EU-27 real expenditure per capita in each country and calendar year. Multiplying these indices with annual GDP per capita of the EU-27 [obtained from EUROSTAT, variable NAMA_10_PC] resulted in country- and calendar year- specific purchasing power adjusted GDP per capita estimates. These estimates were averaged across five calendar years preceding SHARE data collection (2000-2004) to compensate for annual fluctuation.

In countries without relevant EUROSTAT data, purchasing power parity adjustment was based on World Bank datasets. First, purchasing power of 1 USD was determined in the country as the ratio of GDP per capita in USD PPP and GDP per capita in USD, the corresponding data sources were: https://data.worldbank.org/indicator/NY.GDP.PCAP.PP.CD?end=2004&start=2000, and https://data.worldbank.org/indicator/NY.GDP.PCAP.CD?end=2004&start=2000], respectively. Second, purchasing power of 1 USD in the EU-27 was also determined as the ratio of EU-27 GDP per capita in USD PPP and GDP per capita in USD, using the same data sources. Then the ratio of the purchasing powers in the specific country and in the EU-27 was calculated and multiplied by GDP per capita in EUR to get a purchasing power parity adjusted GDP per capita estimate in the specific country and year. GDP per capita in EUR in theyse countries was also calculated from World Bank data (on GDP per capita in USD) and annual average USD / EUR exchange rates of the European Central Bank, available at https://www.ecb.europa.eu/stats/policy_and_exchange_rates/euro_reference_exchange_rates/html/eurofxref-graph-usd.en.html. For quality assurance of these calculations, the estimated values were compared with existing EUROSTAT data in two test countries, HU and NL and excellent agreement was found between EUROSTAT reported and World Bank data derived GDP per capita PPP estimates (see below). In countries without relevant EUROSTAT data, the calculated estimates were also averaged across five calendar years preceding SHARE data collection (2000-2004) to compensate for annual fluctuation.

Test country #1: Hungary					
	2000	2001	2002	2003	2004
GDP/cap in USD	4624.281657	5276.033189	6655.333009	8419.430871	10301.70339
GDP/cap in USD PPP	11854.80221	13210.00097	14526.38826	15481.98907	16272.09176
**purchasing power of 1 USD in Hungary**	$2.56	$2.50	$2.18	$1.84	$1.58
EU GDP/cap in USD.	$16,947.60	$17,199.12	$18,760.98	$22,967.49	$26,307.61
EU GDP/cap in USD PPP	$22,076.90	$23,171.60	$24,186.93	$24,707.28	$25,863.67
**purchasing power of 1 USD in the EU**	$1.30	$1.35	$1.29	$1.08	$0.98
**proportion of PPP HU/EU**	1.97	1.86	1.69	1.71	1.61
**USD / EUR annual exchange rate**	0.9236	0.90 €	0.9456	1.1308	1.2440
GDP per capita PPS, current EUR (calculated)	9,853.27 €	10,948.10 €	11,915.85 €	12,727.10 €	13,304.98 €

GDP per capita PPS, current EUR (EUROSTAT)	9,919.80 €	10,955.40 €	11,922.00 €	12,604.60 €	13,343.40 €

Test country #2: Netherlands					
	2000	2001	2002	2003	2004
GDP/cap in USD	26214.49855	26896.54811	29343.245	35750.97466	40436.61823
GDP/cap in USD PPP	31875.20022	33185.38942	34447.20532	34151.09523	35809.9829
**purchasing power of 1 USD in the Netherlands**	$1.22	$1.23	$1.17	$0.96	$0.89
EU GDP/cap in USD	$16,947.60	$17,199.12	$18,760.98	$22,967.49	$26,307.61
EU GDP/cap in USD PPP	$22,076.90	$23,171.60	$24,186.93	$24,707.28	$25,863.67
**purchasing power of 1 USD in the EU**	$1.30	$1.35	$1.29	$1.08	$0.98
**proportion of PPP NL/EU**	0.93	0.92	0.91	0.89	0.90
**USD / EUR annual exchange rate**	0.9236	0.90 €	0.9456	1.1308	1.2440
GDP per capita PPS, current EUR (calculated)	26,493.48 €	27,503.18 €	28,256.70 €	28,074.20 €	29,280.26 €
GDP per capita PPS, current EUR (EUROSTAT)	26,452.80 €	27,484.60 €	28,215.40 €	28,055.40 €	29,228.40 €

Belarus					
	2000	2001	2002	2003	2004
GDP/cap in USD	1276.288034	1244.373185	1479.314583	1819.766059	2378.623286
GDP/cap in USD PPP	5801.75813	6244.747744	6704.592279	7369.845906	8491.894839
**purchasing power of 1 USD in Belarus**	$4.55	$5.02	$4.53	$4.05	$3.57
EU GDP/cap in USD	$16,947.60	$17,199.12	$18,760.98	$22,967.49	$26,307.61
EU GDP/cap in USD PPP.	$22,076.90	$23,171.60	$24,186.93	$24,707.28	$25,863.67
**purchasing power of 1 USD in the EU**	$1.30	$1.35	$1.29	$1.08	$0.98
**proportion of PPP Belarus/EU**	3.49	3.72	3.52	3.76	3.63
**USD / EUR annual exchange rate**	0.9236	0.90 €	0.9456	1.1308	1.2440
GDP per capita PPS, current EUR (calculated)	4,822.21 €	5,175.48 €	5,499.71 €	6,058.45 €	6,943.45 €

Bosnia and Herzegovina					
	2000	2001	2002	2003	2004
GDP/cap in USD	1332.122365	1382.80542	1602.695144	2031.322775	2451.821681
GDP/cap in USD PPP	4127.638087	4290.625762	4653.131647	4911.659964	5405.608803

**purchasing power of 1 USD in Bosnia**	$3.10	$3.10	$2.90	$2.42	$2.20
EU GDP/cap in USD	$16,947.60	$17,199.12	$18,760.98	$22,967.49	$26,307.61
EU GDP/cap in USD PPP	$22,076.90	$23,171.60	$24,186.93	$24,707.28	$25,863.67
**purchasing power of 1 USD in the EU**	$1.30	$1.35	$1.29	$1.08	$0.98
**proportion of PPP Bosnia/EU**	2.38	2.30	2.25	2.25	2.24
**USD / EUR annual exchange rate**	0.9236	0.90 €	0.9456	1.1308	1.2440
GDP per capita PPS, current EUR (calculated)	3,430.74 €	3,555.96 €	3,816.92 €	4,037.67 €	4,419.93 €

Israel					
	2000	2001	2002	2003	2004
GDP/cap in USD	21630.74761	20909.43036	19035.10241	19627.17549	20557.07863
GDP/cap in USD PPP	25012.03846	25010.55066	25230.01157	23849.82372	25236.93936
**purchasing power of 1 USD in Israel**	$1.16	$1.20	$1.33	$1.22	$1.23
EU GDP/cap in USD	$16,947.60	$17,199.12	$18,760.98	$22,967.49	$26,307.61
EU GDP/cap in USD PPP	$22,076.90	$23,171.60	$24,186.93	$24,707.28	$25,863.67
**purchasing power of 1 USD in the EU**	$1.30	$1.35	$1.29	$1.08	$0.98
**proportion of PPP Israel/EU**	0.89	0.89	1.03	1.13	1.25
**USD / EUR annual exchange rate**	0.9236	0.90 €	0.9456	1.1308	1.2440
GDP per capita PPS, current EUR (calculated)	20,789.08 €	20,728.09 €	20,695.93 €	19,605.95 €	20,635.14 €

Kosovo					
	2000	2001	2002	2003	2004
GDP/cap in USD	NA	NA	NA	NA	NA
GDP/cap in USD PPP	NA	NA	NA	NA	NA
**purchasing power of 1 USD in Kosovo**	NA	NA	NA	NA	NA
EU GDP/cap in USD.	$16,947.60	$17,199.12	$18,760.98	$22,967.49	$26,307.61
EU GDP/cap in USD PPP	$22,076.90	$23,171.60	$24,186.93	$24,707.28	$25,863.67
**purchasing power of 1 USD in the EU**	$1.30	$1.35	$1.29	$1.08	$0.98
**proportion of PPP Kosovo/EU**	NA	NA	NA	NA	NA
**USD / EUR annual exchange rate**	0.9236	0.90 €	0.9456	1.1308	1.2440
GDP per capita PPS, current EUR (calculated)	NA	NA	NA	NA	NA

Moldova					
	2000	2001	2002	2003	2004
GDP/cap in USD	440.538684	507.4040763	570.7998662	682.319096	897.1784947
GDP/cap in USD PPP	3079.299694	3348.223148	3674.136656	4005.191105	4427.990458
**purchasing power of 1 USD in Moldova**	$6.99	$6.60	$6.44	$5.87	$4.94
EU GDP/cap in USD	$16,947.60	$17,199.12	$18,760.98	$22,967.49	$26,307.61
EU GDP/cap in USD PPP	$22,076.90	$23,171.60	$24,186.93	$24,707.28	$25,863.67
**purchasing power of 1 USD in the EU**	$1.30	$1.35	$1.29	$1.08	$0.98
**proportion of PPP Moldova/EU**	5.37	4.90	4.99	5.46	5.02
**USD / EUR annual exchange rate**	0.9236	0.90 €	0.9456	1.1308	1.2440
GDP per capita PPS, current EUR (calculated)	2,559.40 €	2,774.92 €	3,013.86 €	3,292.50 €	3,620.57 €

Montenegro					
	2000	2001	2002	2003	2004
GDP/cap in USD	1627.072633	1909.598702	2106.635068	2789.159065	3380.165121
GDP/cap in USD PPP	6002.605959	6771.370562	7100.324632	7341.204648	7842.347764
**purchasing power of 1 USD in Montenegro**	$3.69	$3.55	$3.37	$2.63	$2.32
EU GDP/cap in USD.	$16,947.60	$17,199.12	$18,760.98	$22,967.49	$26,307.61
EU GDP/cap in USD PPP	$22,076.90	$23,171.60	$24,186.93	$24,707.28	$25,863.67
**purchasing power of 1 USD in the EU**	$1.30	$1.35	$1.29	$1.08	$0.98
**proportion of PPP Montenegro/EU**	2.83	2.63	2.61	2.45	2.36
**USD / EUR annual exchange rate**	0.9236	0.90 €	0.9456	1.1308	1.2440
GDP per capita PPS, current EUR (calculated)	4,989.14 €	5,611.93 €	5,824.33 €	6,034.90 €	6,412.35 €

Russian Federation					
	2000	2001	2002	2003	2004
GDP/cap in USD	1771.594116	2100.352539	2377.529541	2975.125244	4102.364746
GDP/cap in USD PPP	6825.391602	7361.36377	8037.474609	9254.537109	10226.7666
**purchasing power of 1 USD in Russia**	$3.85	$3.50	$3.38	$3.11	$2.49
EU GDP/cap in USD	$16,947.60	$17,199.12	$18,760.98	$22,967.49	$26,307.61
EU GDP/cap in USD PPP	$22,076.90	$23,171.60	$24,186.93	$24,707.28	$25,863.67

**purchasing power of 1 USD in the EU**	$1.30	$1.35	$1.29	$1.08	$0.98
**proportion of PPP Russia/EU**	2.96	2.60	2.62	2.89	2.54
**USD / EUR annual exchange rate**	0.9236	0.90 €	0.9456	1.1308	1.2440
GDP per capita PPS, current EUR (calculated)	5,673.01 €	6,100.91 €	6,593.06 €	7,607.77 €	8,361.98 €

Ukraine					
	2000	2001	2002	2003	2004
GDP/cap in USD	658.3486328	807.8019409	911.9111328	1087.785645	1416.60376
GDP/cap in USD PPP	4260.276855	4789.681641	5172.960449	5824.124512	6736.79541
**purchasing power of 1 USD in Ukraine**	$6.47	$5.93	$5.67	$5.35	$4.76
EU GDP/cap in USD	$16,947.60	$17,199.12	$18,760.98	$22,967.49	$26,307.61
EU GDP/cap in USD PPP	$22,076.90	$23,171.60	$24,186.93	$24,707.28	$25,863.67
**purchasing power of 1 USD in the EU**	$1.30	$1.35	$1.29	$1.08	$0.98
**proportion of PPP Ukraine/EU**	4.97	4.40	4.40	4.98	4.84
**USD / EUR annual exchange rate**	0.9236	0.90 €	0.9456	1.1308	1.2440
GDP per capita PPS, current EUR (calculated)	3,540.98 €	3,969.56 €	4,243.33 €	4,787.77 €	5,508.38 €

Statistical analysis script for the Frailty Index analyses

```
# Suppl material
# SHARE FRAILTY INDEX ANALYSES
#  0. Housekeeping
###############################
library(foreign)
library(dplyr)
library(lubridate)
library(stringr)
library(tidyr)
library(ggplot2)
library(ggthemes)
library(naniar)
library(PropCIs)
library(lme4)
library(broom.mixed)
library(mice)
library(tibble)

setwd("C:/Users/dhw5d8/OneDrive - Pécsi Tudományegyetem/Dokumentumok/SHARE")
# set your working directory where the SHARE data tables are saved (downloaded as
Stata files)

###############################

#  1. Data load
###############################
# Wave 1
W1_cv_r <- read.dta("sharew1_rel8-0-0_cv_r.dta", missing.type = T) %>%
as.data.frame() %>% select(mergeid, country, gender, yrbirth, mobirth, age2004,
age_int, int_year, int_month)
W1_gv_health <- read.dta("sharew1_rel8-0-0_gv_health.dta", missing.type = T) %>%
as.data.frame() %>% select(mergeid, bmi, bmi2, maxgrip, orienti, sphus)
W1_ph <- read.dta("sharew1_rel8-0-0_ph.dta", missing.type = T) %>% as.data.frame()
%>% select(mergeid, ph012_, ph013_, ph004_, ph006d1,  ph006d2, ph006d4, ph006d5,
ph006d6, ph006d8, ph006d9, ph006d10, ph006d14, ph010d3, ph010d7, ph010d8, ph010d9,
ph048d1, ph048d3, ph048d5, ph048d7, ph048d9, ph049d1, ph049d2, ph049d3, ph049d4,
ph049d5, ph049d6, ph049d8,ph049d9, ph049d11, ph049d12, ph049d13, ph011d11,
ph011d12)
W1_br <- read.dta("sharew1_rel8-0-0_br.dta", missing.type = T) %>% as.data.frame()
%>% select(mergeid, br015_, br016_)
W1_mh <- read.dta("sharew1_rel8-0-0_mh.dta", missing.type = T) %>% as.data.frame()
%>% select(mergeid, mh002_, mh003_, mh011_, mh012_, mh013_, mh016_)
W1_cf <- read.dta("sharew1_rel8-0-0_cf.dta", missing.type = T) %>% as.data.frame()
%>% select(mergeid, cf003_, cf004_, cf005_, cf006_)
W1 <- W1_cv_r %>% left_join(W1_gv_health) %>% left_join(W1_ph) %>%
left_join(W1_br) %>% left_join(W1_mh) %>% left_join(W1_cf) %>% filter(int_year !=
-9)  # Interviews with int_year = -9 were not conducted, these are not valid cases
and are removed
rm(W1_br, W1_cv_r, W1_gv_health, W1_mh, W1_ph, W1_cf)
# define age
W1$mobirth_num <- match(W1$mobirth, month.name); table(W1$mobirth_num, W1$mobirth,
useNA = "always")
W1$int_month_num <- match(W1$int_month, month.name); table(W1$int_month_num,
W1$int_month, useNA = "always")
W1$Age <- ifelse(W1$age_int>0, W1$age_int, ifelse(
  W1$age2004>0, W1$age2004, ifelse(
    (is.na(W1$int_month_num)|is.na(W1$mobirth_num))& W1$yrbirth>0, 2004-
W1$yrbirth, ifelse(
```

```
      W1$mobirth_num <= W1$int_month_num & W1$yrbirth>0, 2004-W1$yrbirth, ifelse(
        W1$mobirth_num > W1$int_month_num & W1$yrbirth>0, 2004 - W1$yrbirth - 1,
NA)))))
summary(W1$Age)
W1_considered <-  length(W1$mergeid)
W1_age_unknown <- length(W1$mergeid[is.na(W1$Age)])
W1_age_below50 <- length(W1$mergeid[is.na(W1$Age) == F & W1$Age < 50])
W1 <- W1 %>% filter(W1$Age >= 50 & !is.na(W1$Age))
W1_eligible <- length(W1$mergeid)
W1$Agebands <- paste0(floor(W1$Age/5)*5, " to ", floor(W1$Age/5)*5+4);
W1$Agebands[W1$Age >= 90] <- "90 or higher"
W1$Agebands <- W1$Agebands %>% factor(levels= sort(unique(W1$Agebands)));
table(W1$Agebands, useNA = "always")
W1$Wave <- "W1"
W1 <- W1 %>% data.frame() %>% select(-age2004)

# Wave 2
W2_cv_r <- read.dta("sharew2_rel8-0-0_cv_r.dta", missing.type = T) %>%
as.data.frame() %>% select(mergeid, country, gender, yrbirth, mobirth, age2007,
age_int, int_year, int_month)
W2_gv_health <- read.dta("sharew2_rel8-0-0_gv_health.dta", missing.type = T) %>%
as.data.frame() %>% select(mergeid, bmi, bmi2, maxgrip, orienti, sphus)
W2_ph <- read.dta("sharew2_rel8-0-0_ph.dta", missing.type = T) %>% as.data.frame()
%>% select(mergeid, ph012_, ph013_, ph004_, ph006d1,  ph006d2, ph006d4, ph006d5,
ph006d6, ph006d8, ph006d9, ph006d10, ph006d14, ph010d3, ph010d7, ph010d8, ph010d9,
ph048d1, ph048d3, ph048d5, ph048d7, ph048d9, ph049d1, ph049d2, ph049d3, ph049d4,
ph049d5, ph049d6, ph049d8,ph049d9, ph049d11, ph049d12, ph049d13, ph011d11,
ph011d12)
W2_br <- read.dta("sharew2_rel8-0-0_br.dta", missing.type = T) %>% as.data.frame()
%>% select(mergeid, br015_, br016_)
W2_mh <- read.dta("sharew2_rel8-0-0_mh.dta", missing.type = T) %>% as.data.frame()
%>% select(mergeid, mh002_, mh003_, mh011_, mh012_, mh013_, mh016_)
W2_cf <- read.dta("sharew2_rel8-0-0_cf.dta", missing.type = T) %>% as.data.frame()
%>% select(mergeid, cf003_, cf004_, cf005_, cf006_)
W2 <- W2_cv_r %>% left_join(W2_gv_health) %>% left_join(W2_ph) %>%
left_join(W2_br) %>% left_join(W2_cf) %>% left_join(W2_mh) %>% filter(int_year !=
-9)  # Interviews with int_year = -9 were not conducted, these are not valid cases
and are removed
rm(W2_br, W2_cv_r, W2_gv_health, W2_mh, W2_ph, W2_cf)
# define age
W2$mobirth_num <- match(W2$mobirth, month.name); # table(W2$mobirth_num,
W2$mobirth, useNA = "always")
W2$int_month_num <- match(W2$int_month, month.name); # table(W2$int_month_num,
W2$int_month, useNA = "always")
W2$Age <- ifelse(W2$age_int>0, W2$age_int, ifelse(
  W2$age2007>0, W2$age2007, ifelse(
    (is.na(W2$int_month_num)|is.na(W2$mobirth_num))& W2$yrbirth>0, 2007-
W2$yrbirth, ifelse(
      W2$mobirth_num <= W2$int_month_num & W2$yrbirth>0, 2007-W2$yrbirth, ifelse(
        W2$mobirth_num > W2$int_month_num & W2$yrbirth>0, 2007 - W2$yrbirth - 1,
NA)))))
summary(W2$Age)
W2_considered <-  length(W2$mergeid)
W2_age_unknown <- length(W2$mergeid[is.na(W2$Age)])
W2_age_below50 <- length(W2$mergeid[is.na(W2$Age) == F & W2$Age < 50])
W2 <- W2 %>% filter(W2$Age >= 50 & !is.na(W2$Age))
W2_eligible <- length(W2$mergeid)
W2$Agebands <- paste0(floor(W2$Age/5)*5, " to ", floor(W2$Age/5)*5+4);
W2$Agebands[W2$Age >= 90] <- "90 or higher"
```

```
W2$Agebands <- W2$Agebands %>% factor(levels= sort(unique(W2$Agebands)));
table(W2$Agebands, useNA = "always")
W2$Wave <- "W2"
W2 <- W2 %>% data.frame() %>% select(names(W1))

# Wave 3
W3 <- read.dta("sharew3_rel8-0-0_cv_r.dta", missing.type = T) %>% as.data.frame()
%>% select(mergeid, country)
# W3_gv_health <- read.dta("sharew3_rel8-0-0_gv_health.dta", missing.type = T) %>%
as.data.frame() %>% select(mergeid, bmi, bmi2, maxgrip, orienti, sphus)
# W3_ph <- read.dta("sharew3_rel8-0-0_ph.dta", missing.type = T) %>%
as.data.frame() %>% select(mergeid, ph012_, ph013_, ph004_, ph006d1,  ph006d2,
ph006d4, ph006d5, ph006d6, ph006d8, ph006d9, ph006d10, ph006d14, ph010d3, ph010d7,
ph010d8, ph010d9, ph048d1, ph048d3, ph048d5, ph048d7, ph048d9, ph049d1, ph049d2,
ph049d3, ph049d4, ph049d5, ph049d6, ph049d8,ph049d9, ph049d11, ph049d12, ph049d13,
ph011d11, ph011d12)
# W3_br <- read.dta("sharew3_rel8-0-0_br.dta", missing.type = T) %>%
as.data.frame() %>% select(mergeid, br015_, br016_)
# W3_mh <- read.dta("sharew3_rel8-0-0_mh.dta", missing.type = T) %>%
as.data.frame() %>% select(mergeid, mh002_, mh003_, mh011_, mh012_, mh013_,
mh016_)
# # no relevant data is available. Hence, Wave 3 is skipped from our analysis.
W3_considered <- length(W3$mergeid)

# Wave 4
W4_cv_r <- read.dta("sharew4_rel8-0-0_cv_r.dta", missing.type = T) %>%
as.data.frame() %>% select(mergeid, country, gender, yrbirth, mobirth, age2011,
age_int, int_year, int_month)
W4_gv_health <- read.dta("sharew4_rel8-0-0_gv_health.dta", missing.type = T) %>%
as.data.frame() %>% select(mergeid, bmi, bmi2, maxgrip, orienti, sphus)
W4_ph <- read.dta("sharew4_rel8-0-0_ph.dta", missing.type = T) %>% as.data.frame()
%>% select(mergeid, ph012_, ph013_, ph004_, ph006d1,  ph006d2, ph006d4, ph006d5,
ph006d6, ph006d8, ph006d9, ph006d10, ph006d14, ph010d3, ph010d7, ph010d8, ph010d9,
ph048d1, ph048d3, ph048d5, ph048d7, ph048d9, ph049d1, ph049d2, ph049d3, ph049d4,
ph049d5, ph049d6, ph049d8,ph049d9, ph049d11, ph049d12, ph049d13, ph011d11,
ph011d12)
W4_br <- read.dta("sharew4_rel8-0-0_br.dta", missing.type = T) %>% as.data.frame()
%>% select(mergeid, br015_, br016_)
W4_mh <- read.dta("sharew4_rel8-0-0_mh.dta", missing.type = T) %>% as.data.frame()
%>% select(mergeid, mh002_, mh003_, mh011_, mh012_, mh013_, mh016_)
W4_cf <- read.dta("sharew4_rel8-0-0_cf.dta", missing.type = T) %>% as.data.frame()
%>% select(mergeid, cf003_, cf004_, cf005_, cf006_)
W4 <- W4_cv_r %>% left_join(W4_gv_health) %>% left_join(W4_ph) %>%
left_join(W4_br) %>% left_join(W4_mh) %>% left_join(W4_cf) %>% filter(int_year !=
-9)  # Interviews with int_year = -9 were not conducted, these are not valid cases
and are removed
rm(W4_br, W4_cv_r, W4_gv_health, W4_mh, W4_ph, W4_cf)
# define age
W4$mobirth_num <- match(W4$mobirth, month.name)
W4$int_month_num <- match(W4$int_month, month.name)
W4$Age <- ifelse(W4$age_int>0, W4$age_int, ifelse(
  W4$age2011>0, W4$age2011, ifelse(
    (is.na(W4$int_month_num)|is.na(W4$mobirth_num))& W4$yrbirth>0, 2011-
W4$yrbirth, ifelse(
      W4$mobirth_num <= W4$int_month_num & W4$yrbirth>0, 2011-W4$yrbirth, ifelse(
        W4$mobirth_num > W4$int_month_num & W4$yrbirth>0, 2011 - W4$yrbirth - 1,
NA)))))
summary(W4$Age)
W4_considered <- length(W4$mergeid)
```

```
W4_age_unknown <- length(W4$mergeid[is.na(W4$Age)])
W4_age_below50 <- length(W4$mergeid[is.na(W4$Age) == F & W4$Age < 50])
W4 <- W4 %>% filter(W4$Age >= 50 & !is.na(W4$Age))
W4_eligible <- length(W4$mergeid)
W4$Agebands <- paste0(floor(W4$Age/5)*5, " to ", floor(W4$Age/5)*5+4);
W4$Agebands[W4$Age >= 90] <- "90 or higher"
W4$Agebands <- W4$Agebands %>% factor(levels= sort(unique(W4$Agebands)))
W4$Wave <- "W4"
W4 <- W4 %>% data.frame() %>% select(names(W1))

######################################  Wave 5

W5_cv_r <- read.dta("sharew5_rel8-0-0_cv_r.dta", missing.type = T) %>%
as.data.frame() %>% select(mergeid, country, gender, yrbirth, mobirth, age2013,
age_int, int_year, int_month)
W5_gv_health <- read.dta("sharew5_rel8-0-0_gv_health.dta", missing.type = T) %>%
as.data.frame() %>% select(mergeid, bmi, bmi2, maxgrip, orienti, sphus)
W5_br <- read.dta("sharew5_rel8-0-0_br.dta", missing.type = T) %>% as.data.frame()
%>% select(mergeid, br015_, br016_)
W5_mh <- read.dta("sharew5_rel8-0-0_mh.dta", missing.type = T) %>% as.data.frame()
%>% select(mergeid, mh002_, mh003_, mh011_, mh012_, mh013_, mh016_)
W5_cf <- read.dta("sharew5_rel8-0-0_cf.dta", missing.type = T) %>% as.data.frame()
%>% select(mergeid, cf003_, cf004_, cf005_, cf006_)

# from wave 5, some elements were deprecated or changes in the ph table. See the
adjustments below:
# ph006d8 is deprecated. Instead, new variables are: ph006d19     Doctor told you
had: rheumatoid arthritis; and ph006d20 Doctor told you had: osteoarthritis/other
rheumatism
# ph006d9 (Doctor told you had: osteoporosis) is deprecated. Instead, new
variables could be ph011d11 & ph011d12: 11. Drugs for osteoporosis, hormonal 12.
Drugs for osteoporosis, other than hormonal.
# check in waves 1-4 whether these parameters are showing the same as ph006d9:
##Check_w1 <- read.dta("sharew1_rel8-0-0_ph.dta", missing.type = T) %>%
as.data.frame() %>% select(mergeid, ph006d9, ph011d11, ph011d12) %>%
mutate(ph006d9_replaced = (ph011d11 == "Selected" |ph011d12 == "Selected"))
##table(Check_w1$ph006d9); table(Check_w1$ph006d9_replaced, Check_w1$ph006d9);
Check_w2 <- read.dta("sharew2_rel8-0-0_ph.dta", missing.type = T) %>%
as.data.frame() %>% select(mergeid, ph006d9, ph011d11, ph011d12) %>%
mutate(ph006d9_replaced = (ph011d11 == "Selected" |ph011d12 == "Selected"));
table(Check_w2$ph006d9); table(Check_w2$ph006d9_replaced, Check_w2$ph006d9)
## rm(Check_w1, Check_w2)
# This replacement approach is not sufficiently sensitive:about half of patients
who were told to have osteoporosis are not taking osteoporosis drugs. Complete
case analyses: only for W1-W4. Multiple imputation analyses: Imputations will be
built on
# all available factor items + supportive data on age, gender, education, marital
status, self-perceived health; but will not include country and wave (since
missingness is complete for some cpountries and waves)
# Column `ph010d3` doesn't exist
# Please look at card 7. For the past six months at least, have you been bothered
by any of the health conditions on this card? Please tell me the number or
numbers.
# 3. Breathlessness, difficulty breathing
# This item is deprecated from Wave 5, with no related replacement question.
# Column `ph010d7` doesn't exist. ph010d7: Please look at card 7. For the past six
months at least, have you been bothered by any of the health conditions on this
card? Please tell me the number or numbers. 7. Falling down
```

```
# This item is deprecated from Wave 5, with a related replacement question, ph089d1: Please look at card ^SHOWCARD_ID. For the past six months at least, have you been bothered by any of the health conditions on this card? Please tell me the number or numbers. 1. Falling down 2. Fear of falling down 3. Dizziness, faints or blackouts 4. Fatigue 96. None
# ph010d7 replaced by ph089d1
# similarly, ph010d8 Fear of falling down is replaced by ph089d2 Fear of falling down
# similarly, ph010d9 Dizziness, faints or blackouts is replaced by ph089d3 Dizziness, faints or blackouts

W5_ph <- read.dta("sharew5_rel8-0-0_ph.dta", missing.type = T) %>% as.data.frame() %>% select(mergeid, ph012_, ph013_, ph004_, ph006d1,  ph006d2, ph006d4, ph006d5, ph006d6, ph006d19, ph006d20, ph006d10, ph006d14, ph089d1, ph089d2, ph089d3, ph048d1, ph048d3, ph048d5, ph048d7, ph048d9, ph049d1, ph049d2, ph049d3, ph049d4, ph049d5, ph049d6, ph049d8,ph049d9, ph049d11, ph049d12, ph049d13, ph011d11)
W5_ph <- W5_ph %>% mutate(ph006d8 = ifelse(ph006d19 == "Selected"|ph006d20 == "Selected", "Selected", ifelse(ph006d19 == "Not selected" & ph006d20 == "Not selected", "Not selected", NA)),
                          ph006d9 = NA,
                          ph010d3 = NA,
                          ph010d7 = ph089d1,
                          ph010d8 = ph089d2,
                          ph010d9 = ph089d3,
                          ph011d12 = "Not selected") # ph011d11 and ph011d12 has been merged from W5 as ph011d11: hormonl and other drugs for osteoporosis are not asked for separately from W5.
# when both options were available in Waves 1,2,4, mospt patient with a positive response responded positively only one of the merged items. Hence, a positive response to the merged item is calculated as a single (and not a double) positive item from Wave 5 onward.
W5 <- W5_cv_r %>% left_join(W5_gv_health) %>% left_join(W5_ph) %>% left_join(W5_br) %>% left_join(W5_mh) %>% left_join(W5_cf)  %>% filter(int_year != -9)  # Interviews with int_year = -9 were not conducted, these are not valid cases and are removed
rm(W5_br, W5_cv_r, W5_gv_health, W5_mh, W5_ph, W5_cf)
W5$mobirth_num <- match(W5$mobirth, month.name)
W5$int_month_num <- match(W5$int_month, month.name)
W5$Age <- ifelse(W5$age_int>0, W5$age_int, ifelse(
  W5$age2013>0, W5$age2013, ifelse(
    (is.na(W5$int_month_num)|is.na(W5$mobirth_num))& W5$yrbirth>0, 2013-W5$yrbirth, ifelse(
      W5$mobirth_num <= W5$int_month_num & W5$yrbirth>0, 2013-W5$yrbirth, ifelse(
        W5$mobirth_num > W5$int_month_num & W5$yrbirth>0, 2013 - W5$yrbirth - 1, NA)))))
summary(W5$Age)
W5_considered <- length(W5$mergeid)
W5_age_unknown <- length(W5$mergeid[is.na(W5$Age)])
W5_age_below50 <- length(W5$mergeid[is.na(W5$Age) == F & W5$Age < 50])
W5 <- W5 %>% filter(W5$Age >= 50 & !is.na(W5$Age))
W5_eligible <- length(W5$mergeid)
W5$Agebands <- paste0(floor(W5$Age/5)*5, " to ", floor(W5$Age/5)*5+4);
W5$Agebands[W5$Age >= 90] <- "90 or higher"
W5$Agebands <- W5$Agebands %>% factor(levels= sort(unique(W5$Agebands)))
W5$Wave <- "W5"
W5 <- W5 %>% data.frame() %>% select(names(W1))

######################################  Wave 6
```

```
W6_cv_r <- read.dta("sharew6_rel8-0-0_cv_r.dta", missing.type = T) %>%
as.data.frame() %>% select(mergeid, country, gender, yrbirth, mobirth, age2015,
age_int, int_year, int_month)
W6_gv_health <- read.dta("sharew6_rel8-0-0_gv_health.dta", missing.type = T) %>%
as.data.frame() %>% select(mergeid, bmi, bmi2, maxgrip, orienti, sphus)
W6_br <- read.dta("sharew6_rel8-0-0_br.dta", missing.type = T) %>% as.data.frame()
%>% select(mergeid, br015_, br016_)
W6_mh <- read.dta("sharew6_rel8-0-0_mh.dta", missing.type = T) %>% as.data.frame()
%>% select(mergeid, mh002_, mh003_, mh011_, mh012_, mh013_, mh016_)
W6_cf <- read.dta("sharew6_rel8-0-0_cf.dta", missing.type = T) %>% as.data.frame()
%>% select(mergeid, cf003_, cf004_, cf005_, cf006_)
W6_ph <- read.dta("sharew6_rel8-0-0_ph.dta", missing.type = T) %>% as.data.frame()
%>% select(mergeid, ph012_, ph013_, ph004_, ph006d1,  ph006d2, ph006d4, ph006d5,
ph006d6, ph006d19, ph006d20, ph006d10, ph006d14, ph089d1, ph089d2, ph089d3,
ph048d1, ph048d3, ph048d5, ph048d7, ph048d9, ph049d1, ph049d2, ph049d3, ph049d4,
ph049d5, ph049d6, ph049d8,ph049d9, ph049d11, ph049d12, ph049d13, ph011d11)
W6_ph <- W6_ph %>% mutate(ph006d8 = ifelse(ph006d19 == "Selected"|ph006d20 ==
"Selected", "Selected", ifelse(ph006d19 == "Not selected" & ph006d20 == "Not
selected", "Not selected", NA)),
                          ph006d9 = NA,
                          ph010d3 = NA,
                          ph010d7 = ph089d1,
                          ph010d8 = ph089d2,
                          ph010d9 = ph089d3,
                          ph011d12 = "Not selected") # ph011d11 and ph011d12 has
been merged from W5 as ph011d11: hormonl and other drugs for osteoporosis are not
asked for separately from W5.

W6 <- W6_cv_r %>% left_join(W6_gv_health) %>% left_join(W6_ph) %>%
left_join(W6_br) %>% left_join(W6_mh) %>% left_join(W6_cf) %>% filter(int_year !=
-9)  # Interviews with int_year = -9 were not conducted, these are not valid cases
and are removed
rm(W6_br, W6_cv_r, W6_gv_health, W6_mh, W6_ph, W6_cf)
W6$mobirth_num <- match(W6$mobirth, month.name)
W6$int_month_num <- match(W6$int_month, month.name)
W6$Age <- ifelse(W6$age_int>0, W6$age_int, ifelse(
  W6$age2015>0, W6$age2015, ifelse(
    (is.na(W6$int_month_num)|is.na(W6$mobirth_num))& W6$yrbirth>0, 2015-
W6$yrbirth, ifelse(
      W6$mobirth_num <= W6$int_month_num & W6$yrbirth>0, 2015-W6$yrbirth, ifelse(
        W6$mobirth_num > W6$int_month_num & W6$yrbirth>0, 2015 - W6$yrbirth - 1,
NA)))))
summary(W6$Age)
W6_considered <- length(W6$mergeid)
W6_age_unknown <- length(W6$mergeid[is.na(W6$Age)])
W6_age_below50 <- length(W6$mergeid[is.na(W6$Age) == F & W6$Age < 50])
W6 <- W6 %>% filter(W6$Age >= 50 & !is.na(W6$Age))
W6_eligible <- length(W6$mergeid)
W6$Agebands <- paste0(floor(W6$Age/5)*5, " to ", floor(W6$Age/5)*5+4);
W6$Agebands[W6$Age >= 90] <- "90 or higher"
W6$Agebands <- W6$Agebands %>% factor(levels= sort(unique(W6$Agebands)))
W6$Wave <- "W6"
W6 <- W6 %>% data.frame() %>% select(names(W1))



######################################  Wave 7
```

```
W7_cv_r <- read.dta("sharew7_rel8-0-0_cv_r.dta", missing.type = T) %>%
as.data.frame() %>% select(mergeid, country, gender, yrbirth, mobirth, age2017,
age_int, int_year, int_month)
W7_gv_health <- read.dta("sharew7_rel8-0-0_gv_health.dta", missing.type = T) %>%
as.data.frame() %>% select(mergeid, bmi, bmi2, maxgrip, orienti, sphus)
W7_br <- read.dta("sharew7_rel8-0-0_br.dta", missing.type = T) %>% as.data.frame()
%>% select(mergeid, br015_, br016_)
W7_mh <- read.dta("sharew7_rel8-0-0_mh.dta", missing.type = T) %>% as.data.frame()
%>% select(mergeid, mh002_, mh003_, mh011_, mh012_, mh013_, mh016_)
W7_cf <- read.dta("sharew7_rel8-0-0_cf.dta", missing.type = T) %>% as.data.frame()
%>% select(mergeid, cf003_, cf004_, cf005_, cf006_)
W7_ph <- read.dta("sharew7_rel8-0-0_ph.dta", missing.type = T) %>% as.data.frame()
%>% select(mergeid, ph012_, ph013_, ph004_, ph006d1,  ph006d2, ph006d4, ph006d5,
ph006d6, ph006d19, ph006d20, ph006d10, ph006d14, ph089d1, ph089d2, ph089d3,
ph048d1, ph048d3, ph048d5, ph048d7, ph048d9, ph049d1, ph049d2, ph049d3, ph049d4,
ph049d5, ph049d6, ph049d8,ph049d9, ph049d11, ph049d12, ph049d13, ph011d11)
W7_ph <- W7_ph %>% mutate(ph006d8 = ifelse(ph006d19 == "Selected"|ph006d20 ==
"Selected", "Selected", ifelse(ph006d19 == "Not selected" & ph006d20 == "Not
selected", "Not selected", NA)),
                          ph006d9 = NA,
                          ph010d3 = NA,
                          ph010d7 = ph089d1,
                          ph010d8 = ph089d2,
                          ph010d9 = ph089d3,
                          ph011d12 = "Not selected") # ph011d11 and ph011d12 has
been merged from W5 as ph011d11: hormonl and other drugs for osteoporosis are not
asked for separately from W5.

W7 <- W7_cv_r %>% left_join(W7_gv_health) %>% left_join(W7_ph) %>%
left_join(W7_br) %>% left_join(W7_mh) %>% left_join(W7_cf) %>% filter(int_year !=
-9)  # Interviews with int_year = -9 were not conducted, these are not valid cases
and are removed
rm(W7_br, W7_cv_r, W7_gv_health, W7_mh, W7_ph, W7_cf)
W7$mobirth_num <- match(W7$mobirth, month.name)
W7$int_month_num <- match(W7$int_month, month.name)
W7$Age <- ifelse(W7$age_int>0, W7$age_int, ifelse(
  W7$age2017>0, W7$age2017, ifelse(
    (is.na(W7$int_month_num)|is.na(W7$mobirth_num))& W7$yrbirth>0, 2017-
W7$yrbirth, ifelse(
      W7$mobirth_num <= W7$int_month_num & W7$yrbirth>0, 2017-W7$yrbirth, ifelse(
        W7$mobirth_num > W7$int_month_num & W7$yrbirth>0, 2017 - W7$yrbirth - 1,
NA)))))
summary(W7$Age)
W7_considered <- length(W7$mergeid)
W7_age_unknown <- length(W7$mergeid[is.na(W7$Age)])
W7_age_below50 <- length(W7$mergeid[is.na(W7$Age) == F & W7$Age < 50])
W7 <- W7 %>% filter(W7$Age >= 50 & !is.na(W7$Age))
W7_eligible <- length(W7$mergeid)
W7$Agebands <- paste0(floor(W7$Age/5)*5, " to ", floor(W7$Age/5)*5+4);
W7$Agebands[W7$Age >= 90] <- "90 or higher"
W7$Agebands <- W7$Agebands %>% factor(levels= sort(unique(W7$Agebands)))
W7$Wave <- "W7"
W7 <- W7 %>% data.frame() %>% select(names(W1))

######################################  Wave 8

W8_cv_r <- read.dta("sharew8_rel8-0-0_cv_r.dta", missing.type = T) %>%
as.data.frame() %>% select(mergeid, country, gender, yrbirth, mobirth, age2020,
age_int, int_year, int_month)
```

```
W8_gv_health <- read.dta("sharew8_rel8-0-0_gv_health.dta", missing.type = T) %>%
as.data.frame() %>% select(mergeid, bmi, bmi2, maxgrip, orienti, sphus)
W8_br <- read.dta("sharew8_rel8-0-0_br.dta", missing.type = T) %>% as.data.frame()
%>% select(mergeid, br015_, br016_)
W8_mh <- read.dta("sharew8_rel8-0-0_mh.dta", missing.type = T) %>% as.data.frame()
%>% select(mergeid, mh002_, mh003_, mh011_, mh012_, mh013_, mh016_)
W8_cf <- read.dta("sharew8_rel8-0-0_cf.dta", missing.type = T) %>% as.data.frame()
%>% select(mergeid, cf003_, cf004_, cf005_, cf006_)
W8_ph <- read.dta("sharew8_rel8-0-0_ph.dta", missing.type = T) %>% as.data.frame()
%>% select(mergeid, ph012_, ph013_, ph004_, ph006d1,  ph006d2, ph006d4, ph006d5,
ph006d6, ph006d19, ph006d20, ph006d10, ph006d14, ph089d1, ph089d2, ph089d3,
ph048d1, ph048d3, ph048d5, ph048d7, ph048d9, ph049d1, ph049d2, ph049d3, ph049d4,
ph049d5, ph049d6, ph049d8,ph049d9, ph049d11, ph049d12, ph049d13, ph011d11)
W8_ph <- W8_ph %>% mutate(ph006d8 = ifelse(ph006d19 == "Selected"|ph006d20 ==
"Selected", "Selected", ifelse(ph006d19 == "Not selected" & ph006d20 == "Not
selected", "Not selected", NA)),
                          ph006d9 = NA,
                          ph010d3 = NA,
                          ph010d7 = ph089d1,
                          ph010d8 = ph089d2,
                          ph010d9 = ph089d3,
                          ph011d12 = "Not selected") # ph011d11 and ph011d12 has
been merged from W5 as ph011d11: hormonl and other drugs for osteoporosis are not
asked for separately from W5.

W8 <- W8_cv_r %>% left_join(W8_gv_health) %>% left_join(W8_ph) %>%
left_join(W8_br) %>% left_join(W8_mh) %>% left_join(W8_cf) %>% filter(int_year !=
-9)  # Interviews with int_year = -9 were not conducted, these are not valid cases
and are removed
rm(W8_br, W8_cv_r, W8_gv_health, W8_mh, W8_ph, W8_cf)
W8$mobirth_num <- match(W8$mobirth, month.name)
W8$int_month_num <- match(W8$int_month, month.name)
W8$Age <- ifelse(W8$age_int>0, W8$age_int, ifelse(
  W8$age2020>0, W8$age2020, ifelse(
    (is.na(W8$int_month_num)|is.na(W8$mobirth_num))& W8$yrbirth>0, 2020-
W8$yrbirth, ifelse(
      W8$mobirth_num <= W8$int_month_num & W8$yrbirth>0, 2020-W8$yrbirth, ifelse(
        W8$mobirth_num > W8$int_month_num & W8$yrbirth>0, 2020 - W8$yrbirth - 1,
NA)))))
summary(W8$Age)
W8_considered <- length(W8$mergeid)
W8_age_unknown <- length(W8$mergeid[is.na(W8$Age)])
W8_age_below50 <- length(W8$mergeid[is.na(W8$Age) == F & W8$Age < 50])
W8 <- W8 %>% filter(W8$Age >= 50 & !is.na(W8$Age))
W8_eligible <- length(W8$mergeid)
W8$Agebands <- paste0(floor(W8$Age/5)*5, " to ", floor(W8$Age/5)*5+4);
W8$Agebands[W8$Age >= 90] <- "90 or higher"
W8$Agebands <- W8$Agebands %>% factor(levels= sort(unique(W8$Agebands)))
W8$Wave <- "W8"
W8 <- W8 %>% data.frame() %>% select(names(W1))

Wall <- rbind(W1, W2, W4, W5, W6, W7, W8); rm(W1, W2, W4, W5, W6, W7, W8)

# GDP per capita PPP data from World Bank. First download the file to working
directory from:
#
https://data.worldbank.org/indicator/NY.GDP.PCAP.PP.KD?locations=EU&type=shaded&vi
ew=map&year=1997
# delete non-European country rows and save the second sheet as csv file.
```

```
GDP <- read.csv("GDP_cap_EUR_2000_2004.csv", stringsAsFactors = F) %>%
  select(country, GDP_cap) %>%
  data.frame()
GDP$country[GDP$country == "Czechia"] <- "Czech Republic"
GDP$country[GDP$country == "Slovak Republic"] <- "Slovakia"
GDP$country[GDP$country == "T\xfcrkiye"] <- "Turkey"
GDP$GDP_cap <- as.numeric(GDP$GDP_cap)
GDP$GDP_cap_k <- GDP$GDP_cap / 1000
GDP <- GDP %>% select(country, GDP_cap_k) %>%
    filter(country %in% c("Iran, Islamic Rep.", "Iraq", "Turkiye", "T\xfcrkiye",
"Turkey", "Liechtenstein") == F)


GDP_PPP <-  read.csv("GDP_cap_EUR_PPP_2000_2004.csv", stringsAsFactors = F) %>%
rename(country = Country) %>% data.frame() # GDP is expressed in EUR PPS:
equivalent customer value to 1 EUR in the EU27
GDP_PPP$country[GDP_PPP$country == "Czechia"] <- "Czech Republic"
GDP_PPP$GDP_cap_PPP <- as.numeric(GDP_PPP$GDP_cap_PPP)
GDP_PPP$GDP_cap_PPP_k <- GDP_PPP$GDP_cap_PPP / 1000
GDP_PPP <- GDP_PPP %>% select(country, GDP_cap_PPP_k) %>%
  filter(country %in% c("Iran, Islamic Rep.", "Iraq", "Turkiye", "T\xfcrkiye",
"Turkey", "Liechtenstein", "United States") == F)


Wall <- Wall %>% mutate(Year = int_year) %>% left_join(GDP) %>% left_join(GDP_PPP)


###############################

#  2. Frailty Index scores in complete cases
###############################
#    as defined by Romero-Ortuno et al. Age Ageing 2012, 41(5):684-689.

Wall$i01 <-  ifelse(Wall$ph049d3 == "Selected", 1, ifelse(Wall$ph049d3 == "Not
selected",0, NA)); table(Wall$ph049d3, Wall$i01, useNA = "always")
Wall$i02 <-  ifelse(Wall$ph049d1 == "Selected", 1, ifelse(Wall$ph049d1 == "Not
selected",0, NA)); table(Wall$ph049d1, Wall$i02, useNA = "always")
Wall$i03 <-  ifelse(Wall$ph048d3 == "Selected", 1, ifelse(Wall$ph048d3 == "Not
selected",0, NA)); table(Wall$ph048d3, Wall$i03, useNA = "always")
Wall$i04 <-  ifelse(Wall$ph049d2 == "Selected", 1, ifelse(Wall$ph049d2 == "Not
selected",0, NA)); table(Wall$ph049d2, Wall$i04, useNA = "always")
Wall$i05 <-  ifelse(Wall$ph049d4 == "Selected", 1, ifelse(Wall$ph049d4 == "Not
selected",0, NA)); table(Wall$ph049d4, Wall$i05, useNA = "always")
Wall$i06 <-  ifelse(Wall$ph048d7 == "Selected", 1, ifelse(Wall$ph048d7 == "Not
selected",0, NA)); table(Wall$ph048d7, Wall$i06, useNA = "always")
Wall$i07 <-  ifelse(Wall$ph049d6 == "Selected", 1, ifelse(Wall$ph049d6 == "Not
selected",0, NA)); table(Wall$ph049d6, Wall$i07, useNA = "always")
Wall$i08 <-  ifelse(Wall$ph048d5 == "Selected", 1, ifelse(Wall$ph048d5 == "Not
selected",0, NA)); table(Wall$ph048d5, Wall$i08, useNA = "always")
Wall$i09 <-  ifelse(Wall$ph048d9 == "Selected", 1, ifelse(Wall$ph048d9 == "Not
selected",0, NA)); table(Wall$ph048d9, Wall$i09, useNA = "always")
Wall$i10 <-  ifelse(Wall$ph049d9 == "Selected", 1, ifelse(Wall$ph049d9 == "Not
selected",0, NA)); table(Wall$ph049d9, Wall$i10, useNA = "always")
Wall$i11 <-  ifelse(Wall$ph049d12 == "Selected", 1, ifelse(Wall$ph049d12 == "Not
selected",0, NA)); table(Wall$ph049d12, Wall$i11, useNA = "always")
Wall$i12 <-  ifelse(Wall$ph049d8 == "Selected", 1, ifelse(Wall$ph049d8 == "Not
selected",0, NA)); table(Wall$ph049d8, Wall$i12, useNA = "always")
```

```
Wall$i13 <-  ifelse(Wall$ph049d11 == "Selected", 1, ifelse(Wall$ph049d11 == "Not
selected",0, NA)); table(Wall$ph049d11, Wall$i13, useNA = "always")
Wall$i14 <-  ifelse(Wall$ph049d13 == "Selected", 1, ifelse(Wall$ph049d13 == "Not
selected",0, NA)); table(Wall$ph049d13, Wall$i14, useNA = "always")
Wall$i15 <-  ifelse(Wall$ph048d1 == "Selected", 1, ifelse(Wall$ph048d1 == "Not
selected",0, NA)); table(Wall$ph048d1, Wall$i15, useNA = "always")
Wall$i16 <-  ifelse(Wall$ph049d5 == "Selected", 1, ifelse(Wall$ph049d5 == "Not
selected",0, NA)); table(Wall$ph049d5, Wall$i16, useNA = "always")
#Phactiv:
Wall$i17 <-  ifelse(Wall$br015_ == "Hardly ever, or never" & Wall$br016_ ==
"Hardly ever, or never", 1, ifelse(
  Wall$br015_ %in% c("Refusal", "Don't know") | Wall$br016_ %in% c("Refusal",
"Don't know"), NA, 0)); table(Wall$br015_, Wall$br016_, Wall$i17, useNA =
"always")
#mh011 & mh012:
Wall$i18 <-  ifelse(
  (is.na(Wall$mh011_)|Wall$mh011_ == "Refusal"|Wall$mh011_ == "Don't know"), NA,
ifelse(
    Wall$mh011_ == "Diminution in desire for food"|(Wall$mh011_ == "Non-specific
or uncodeable response" & !is.na(Wall$mh012_) & Wall$mh012_ == "Less"), 1,ifelse(
      Wall$mh011_ == "Non-specific or uncodeable response" &
(is.na(Wall$mh012_)|Wall$mh012_ == "Refusal"|Wall$mh012_ == "Don't know"), NA,
0)))
table(Wall$mh011_, Wall$mh012_, Wall$i18, useNA = "always")
Wall$i19 <-  (as.numeric(factor(Wall$sphus, levels = c("Excellent", "Very good",
"Good","Fair","Poor")))-1)/4; table(Wall$sphus, Wall$i19, useNA = "always")
# note that spheu was captured only in W1, hence sphus is used instead, with the
same linear scoring function from worst (1) to best (0), using 0.25 steps by
category
Wall$i20 <-  ifelse(Wall$ph004_ == "Yes", 1, ifelse(Wall$ph004_ == "No",0, NA));
table(Wall$ph004_, Wall$i20, useNA = "always")
Wall$i21 <-  ifelse(Wall$mh013_ == "Yes", 1, ifelse(Wall$mh013_ == "No",0, NA));
table(Wall$mh013_, Wall$i21, useNA = "always")
Wall$i22 <-  ifelse(Wall$mh002_ == "Yes", 1, ifelse(Wall$mh002_ == "No",0, NA));
table(Wall$mh002_, Wall$i22, useNA = "always")
Wall$i23 <-  ifelse(Wall$mh016_ == "Fails to mention any enjoyable activity", 1,
ifelse(Wall$mh016_ == "Mentions any enjoyment from activity",0, NA));
table(Wall$mh016_, Wall$i23, useNA = "always")
Wall$i24 <-  ifelse(Wall$mh003_ == "No hopes mentioned", 1, ifelse(Wall$mh003_ ==
"Any hopes mentioned",0, NA)); table(Wall$mh003_, Wall$i24, useNA = "always")
Wall$i25 <-  ifelse(Wall$ph006d2 == "Selected", 1, ifelse(Wall$ph006d2 == "Not
selected",0, NA)); table(Wall$ph006d2, Wall$i25, useNA = "always")
Wall$i26 <-  ifelse(Wall$ph006d1 == "Selected", 1, ifelse(Wall$ph006d1 == "Not
selected",0, NA)); table(Wall$ph006d1, Wall$i26, useNA = "always")
Wall$i27 <-  ifelse(Wall$ph006d4 == "Selected", 1, ifelse(Wall$ph006d4 == "Not
selected",0, NA)); table(Wall$ph006d4, Wall$i27, useNA = "always")
Wall$i28 <-  ifelse(Wall$ph006d10 == "Selected", 1, ifelse(Wall$ph006d10 == "Not
selected",0, NA)); table(Wall$ph006d10, Wall$i28, useNA = "always")
Wall$i29 <-  ifelse(Wall$ph006d5 == "Selected", 1, ifelse(Wall$ph006d5 == "Not
selected",0, NA)); table(Wall$ph006d5, Wall$i29, useNA = "always")
Wall$i30 <-  ifelse(Wall$ph006d8 == "Selected", 1, ifelse(Wall$ph006d8 == "Not
selected",0, NA)); table(Wall$ph006d8, Wall$i30, useNA = "always")
Wall$i31 <-  ifelse(Wall$ph006d6 == "Selected", 1, ifelse(Wall$ph006d6 == "Not
selected",0, NA)); table(Wall$ph006d6, Wall$i31, useNA = "always")
Wall$i32 <-  ifelse(Wall$ph006d9 == "Selected", 1, ifelse(Wall$ph006d9 == "Not
selected",0, NA)); table(Wall$ph006d9, Wall$i32, useNA = "always")
Wall$i32_drugs  <-  ifelse(Wall$ph011d11 == "Selected" |Wall$ph011d12 ==
"Selected", 1, ifelse(Wall$ph011d11 == "Not selected" & Wall$ph011d12 == "Not
selected",0, NA)); table(Wall$i32_drugs, useNA = "always")
```

```
Wall$i33 <-  ifelse(Wall$ph006d14 == "Selected", 1, ifelse(Wall$ph006d14 == "Not
selected",0, NA)); table(Wall$ph006d14, Wall$i33, useNA = "always")
# orienti: Impaired orientation to date, month, year and day of week (i.e. less
than good; 0 bad, 4 good); alternative sources could be: Impaired orientation to
date: cf003_; Impaired orientation to month: cf004_; Impaired orientation to year:
cf005_; Impaired orientation to day of week: cf006_
table(Wall$orienti, useNA = "always") # values are in the 0-4 range, with several
NAs... 4 = good; 0 = bad. Scoring 0 = good, 1 = less than good
Wall$i34 <-  ifelse(is.na(Wall$orienti), NA, ifelse(Wall$orienti == "4", 0, 1));
table(Wall$orienti, Wall$i34, useNA = "always")
Wall$i35 <-  ifelse(is.na(Wall$bmi)|Wall$bmi<0, NA, ifelse(
  (Wall$bmi < 18.5 | Wall$bmi >= 30),1, ifelse(
    (Wall$bmi >=25 & Wall$bmi < 30),0.5,0))); table(Wall$bmi2, Wall$i35, useNA =
"always")
Wall$i36 <-  ifelse(Wall$ph010d3 == "Selected", 1, ifelse(Wall$ph010d3 == "Not
selected",0, NA)); table(Wall$ph010d3, Wall$i36, useNA = "always")
Wall$i37 <-  ifelse(Wall$ph010d7 == "Selected", 1, ifelse(Wall$ph010d7 == "Not
selected",0, NA)); table(Wall$ph010d7, Wall$i37, useNA = "always")
Wall$i38 <-  ifelse(Wall$ph010d8 == "Selected", 1, ifelse(Wall$ph010d8 == "Not
selected",0, NA)); table(Wall$ph010d8, Wall$i38, useNA = "always")
Wall$i39 <-  ifelse(Wall$ph010d9 == "Selected", 1, ifelse(Wall$ph010d9 == "Not
selected",0, NA)); table(Wall$ph010d9, Wall$i39, useNA = "always")
# gripstrength deficit: Man, for BMI ≤ 24, GS ≤ 29; for BMI > 24 and ≤ 28, GS ≤
30; for BMI > 28, GS ≤ 32;; Women: For BMI ≤ 23, GS ≤ 17; For BMI > 23 and ≤ 26,
GS ≤ 17.3; For BMI > 26 and ≤ 29, GS ≤ 18
Wall$i40 <-  ifelse(is.na(Wall$maxgrip)|Wall$maxgrip<0, NA, ifelse(
  Wall$gender == "Male" & Wall$maxgrip > 32, 0, ifelse(
    Wall$gender == "Male" & Wall$maxgrip <=29, 1, ifelse(
      Wall$gender == "Male" & is.na(Wall$bmi), NA, ifelse(
        Wall$gender == "Male" & Wall$bmi<=24 & Wall$maxgrip >29, 0, ifelse(
          Wall$gender == "Male" & Wall$bmi>24 & Wall$bmi <= 28 & Wall$maxgrip
<=30, 1, ifelse(
            Wall$gender == "Male" & Wall$bmi>24 & Wall$bmi <= 28 & Wall$maxgrip
>30, 0, ifelse(
              Wall$gender == "Male" & Wall$bmi>28 & Wall$maxgrip <=32, 1, ifelse(
                Wall$gender == "Female" & Wall$maxgrip > 21, 0, ifelse(
                  Wall$gender == "Female" & Wall$maxgrip <=17, 1, ifelse(
                    Wall$gender == "Female" & is.na(Wall$bmi), NA, ifelse(
                      Wall$gender == "Female" & Wall$bmi<=23 & Wall$maxgrip >17,
0, ifelse(
                        Wall$gender == "Female" & Wall$bmi>23 & Wall$bmi <= 26 &
Wall$maxgrip <=17.3, 1, ifelse(
                          Wall$gender == "Female" & Wall$bmi>23 & Wall$bmi <= 26 &
Wall$maxgrip >17.3, 0, ifelse(
                            Wall$gender == "Female" & Wall$bmi>26 & Wall$bmi <= 29
& Wall$maxgrip <=18, 1, ifelse(
                              Wall$gender == "Female" & Wall$bmi>26 & Wall$bmi <=
29 & Wall$maxgrip >18, 0, ifelse(
                                Wall$gender == "Female" & Wall$bmi>29 &
Wall$maxgrip <=21, 1, "???"))))))))))))))))
table(Wall$i40[Wall$gender == "Male" & Wall$bmi <= 24 & Wall$maxgrip <= 29], useNA
= "always")
table(Wall$i40[Wall$gender == "Male" & Wall$bmi <= 24 & Wall$maxgrip > 29], useNA
= "always")

Wall$MissingComponents <- is.na(Wall$i01) + is.na(Wall$i02) + is.na(Wall$i03) +
is.na(Wall$i04) + is.na(Wall$i05) +
  is.na(Wall$i06) + is.na(Wall$i07) + is.na(Wall$i08) + is.na(Wall$i09) +
is.na(Wall$i10) +
```

```
  is.na(Wall$i11) + is.na(Wall$i12) + is.na(Wall$i13) + is.na(Wall$i14) +
is.na(Wall$i15) +
  is.na(Wall$i16) + is.na(Wall$i17) + is.na(Wall$i18) + is.na(Wall$i19) +
is.na(Wall$i20) +
  is.na(Wall$i21) + is.na(Wall$i22) + is.na(Wall$i23) + is.na(Wall$i24) +
is.na(Wall$i25) +
  is.na(Wall$i26) + is.na(Wall$i27) + is.na(Wall$i28) + is.na(Wall$i29) +
is.na(Wall$i30) +
  is.na(Wall$i31) + is.na(Wall$i32) + is.na(Wall$i33) + is.na(Wall$i34) +
is.na(Wall$i35) +
  is.na(Wall$i36) + is.na(Wall$i37) + is.na(Wall$i38) + is.na(Wall$i39) +
is.na(Wall$i40)

Wall$CompleteCase <- Wall$MissingComponents == 0
table(Wall$CompleteCase, Wall$Wave)
# There are no complete cases from waves 5-8; proportion of complete cases is 80-
90% in waves1-2, and ~10% in Wave 4

###############################

#  3. Missingness patterns
################################
table(Wall$CompleteCase, Wall$Wave)
table(Wall$CompleteCase, Wall$country)
table(Wall$MissingComponents)
vis_miss(Wall %>% select(i01:i40), warn_large_data = F)
# high rate of missingness with clusters. Check by waves:
gg_miss_fct(Wall %>% select(Wave, country, i01:i40), fct = Wave)
# high missingness in WP7 in 9 of 40 frailty index components: drop this wave from
the index analysis
# (dropping a complete wave does not bias the analyses)
# high missingness in WP5 in 3 of 40 frailty index components:
# keep Wave 5 in the analysis (available index components may be sufficient
# to impute the few missing items)
Wall2 <- Wall %>% filter(Wave != "W7")

gg_miss_upset(Wall2 %>% select(i01:i40), nsets = 40, nintersects = 15)
# isolated lack of a few factor components,
# and 12341 cases with complete missingness
table(Wall2$Agebands[Wall2$MissingComponents == 40])
# complete lack of frailty index input was mostly occurring in the highest age
groups
vis_miss(Wall2 %>% select(i01:i40), warn_large_data = F)
# missingness is mostly affecting i32, i36, and i34
# Missingness of i32 and i36 reflects structural changes in the survey from Wave
5.
# Higher proportion of i34 (orienti) missingness at higher ages:
table(Wall2$Agebands, is.na(Wall2$orienti))
# Higher proportion of complete index item missingness at higher ages:
table(Wall2$Agebands, Wall2$MissingComponents == 40)

# Hence, missingness of  Frailty Index items in Wall2 is not at random in the case
of i34.
# Listwise deletion of surveys with missing data (complete case analysis)
# may be biased and is conducted only as sensitivity analysis.
# The primary analysis will include all surveys of the included waves
# using multiple imputation.

###############################
```

```
#  4. Complete case analysis
###############################
Wall_c <- Wall2 %>%
  filter(CompleteCase == T) %>% select(mergeid, Wave, country, Age, Agebands,
gender, int_year, i01:i40, GDP_cap_k, GDP_cap_PPP_k)
summary(Wall_c)

# merge highest agebands
levels(Wall_c$Agebands) <- c(levels(Wall_c$Agebands), "85 or higher")
Wall_c$Agebands[Wall_c$Agebands %in% c("85 to 89", "90 or higher")] <- "85 or
higher"


# factor level and variable type setting
Wall_c$Wave <- factor(Wall_c$Wave)
Wall_c$gender <- as.character(factor(Wall_c$gender, levels = c("Male", "Female")))
Wall_c$int_year <- factor(Wall_c$int_year)
Wall_c$GDP_cap_k <- as.numeric(Wall_c$GDP_cap_k)
Wall_c$i40 <- as.numeric(Wall_c$i40)
Wall_c$GDP_cap_PPP_k <- as.numeric(Wall_c$GDP_cap_PPP_k)
summary(Wall_c)


### calculate the frailty index, using only complete data for assessing frailty -
as it was done in Romero-Ortuno et al. 2012.
# The frailty index in Europeans: association with age and mortality. Age Ageing.
201210.1093/ageing/afs051
Wall_c <- Wall_c %>% mutate(FrailtyIndex =
(i01+i02+i03+i04+i05+i06+i07+i08+i09+i10+

i11+i12+i13+i14+i15+i16+i17+i18+i19+i20+

i21+i22+i23+i24+i25+i26+i27+i28+i29+i30+

i31+i32+i33+i34+i35+i36+i37+i38+i39+i40)/40)
hist(Wall_c$FrailtyIndex)
Wall_c  <- Wall_c %>% mutate(Frail = FrailtyIndex >= 0.25)
# justification for the frailty threshold from Romero Ortuno 2013:
#  "Primarily, the Frailty Index is a continuous variable, but it can be graded to
be equivalent to the phenotypic definition:
#  non-frail (FIx < 0.08), pre-frail (0.08 ≤ FIx < 0.25) and frail (FIx ≥ 0.25)"

table(Wall_c$Agebands, Wall_c$Frail)
ggplot(Wall_c, aes(x=FrailtyIndex, color = country)) + stat_ecdf(geom = "step") +
geom_vline(xintercept = 0.25) + facet_grid(Agebands ~ gender)
#Check the cumulative distribution of the Frailty Index by age bands and sexes by
country.
# The vertical line is the frailty threshold both for females and males.

Summary_c <- Wall_c %>%
  group_by(country, gender, Agebands) %>%
  summarize(
    N_total = n(),
    N_frail =  sum(Frail == TRUE),
    Prop_frail = N_frail / N_total,
    GDP_cap_k = mean(GDP_cap_k),
    GDP_cap_PPP_k = mean(GDP_cap_PPP_k)) %>%
  ungroup()
```

```
Summary_c$Prop_frail_CI_lower <- 99
Summary_c$Prop_frail_CI_upper <- 99
for(i in 1:length(Summary_c$Prop_frail)){
  Summary_c$Prop_frail_CI_lower[i] <-
exactci(Summary_c$N_frail[i],Summary_c$N_total[i], 0.95)[[1]][1]
  Summary_c$Prop_frail_CI_upper[i] <-
exactci(Summary_c$N_frail[i],Summary_c$N_total[i], 0.95)[[1]][2]}

# Visual check of observed data
ggplot(Summary_c) + geom_point(aes(x=GDP_cap_k, y=Prop_frail, color = Agebands,
size = N_total)) +
  facet_grid(gender ~ Agebands) + theme_clean() + theme(axis.text.x =
element_text(angle = 45))

### Mixed model regression to set up predictive function by age, gender and GDP
per capita PPP
# two observed interactions at visual check: gender difference is age dependent;
GDP effect is age dependent.
# fixed factors (predictors) include ageband, gender; and GDP_cap
# random factors to reflect lack of independence in the data: same country:same
mergeid
# random intercept by mergeid: to control for repeated survey in many participants
in different waves
# random intercept by country: to control for other country effects beyond GDP per
capita
# age is not numeric but categorical as non-linear and non loglinear associations
are possible

model_c <- glmer(Frail ~ gender * Agebands + Agebands * GDP_cap_k +
                   (1|country) + (1|country:mergeid), family = binomial(link =
"logit"), data = Wall_c, nAGQ = 0)
summary(model_c) # this is the complete case model
# predictions using this model for extrapolation to other European countries
# visual check of predictions
Summary_c$Predict_prob <- predict(model_c, newdata = Summary_c, re.form = NA, type
= "response")
ggplot(Summary_c) + geom_point(aes(x=GDP_cap_k, y=Prop_frail, color = Agebands,
size = N_total)) +
  geom_line(aes(x=GDP_cap_k, y=Predict_prob), linetype = "dashed", color =
"black", linewidth = 1) +
  facet_grid(gender ~ Agebands) + theme_clean() + theme(axis.text.x =
element_text(angle = 45))

#PPP analysis
model_c_ppp <- glmer(Frail ~ gender * Agebands + Agebands * GDP_cap_PPP_k +
                   (1|country) + (1|country:mergeid), family = binomial(link =
"logit"), data = Wall_c, nAGQ = 0)
summary(model_c_ppp) # this is the complete case model
# predictions using this model for extrapolation to other European countries
# visual check of predictions
Summary_c$Predict_prob_ppp <- predict(model_c_ppp, newdata = Summary_c, re.form =
NA, type = "response")
ggplot(Summary_c) + geom_point(aes(x=GDP_cap_PPP_k, y=Prop_frail, color =
Agebands, size = N_total)) +
  geom_line(aes(x=GDP_cap_PPP_k, y=Predict_prob_ppp), linetype = "dashed", color =
"black", linewidth = 1) +
  facet_grid(gender ~ Agebands) + theme_clean() + theme(axis.text.x =
element_text(angle = 45))
```

```
################################

#  5. Multiple imputation and analysis of the full sample
################################
# Missingness within waves is not at random; a complete case analysis would
systematically undersample patients
# with higher risk for frailty. Data imputations need to take into account
ageband, gender,
# data dimensions of frailty instrument, and some auxiliary variables proposed by
SHARE for hot deck imputations:
# an indicator for people living with a spouse/partner,
# five groups for years of education, and two groups for self-reported good/bad
health.

# Collect data on education years: Waves 2-8, dn041_ How many years have you been
in full time education?
# + Wave 1: isced1997y_r, Respondent: years of education derived from ISCED-97, -7
      Not yet coded (temporary); -2    Refusal; -1  Don't know; 0None; 95
      Still in school; 97 Other
EDU <- rbind(
  read.dta("sharew1_rel8-0-0_gv_isced.dta", missing.type = T) %>% as.data.frame()
%>% select(mergeid, isced1997y_r) %>% mutate(Wave = "W1", isced1997y_r =
ifelse(isced1997y_r<0|isced1997y_r>90, NA, isced1997y_r)) %>% rename(Edu_y =
isced1997y_r),
  read.dta("sharew2_rel8-0-0_dn.dta", missing.type = T) %>% as.data.frame() %>%
select(mergeid, dn041_) %>% mutate(Wave = "W2", dn041_ = ifelse(dn041_<0, NA,
dn041_)) %>% rename(Edu_y = dn041_),
  read.dta("sharew4_rel8-0-0_dn.dta", missing.type = T) %>% as.data.frame() %>%
select(mergeid, dn041_) %>% mutate(Wave = "W4", dn041_ = ifelse(dn041_<0, NA,
dn041_)) %>% rename(Edu_y = dn041_),
  read.dta("sharew5_rel8-0-0_dn.dta", missing.type = T) %>% as.data.frame() %>%
select(mergeid, dn041_) %>% mutate(Wave = "W5", dn041_ = ifelse(dn041_<0, NA,
dn041_)) %>% rename(Edu_y = dn041_),
  read.dta("sharew6_rel8-0-0_dn.dta", missing.type = T) %>% as.data.frame() %>%
select(mergeid, dn041_) %>% mutate(Wave = "W6", dn041_ = ifelse(dn041_<0, NA,
dn041_)) %>% rename(Edu_y = dn041_),
  read.dta("sharew7_rel8-0-0_dn.dta", missing.type = T) %>% as.data.frame() %>%
select(mergeid, dn041_) %>% mutate(Wave = "W7", dn041_ = ifelse(dn041_<0, NA,
dn041_)) %>% rename(Edu_y = dn041_),
  read.dta("sharew8_rel8-0-0_dn.dta", missing.type = T) %>% as.data.frame() %>%
select(mergeid, dn041_) %>% mutate(Wave = "W8", dn041_ = ifelse(dn041_<0, NA,
dn041_)) %>% rename(Edu_y = dn041_))
gg_miss_fct(EDU %>% select(Wave, Edu_y), fct = Wave)
# missing education years data especially in later waves. Imputed using
observations across all waves, assuming no changes in full time education years in
the 50+ years of age, when data is missing:
EDU <- EDU %>% mutate(Edu_y = ifelse(Edu_y <0 |Edu_y>90, NA, Edu_y)) %>%
group_by(mergeid) %>% mutate(Edu_y_i = max(Edu_y, na.rm = T)) %>% ungroup() %>%
select(mergeid, Edu_y_i) %>% distinct()
# generate EDU categories
hist(EDU$Edu_y_i)
table(as.numeric(EDU$Edu_y_i), useNA = "always")
EDU$Edu_y_i[EDU$Edu_y_i < 0] <- NA
table(as.numeric(EDU$Edu_y_i), useNA = "always")
EDU$Edu_cat <- ifelse(is.na(EDU$Edu_y_i), "Missing", ntile(EDU$Edu_y_i, 4)) %>%
factor()
table(EDU$Edu_cat, useNA = "always")
```

```
EDU_table2 <- EDU %>% select(mergeid, Edu_y_i, Edu_cat)
EDU <- EDU %>% select (mergeid, Edu_cat)
EDU$Edu_cat[is.na(EDU$Edu_cat)] <- "Missing"

# Collect data on living with a spouse/partner: dn014_ Marital status: What is
your marital status?
# 1.   Married and living together with spouse; 2.   Registered partnership; 3.
       Married, living separated from spouse; 4.     Never married; 5.   Divorced;
6.     Widowed
SPP <- rbind(
  read.dta("sharew1_rel8-0-0_dn.dta", missing.type = T) %>% as.data.frame() %>%
select(mergeid, dn014_) %>% mutate(Wave = "W1"),
  read.dta("sharew2_rel8-0-0_dn.dta", missing.type = T) %>% as.data.frame() %>%
select(mergeid, dn014_) %>% mutate(Wave = "W2"),
  read.dta("sharew4_rel8-0-0_dn.dta", missing.type = T) %>% as.data.frame() %>%
select(mergeid, dn014_) %>% mutate(Wave = "W4"),
  read.dta("sharew6_rel8-0-0_dn.dta", missing.type = T) %>% as.data.frame() %>%
select(mergeid, dn014_) %>% mutate(Wave = "W6"),
  read.dta("sharew8_rel8-0-0_dn.dta", missing.type = T) %>% as.data.frame() %>%
select(mergeid, dn014_) %>% mutate(Wave = "W8"))
gg_miss_fct(SPP %>% select(Wave, dn014_), fct = Wave)
SPP$MaritalStatus <- ifelse(is.na(SPP$dn014_)|SPP$dn014_ %in% c("Refusal", "Don't
know"), "Missing", ifelse(SPP$dn014_ %in% c("Married and living together with
spouse", "Registered partnership"), "Together", "Alone")) %>% factor(levels =
c("Alone", "Together", "Missing"))
SPP <- SPP %>% select(mergeid, Wave, MaritalStatus)
table(SPP$Wave, SPP$MaritalStatus, useNA = "always")

# Collect data on self-reported good/bad health: ph003_ Would you say your health
is...1. Excellent 2. Very good 3. Good 4. Fair 5. Poor
SRH <- rbind(
  read.dta("sharew1_rel8-0-0_ph.dta", missing.type = T) %>% as.data.frame() %>%
select(mergeid, ph003_) %>% mutate(Wave = "W1"),
  read.dta("sharew2_rel8-0-0_ph.dta", missing.type = T) %>% as.data.frame() %>%
select(mergeid, ph003_) %>% mutate(Wave = "W2"),
  read.dta("sharew4_rel8-0-0_ph.dta", missing.type = T) %>% as.data.frame() %>%
select(mergeid, ph003_) %>% mutate(Wave = "W4"),
  read.dta("sharew6_rel8-0-0_ph.dta", missing.type = T) %>% as.data.frame() %>%
select(mergeid, ph003_) %>% mutate(Wave = "W6"),
  read.dta("sharew8_rel8-0-0_ph.dta", missing.type = T) %>% as.data.frame() %>%
select(mergeid, ph003_) %>% mutate(Wave = "W8"))
gg_miss_fct(SRH %>% select(Wave, ph003_), fct = Wave)
SRH$Health <- ifelse(is.na(SRH$ph003_)|SRH$ph003_ %in% c("Refusal", "Don't know"),
"Missing", ifelse(SRH$ph003_ %in% c("Excellent", "Very good", "Good"), "Good",
"Not good")) %>% factor(levels = c("Good", "Not good", "Missing"))
table(SRH$ph003_, SRH$Health, useNA = "always")
table(SRH$Wave, SRH$Health, useNA = "always")
SRH <- SRH %>% select(mergeid, Wave, Health)

###### combine Wall2 with auxiliary data for missing data imputations
Wall_i <- Wall2 %>%
  select(mergeid, Wave, country, Age, Agebands, gender, int_year, i01:i40,
GDP_cap_k, GDP_cap_PPP_k, MissingComponents, CompleteCase) %>%
  left_join(EDU) %>%
  left_join(SPP) %>%
  left_join(SRH)
Wall_i$Edu_cat[is.na(Wall_i$Edu_cat)] <- "Missing"
Wall_i$MaritalStatus[is.na(Wall_i$MaritalStatus)] <- "Missing"
Wall_i$Health[is.na(Wall_i$Health)] <- "Missing"
```

```
Wall_i$gender <- factor(Wall_i$gender, levels = c("Male", "Female"))
Wall_i$i40 <- as.numeric(Wall_i$i40)
summary(Wall_i)

# adjust parameter types before imputation
Wall_i$i01 <- factor(Wall_i$i01); table(Wall_i$i01, useNA = "always")
Wall_i$i02 <- factor(Wall_i$i02); table(Wall_i$i02, useNA = "always")
Wall_i$i03 <- factor(Wall_i$i03); table(Wall_i$i03, useNA = "always")
Wall_i$i04 <- factor(Wall_i$i04); table(Wall_i$i04, useNA = "always")
Wall_i$i05 <- factor(Wall_i$i05); table(Wall_i$i05, useNA = "always")
Wall_i$i06 <- factor(Wall_i$i06); table(Wall_i$i06, useNA = "always")
Wall_i$i07 <- factor(Wall_i$i07); table(Wall_i$i07, useNA = "always")
Wall_i$i08 <- factor(Wall_i$i08); table(Wall_i$i08, useNA = "always")
Wall_i$i09 <- factor(Wall_i$i09); table(Wall_i$i09, useNA = "always")
Wall_i$i10 <- factor(Wall_i$i10); table(Wall_i$i10, useNA = "always")

Wall_i$i11 <- factor(Wall_i$i11); table(Wall_i$i11, useNA = "always")
Wall_i$i12 <- factor(Wall_i$i12); table(Wall_i$i12, useNA = "always")
Wall_i$i13 <- factor(Wall_i$i13); table(Wall_i$i13, useNA = "always")
Wall_i$i14 <- factor(Wall_i$i14); table(Wall_i$i14, useNA = "always")
Wall_i$i15 <- factor(Wall_i$i15); table(Wall_i$i15, useNA = "always")
Wall_i$i16 <- factor(Wall_i$i16); table(Wall_i$i16, useNA = "always")
Wall_i$i17 <- factor(Wall_i$i17); table(Wall_i$i17, useNA = "always")
Wall_i$i18 <- factor(Wall_i$i18); table(Wall_i$i18, useNA = "always")
Wall_i$i19 <- as.numeric(as.character(Wall_i$i19)); table(Wall_i$i19, useNA =
"always")
Wall_i$i20 <- factor(Wall_i$i20); table(Wall_i$i20, useNA = "always")

Wall_i$i21 <- factor(Wall_i$i21); table(Wall_i$i21, useNA = "always")
Wall_i$i22 <- factor(Wall_i$i22); table(Wall_i$i22, useNA = "always")
Wall_i$i23 <- factor(Wall_i$i23); table(Wall_i$i23, useNA = "always")
Wall_i$i24 <- factor(Wall_i$i24); table(Wall_i$i24, useNA = "always")
Wall_i$i25 <- factor(Wall_i$i25); table(Wall_i$i25, useNA = "always")
Wall_i$i26 <- factor(Wall_i$i26); table(Wall_i$i26, useNA = "always")
Wall_i$i27 <- factor(Wall_i$i27); table(Wall_i$i27, useNA = "always")
Wall_i$i28 <- factor(Wall_i$i28); table(Wall_i$i28, useNA = "always")
Wall_i$i29 <- factor(Wall_i$i29); table(Wall_i$i29, useNA = "always")
Wall_i$i30 <- factor(Wall_i$i30); table(Wall_i$i30, useNA = "always")

Wall_i$i31 <- factor(Wall_i$i31); table(Wall_i$i31, useNA = "always")
Wall_i$i32 <- factor(Wall_i$i32); table(Wall_i$i32, useNA = "always")
Wall_i$i33 <- factor(Wall_i$i33); table(Wall_i$i33, useNA = "always")
Wall_i$i34 <- factor(Wall_i$i34); table(Wall_i$i34, useNA = "always")
Wall_i$i35 <- as.numeric(as.character(Wall_i$i35)); table(Wall_i$i35, useNA =
"always")
Wall_i$i36 <- factor(Wall_i$i36); table(Wall_i$i36, useNA = "always")
Wall_i$i37 <- factor(Wall_i$i37); table(Wall_i$i37, useNA = "always")
Wall_i$i38 <- factor(Wall_i$i38); table(Wall_i$i38, useNA = "always")
Wall_i$i39 <- factor(Wall_i$i39); table(Wall_i$i39, useNA = "always")
Wall_i$i40 <- factor(Wall_i$i40); table(Wall_i$i40, useNA = "always")
Wall_i$i32_drugs <- factor(Wall_i$i32_drugs); table(Wall_i$i32_drugs, useNA =
"always")
summary(Wall_i)

# get prepared for imputations
init = mice(Wall_i, maxit=0)
meth = init$method
predM = init$predictorMatrix
```

```
predM[, c("mergeid", "MissingComponents", "country", "Wave",
"CompleteCase","GDP_cap_k", "GDP_cap_PPP_k", "int_year", "Age")]=0
# remove these variable as predictors

meth[c("mergeid", "Wave", "country", "Edu_cat", "MaritalStatus", "Health",
"MissingComponents", "CompleteCase",
       "i32_drugs", "Age", "Agebands", "gender")]=""
# Columns that need not be imputed have the empty method "".

# Now let specify the methods for imputing the missing values. There are specific
methods for continues, binary and ordinal variables:
meth[c("i19", "i35")]="pmm"
meth[c("i01", "i02", "i03", "i04", "i05", "i06", "i07", "i08", "i09", "i10",
"i11", "i12", "i13",
       "i14", "i15", "i16", "i17", "i18", "i20", "i21", "i22", "i23", "i24",
"i25", "i26", "i27",
       "i28", "i29", "i30", "i31", "i32", "i33", "i34", "i36", "i37", "i38",
"i39", "i40")] = "logreg"

set.seed(2023)
imputed = mice(Wall_i, method=meth, predictorMatrix=predM, m=10)
imp_long <- complete(imputed, action = "long", include = T)

imp_long$FrailtyIndex <- 99
imp_long$i40 <- as.numeric(as.character(imp_long$i40))
imp_long <- imp_long %>% rowwise() %>% mutate(
  FrailtyIndex = sum(as.numeric(as.character(i01)), as.numeric(as.character(i02)),
as.numeric(as.character(i03)), as.numeric(as.character(i04)),
as.numeric(as.character(i05)),
                     as.numeric(as.character(i06)), as.numeric(as.character(i07)),
as.numeric(as.character(i08)), as.numeric(as.character(i09)),
as.numeric(as.character(i10)),
                     as.numeric(as.character(i11)), as.numeric(as.character(i12)),
as.numeric(as.character(i13)), as.numeric(as.character(i14)),
as.numeric(as.character(i15)),
                     as.numeric(as.character(i16)), as.numeric(as.character(i17)),
as.numeric(as.character(i18)), as.numeric(as.character(i19)),
as.numeric(as.character(i20)),
                     as.numeric(as.character(i21)), as.numeric(as.character(i22)),
as.numeric(as.character(i23)), as.numeric(as.character(i24)),
as.numeric(as.character(i25)),
                     as.numeric(as.character(i26)), as.numeric(as.character(i27)),
as.numeric(as.character(i28)), as.numeric(as.character(i29)),
as.numeric(as.character(i30)),
                     as.numeric(as.character(i31)), as.numeric(as.character(i32)),
as.numeric(as.character(i33)), as.numeric(as.character(i34)),
as.numeric(as.character(i35)),
                     as.numeric(as.character(i36)), as.numeric(as.character(i37)),
as.numeric(as.character(i38)), as.numeric(as.character(i39)),
as.numeric(as.character(i40)),
                     na.rm = F)/40) %>% ungroup
hist(imp_long$FrailtyIndex)
table(is.na(imp_long$FrailtyIndex), imp_long$.imp) # the imputation was incomplete

gg_miss_upset(imp_long %>% filter(.imp == 1) %>% select(i01:i40), nsets = 40,
nintersects = 15)
gg_miss_upset(imp_long %>% filter(.imp == 2) %>% select(i01:i40), nsets = 40,
nintersects = 15) # the same
```

```
# incomplete imputations dominated by combined missingness of data on multiple
frailty index components: simplified imputation of the index itself
# incomplete imputations are associated with increasing age: need for further
imputation efforts (do not drop the not imputed rows)
table(imp_long$Agebands, is.na(imp_long$FrailtyIndex), imp_long$.imp)

# select ids with incomplete imputations and complete case ids
imputed_2 <- imp_long %>% select(.imp, .id, mergeid, Wave, country, Agebands,
gender,
                                  Edu_cat, MaritalStatus, Health,
MissingComponents, FrailtyIndex)
imputed_2_completecases <- imputed_2 %>% filter(MissingComponents == 0)
imputed_2_missingFI <- imputed_2 %>% filter(.id %in% imputed_2$.id[imputed_2$.imp
> 0 & is.na(imputed_2$FrailtyIndex)])
imputed2_imputationcomplete <- imputed_2 %>% filter(.id %in%
imputed_2_completecases$.id == F & .id %in% imputed_2_missingFI$.id == F )
table(imputed_2_completecases$.imp)
table(imputed_2_missingFI$.imp)
table(imputed2_imputationcomplete$.imp)
imputed_2_select <- rbind(imputed_2_completecases, imputed_2_missingFI)
table(imputed_2_select$MissingComponents, imputed_2_select$.imp,
is.na(imputed_2_select$FrailtyIndex))
imputed_2_select <- imputed_2_select %>% mutate(old.imp = .imp, old.id = .id, .imp
= NULL, .id = NULL)

# impute the missing data on frailty index
init = mice(imputed_2_select, maxit=0)
meth = init$method
predM = init$predictorMatrix
predM[, c("mergeid", "MissingComponents", "country", "Wave", "FrailtyIndex",
"old.imp", "old.id")]=0
# remove these variable as predictors but these still will be imputed.
meth[c("mergeid", "Wave", "country", "Edu_cat", "MaritalStatus", "Health",
"MissingComponents", "Agebands", "gender", "old.id", "old.imp")]=""
# Columns that need not be imputed have the empty method "".
# Now let specify the methods for imputing the missing values. There are specific
methods for continues, binary and ordinal variables:
meth[c("FrailtyIndex")]="pmm"
set.seed(2023)
imputed_new2 = mice(imputed_2_select, method=meth, predictorMatrix=predM, m=1)
imputed_3 <- complete(imputed_new2, action = "long", include = T)
table(is.na(imputed_3$FrailtyIndex[imputed_3$.imp == 0]))
table(is.na(imputed_3$FrailtyIndex[imputed_3$.imp == 1]))
# imputation is complete for all rows. Select the completed imputation set:
imputed_3 <- imputed_3 %>% filter(.imp == 1) %>% mutate(.id = old.id, .imp =
old.imp, old.id = NULL, old.imp = NULL)

# merge all data for a complete imputed dataset
imputed_all_long <- rbind(imputed_3, imputed2_imputationcomplete) %>%
mutate(Frailty = FrailtyIndex >= 0.25)
table(imputed_all_long$Agebands, imputed_all_long$Frailty, imputed_all_long$.imp,
useNA = "always")
# imputation is complete


# descriptive analyses of the imputed datasets
Wall_i_plus <- Wall_i %>% select(mergeid, Wave, int_year, CompleteCase, GDP_cap_k,
GDP_cap_PPP_k)
Wall_i_plus$GDP_cap_k <- as.numeric(Wall_i_plus$GDP_cap_k)
```

```
Wall_i_plus$GDP_cap_PPP_k <- as.numeric(Wall_i_plus$GDP_cap_PPP_k)

# merge highest agebands
levels(imputed_all_long$Agebands) <- c(levels(imputed_all_long$Agebands), "85 or
higher")
imputed_all_long$Agebands[imputed_all_long$Agebands %in% c("85 to 89", "90 or
higher")] <- "85 or higher"

Wall_i2 <- imputed_all_long %>% mutate(Wave = factor(Wave)) %>%
left_join(Wall_i_plus) %>% mutate(
  int_year = factor(int_year))

summary(Wall_i2)

Summary_i <- Wall_i2 %>% filter(.imp >0) %>% mutate(imp = .imp) %>%
  group_by(country, gender, Agebands) %>%
  summarize(
  N_total = n()/10,
  N_frail = round(sum(Frailty == T)/10,0),
  Prop_frail = N_frail / N_total,
  GDP_cap_k = mean(GDP_cap_k),
  GDP_cap_PPP_k = mean(GDP_cap_PPP_k)) %>%
  ungroup()

Summary_i$Prop_frail_CI_lower <- 99
Summary_i$Prop_frail_CI_upper <- 99
for(i in 1:length(Summary_i$Prop_frail)){
  Summary_i$Prop_frail_CI_lower[i] <-
exactci(Summary_i$N_frail[i],Summary_i$N_total[i], 0.95)[[1]][1]
  Summary_i$Prop_frail_CI_upper[i] <-
exactci(Summary_i$N_frail[i],Summary_i$N_total[i], 0.95)[[1]][2]}

# This is the descriptive analysis results, average of 10 multiple imputations.

Wall_i_modeldata <- Wall_i2 %>% select(.imp, .id, mergeid, Wave, country, gender,
Agebands,
                                       int_year, GDP_cap_k, GDP_cap_PPP_k,
FrailtyIndex, Frailty) %>%
  arrange(.imp, .id) %>% select(.imp, .id, Frailty, mergeid, Wave, country,
gender, Agebands,
                                GDP_cap_k, GDP_cap_PPP_k) %>% mutate(Wave =
factor(Wave), gender = as.character(gender)) %>%
  arrange(.imp, .id)

imputed_mids <- as.mids(Wall_i_modeldata)

# Mixed model regression analysis:
model_i_index <- with(imputed_mids, glmer(Frailty ~ gender * Agebands + Agebands *
GDP_cap_k + (1|country) + (1|country:mergeid), family = binomial(link = "logit"),
nAGQ = 0))
model_i_index_pooledresults <- summary(pool(model_i_index, dfcom =
model_i_index$analyses[[1]]@devcomp$dims[4]), rule = "rubin1987")
# prediction model
model_i_index_predict <- model_i_index$analyses[[1]]
model_i_index_predict@beta <- model_i_index_pooledresults$estimate
# visual check of predictions
Summary_i$Predict_prob <- predict(model_i_index_predict, newdata = Summary_i,
re.form = NA, type = "response")
```

```
ggplot(Summary_i) + geom_point(aes(x=GDP_cap_k, y=Prop_frail, color = Agebands,
size = N_total)) +
  geom_line(aes(x=GDP_cap_k, y=Predict_prob), linetype = "dashed", color =
"black", size = 1) +
  facet_grid(gender ~ Agebands) + theme_clean() + theme(axis.text.x =
element_text(angle = 45))


# PPP analysis: mixed model regression analysis using PPP GDP data (purchasing
power parity)
model_i_ppp_index <- with(imputed_mids, glmer(Frailty ~ gender * Agebands +
Agebands * GDP_cap_PPP_k +
                                            (1|country) + (1|country:mergeid),
family = binomial(link = "logit"), nAGQ = 0))
model_i_ppp_index_pooledresults <- summary(pool(model_i_ppp_index, dfcom =
model_i_ppp_index$analyses[[1]]@devcomp$dims[4]), rule = "rubin1987")
# prediction model
model_i_ppp_index_predict <- model_i_ppp_index$analyses[[1]]
model_i_ppp_index_predict@beta <- model_i_ppp_index_pooledresults$estimate
# visual check of predictions
Summary_i$Predict_prob_ppp <- predict(model_i_ppp_index_predict, newdata =
Summary_i, re.form = NA, type = "response")
ggplot(Summary_i) + geom_point(aes(x=GDP_cap_PPP_k, y=Prop_frail, color =
Agebands, size = N_total)) +
  geom_line(aes(x=GDP_cap_PPP_k, y=Predict_prob_ppp), linetype = "dashed", color =
"black", size = 1) +
  facet_grid(gender ~ Agebands) + theme_clean() + theme(axis.text.x =
element_text(angle = 45))

# combine completecase and imputed data on a plot

ggplot() +
  geom_point(
    data = Summary_c,
    aes(x=GDP_cap_k, y=Prop_frail, size = N_total),
    shape = 21, color = "black", fill = NA) +
  geom_point(
    data = Summary_i,
    aes(x=GDP_cap_k, y=Prop_frail, size = N_total),
    shape = 21, color = "darkgreen", fill = "darkgreen", alpha = 0.2) +
  geom_line(
    data = Summary_c,
    aes(x=GDP_cap_k, y=Predict_prob),
    color = "black") +
  geom_line(
    data = Summary_i,
    aes(x=GDP_cap_k, y=Predict_prob), color = "darkgreen") +
  facet_grid(gender ~ Agebands) +
  theme_clean() + theme(axis.text.x = element_text(angle = 45))

###############################

#  6. Derive data for the Frailty Atlas
###############################
# The comprehensive list of frailty estimates by country, gender, and age bands
with 95% confidence intervals
# will be provided in tabular format as part of the SHARE Frailty Atlas (online
suppl. materials)
```

```
Country_list <- data.frame(crossing(country = GDP$country, Agebands =
Wall_c$Agebands,
                                   gender = c("Male", "Female"))) %>%
  filter(Agebands %in% c("85 to 89", "90 or higher") == F) %>%
  droplevels()

Atlas_Index_obs_cc <-  Summary_c %>%
  select(country, gender, Agebands, Prop_frail, Prop_frail_CI_lower,
Prop_frail_CI_upper) %>%
  right_join(Country_list) %>% select(country, gender, Agebands, Prop_frail,
                                     Prop_frail_CI_lower, Prop_frail_CI_upper)
%>%
  mutate(datatype = "Observed values", Missingness = "Complete cases",
         Frailty_method = "SHARE Frailty Index")

Atlas_Index_obs_imp <-  Summary_i %>%
  select(country, gender, Agebands, Prop_frail, Prop_frail_CI_lower,
Prop_frail_CI_upper) %>%
  right_join(Country_list) %>% mutate(datatype = "Observed values", Missingness =
"Multiple imputation",
                                     Frailty_method = "SHARE Frailty Index")
%>%select(names(Atlas_Index_obs_cc))

Atlas_Index_predicted_cc_GDP_kEUR <- Country_list %>%
  left_join(GDP %>% select(country, GDP_cap_k)) %>%
  mutate(GDP_cap_k = as.numeric(GDP_cap_k),
         datatype = "Prediction_GDP_kEUR",
         Prop_frail_CI_lower = NA, Prop_frail_CI_upper = NA,
         Missingness = "Complete cases",
         Frailty_method = "SHARE Frailty Index")
Atlas_Index_predicted_cc_GDP_kEUR$Prop_frail <- predict(
  model_c, newdata = Atlas_Index_predicted_cc_GDP_kEUR, re.form = NA, type =
"response")
Atlas_Index_predicted_cc_GDP_kEUR <- Atlas_Index_predicted_cc_GDP_kEUR %>%
select(names(Atlas_Index_obs_cc))

Atlas_Index_predicted_cc_GDP_PPP <- Country_list %>%
  left_join(GDP_PPP %>% select(country, GDP_cap_PPP_k)) %>%
  mutate(GDP_cap_PPP_k = as.numeric(GDP_cap_PPP_k),
         datatype = "Prediction_GDP_PPP",
         Prop_frail_CI_lower = NA, Prop_frail_CI_upper = NA,
         Missingness = "Complete cases",
         Frailty_method = "SHARE Frailty Index")
Atlas_Index_predicted_cc_GDP_PPP$Prop_frail <- predict(
  model_c_ppp, newdata = Atlas_Index_predicted_cc_GDP_PPP, re.form = NA, type =
"response")
Atlas_Index_predicted_cc_GDP_PPP <- Atlas_Index_predicted_cc_GDP_PPP %>%
select(names(Atlas_Index_obs_cc))

Atlas_Index_predicted_imp_GDP_kEUR <- Country_list %>%
  left_join(GDP %>% select(country, GDP_cap_k)) %>%
  mutate(GDP_cap_k = as.numeric(GDP_cap_k),
         datatype = "Prediction_GDP_kEUR",
         Prop_frail_CI_lower = NA, Prop_frail_CI_upper = NA,
         Missingness = "Multiple imputation",
         Frailty_method = "SHARE Frailty Index")
Atlas_Index_predicted_imp_GDP_kEUR$Prop_frail <- predict(
  model_i_index_predict, newdata = Atlas_Index_predicted_imp_GDP_kEUR, re.form =
NA, type = "response")
```

```
Atlas_Index_predicted_imp_GDP_kEUR <- Atlas_Index_predicted_imp_GDP_kEUR %>%
select(names(Atlas_Index_obs_cc))

Atlas_Index_predicted_imp_GDP_PPP <- Country_list %>%
  left_join(GDP_PPP %>% select(country, GDP_cap_PPP_k)) %>%
  mutate(GDP_cap_PPP_k = as.numeric(GDP_cap_PPP_k),
         datatype = "Prediction_GDP_PPP",
         Prop_frail_CI_lower = NA, Prop_frail_CI_upper = NA,
         Missingness = "Multiple imputation",
         Frailty_method = "SHARE Frailty Index")
Atlas_Index_predicted_imp_GDP_PPP$Prop_frail <- predict(
  model_i_ppp_index_predict, newdata = Atlas_Index_predicted_imp_GDP_PPP, re.form
= NA, type = "response")
Atlas_Index_predicted_imp_GDP_PPP <- Atlas_Index_predicted_imp_GDP_PPP %>%
select(names(Atlas_Index_obs_cc))

Atlas_Index <- rbind(Atlas_Index_obs_cc,
                          Atlas_Index_obs_imp,
                          Atlas_Index_predicted_cc_GDP_kEUR,
                          Atlas_Index_predicted_cc_GDP_PPP,
                          Atlas_Index_predicted_imp_GDP_kEUR,
                          Atlas_Index_predicted_imp_GDP_PPP)
write.csv(as.data.frame(Atlas_Index), "Atlas_Index_20230927.csv")

###############################

#  8. Tables and figures for the manuscript
############################

# Figure 1. Flowchart
# Surveys covered in SHARE Waves 1-8
#Considered
W1_considered + W2_considered + W3_considered + W4_considered +
  W5_considered + W6_considered + W7_considered + W8_considered
# Excluded_W3
W3_considered
# Excluded_age_unknown
W1_age_unknown + W2_age_unknown + W4_age_unknown + W5_age_unknown +
  W6_age_unknown + W7_age_unknown + W8_age_unknown
# Excluded_age_below50
W1_age_below50 + W2_age_below50 + W4_age_below50 + W5_age_below50 +
  W6_age_below50 + W7_age_below50 + W8_age_below50
# Eligible
W1_eligible + W2_eligible + W4_eligible + W5_eligible + W6_eligible + W7_eligible
+ W8_eligible
# Descriptive analysis, index
# exclude W7
table(Wall$Wave == "W7")
# analyses, complete case / multiple imputation
length(Wall_c$mergeid)
length(Wall_i$mergeid)


# Supplementary Table 1. Data missingness on frailty index components
#######
# by agebands
Wall2 %>%
  group_by(Agebands) %>%   summarize(Total = n(), Complete = sum(CompleteCase),
```

```
    Incomplete = n() - sum(CompleteCase), Total_percent = round(Total /
length(Wall2$mergeid), 4),
    Complete_agedistr = round(Complete / length(Wall2$mergeid[Wall2$CompleteCase
== T]), 4), Prop_incomplete = round(Incomplete / Total , 4)) %>%
  ungroup() %>% as.data.frame() %>% print() %>% filter(!is.na(Agebands)) %>%
select(Agebands, Complete, Incomplete) %>%
  column_to_rownames("Agebands") %>% chisq.test()
# by gender
Wall2 %>%
  group_by(gender) %>%   summarize(Total = n(), Complete = sum(CompleteCase),
                                     Incomplete = n() - sum(CompleteCase),
Total_percent = round(Total / length(Wall2$mergeid), 4),
                                     Prop_incomplete = round(Incomplete / Total ,
4)) %>%
  ungroup() %>% as.data.frame() %>% print() %>% filter(!is.na(gender)) %>%
select(gender, Complete, Incomplete) %>%
  column_to_rownames("gender") %>% chisq.test()
# by index components
Wall2 %>% group_by(i01) %>%
  summarize(Total = n(), Complete = sum(CompleteCase), Incomplete = n() -
sum(CompleteCase), Total_percent = round(Total / length(Wall2$mergeid), 4),
Prop_incomplete = round(Incomplete / Total , 4)) %>%
  ungroup() %>% as.data.frame() %>% print() %>% filter(!is.na(i01)) %>%
  select(i01, Complete, Incomplete) %>%
  column_to_rownames("i01") %>% chisq.test()
Wall2 %>% group_by(i02) %>%
  summarize(Total = n(), Complete = sum(CompleteCase), Incomplete = n() -
sum(CompleteCase), Total_percent = round(Total / length(Wall2$mergeid), 4),
Prop_incomplete = round(Incomplete / Total , 4)) %>%
  ungroup() %>% as.data.frame() %>% print() %>% filter(!is.na(i02)) %>%
  select(i02, Complete, Incomplete) %>%
  column_to_rownames("i02") %>% chisq.test()
Wall2 %>% group_by(i03) %>%
  summarize(Total = n(), Complete = sum(CompleteCase), Incomplete = n() -
sum(CompleteCase), Total_percent = round(Total / length(Wall2$mergeid), 4),
Prop_incomplete = round(Incomplete / Total , 4)) %>%
  ungroup() %>% as.data.frame() %>% print() %>% filter(!is.na(i03)) %>%
  select(i03, Complete, Incomplete) %>%
  column_to_rownames("i03") %>% chisq.test()
Wall2 %>% group_by(i04) %>%
  summarize(Total = n(), Complete = sum(CompleteCase), Incomplete = n() -
sum(CompleteCase), Total_percent = round(Total / length(Wall2$mergeid), 4),
Prop_incomplete = round(Incomplete / Total , 4)) %>%
  ungroup() %>% as.data.frame() %>% print() %>% filter(!is.na(i04)) %>%
  select(i04, Complete, Incomplete) %>%
  column_to_rownames("i04") %>% chisq.test()
Wall2 %>% group_by(i05) %>%
  summarize(Total = n(), Complete = sum(CompleteCase), Incomplete = n() -
sum(CompleteCase), Total_percent = round(Total / length(Wall2$mergeid), 4),
Prop_incomplete = round(Incomplete / Total , 4)) %>%
  ungroup() %>% as.data.frame() %>% print() %>% filter(!is.na(i05)) %>%
  select(i05, Complete, Incomplete) %>%
  column_to_rownames("i05") %>% chisq.test()
Wall2 %>% group_by(i06) %>%
  summarize(Total = n(), Complete = sum(CompleteCase), Incomplete = n() -
sum(CompleteCase), Total_percent = round(Total / length(Wall2$mergeid), 4),
Prop_incomplete = round(Incomplete / Total , 4)) %>%
  ungroup() %>% as.data.frame() %>% print() %>% filter(!is.na(i06)) %>%
  select(i06, Complete, Incomplete) %>%
```

```
  column_to_rownames("i06") %>% chisq.test()
Wall2 %>% group_by(i07) %>%
  summarize(Total = n(), Complete = sum(CompleteCase), Incomplete = n() -
sum(CompleteCase), Total_percent = round(Total / length(Wall2$mergeid), 4),
Prop_incomplete = round(Incomplete / Total , 4)) %>%
  ungroup() %>% as.data.frame() %>% print() %>% filter(!is.na(i07)) %>%
  select(i07, Complete, Incomplete) %>%
  column_to_rownames("i07") %>% chisq.test()
Wall2 %>% group_by(i08) %>%
  summarize(Total = n(), Complete = sum(CompleteCase), Incomplete = n() -
sum(CompleteCase), Total_percent = round(Total / length(Wall2$mergeid), 4),
Prop_incomplete = round(Incomplete / Total , 4)) %>%
  ungroup() %>% as.data.frame() %>% print() %>% filter(!is.na(i08)) %>%
  select(i08, Complete, Incomplete) %>%
  column_to_rownames("i08") %>% chisq.test()
Wall2 %>% group_by(i09) %>%
  summarize(Total = n(), Complete = sum(CompleteCase), Incomplete = n() -
sum(CompleteCase), Total_percent = round(Total / length(Wall2$mergeid), 4),
Prop_incomplete = round(Incomplete / Total , 4)) %>%
  ungroup() %>% as.data.frame() %>% print() %>% filter(!is.na(i09)) %>%
  select(i09, Complete, Incomplete) %>%
  column_to_rownames("i09") %>% chisq.test()
Wall2 %>% group_by(i10) %>%
  summarize(Total = n(), Complete = sum(CompleteCase), Incomplete = n() -
sum(CompleteCase), Total_percent = round(Total / length(Wall2$mergeid), 4),
Prop_incomplete = round(Incomplete / Total , 4)) %>%
  ungroup() %>% as.data.frame() %>% print() %>% filter(!is.na(i10)) %>%
  select(i10, Complete, Incomplete) %>%
  column_to_rownames("i10") %>% chisq.test()
Wall2 %>% group_by(i11) %>%
  summarize(Total = n(), Complete = sum(CompleteCase), Incomplete = n() -
sum(CompleteCase), Total_percent = round(Total / length(Wall2$mergeid), 4),
Prop_incomplete = round(Incomplete / Total , 4)) %>%
  ungroup() %>% as.data.frame() %>% print() %>% filter(!is.na(i11)) %>%
  select(i11, Complete, Incomplete) %>%
  column_to_rownames("i11") %>% chisq.test()
Wall2 %>% group_by(i12) %>%
  summarize(Total = n(), Complete = sum(CompleteCase), Incomplete = n() -
sum(CompleteCase), Total_percent = round(Total / length(Wall2$mergeid), 4),
Prop_incomplete = round(Incomplete / Total , 4)) %>%
  ungroup() %>% as.data.frame() %>% print() %>% filter(!is.na(i12)) %>%
  select(i12, Complete, Incomplete) %>%
  column_to_rownames("i12") %>% chisq.test()
Wall2 %>% group_by(i13) %>%
  summarize(Total = n(), Complete = sum(CompleteCase), Incomplete = n() -
sum(CompleteCase), Total_percent = round(Total / length(Wall2$mergeid), 4),
Prop_incomplete = round(Incomplete / Total , 4)) %>%
  ungroup() %>% as.data.frame() %>% print() %>% filter(!is.na(i13)) %>%
  select(i13, Complete, Incomplete) %>%
  column_to_rownames("i13") %>% chisq.test()
Wall2 %>% group_by(i14) %>%
  summarize(Total = n(), Complete = sum(CompleteCase), Incomplete = n() -
sum(CompleteCase), Total_percent = round(Total / length(Wall2$mergeid), 4),
Prop_incomplete = round(Incomplete / Total , 4)) %>%
  ungroup() %>% as.data.frame() %>% print() %>% filter(!is.na(i14)) %>%
  select(i14, Complete, Incomplete) %>%
  column_to_rownames("i14") %>% chisq.test()
Wall2 %>% group_by(i15) %>%
```

```
  summarize(Total = n(), Complete = sum(CompleteCase), Incomplete = n() -
sum(CompleteCase), Total_percent = round(Total / length(Wall2$mergeid), 4),
Prop_incomplete = round(Incomplete / Total , 4)) %>%
  ungroup() %>% as.data.frame() %>% print() %>% filter(!is.na(i15)) %>%
  select(i15, Complete, Incomplete) %>%
  column_to_rownames("i15") %>% chisq.test()
Wall2 %>% group_by(i16) %>%
  summarize(Total = n(), Complete = sum(CompleteCase), Incomplete = n() -
sum(CompleteCase), Total_percent = round(Total / length(Wall2$mergeid), 4),
Prop_incomplete = round(Incomplete / Total , 4)) %>%
  ungroup() %>% as.data.frame() %>% print() %>% filter(!is.na(i16)) %>%
  select(i16, Complete, Incomplete) %>%
  column_to_rownames("i16") %>% chisq.test()
Wall2 %>% group_by(i17) %>%
  summarize(Total = n(), Complete = sum(CompleteCase), Incomplete = n() -
sum(CompleteCase), Total_percent = round(Total / length(Wall2$mergeid), 4),
Prop_incomplete = round(Incomplete / Total , 4)) %>%
  ungroup() %>% as.data.frame() %>% print() %>% filter(!is.na(i17)) %>%
  select(i17, Complete, Incomplete) %>%
  column_to_rownames("i17") %>% chisq.test()
Wall2 %>% group_by(i18) %>%
  summarize(Total = n(), Complete = sum(CompleteCase), Incomplete = n() -
sum(CompleteCase), Total_percent = round(Total / length(Wall2$mergeid), 4),
Prop_incomplete = round(Incomplete / Total , 4)) %>%
  ungroup() %>% as.data.frame() %>% print() %>% filter(!is.na(i18)) %>%
  select(i18, Complete, Incomplete) %>%
  column_to_rownames("i18") %>% chisq.test()
Wall2 %>% group_by(i19) %>%
  summarize(Total = n(), Complete = sum(CompleteCase), Incomplete = n() -
sum(CompleteCase), Total_percent = round(Total / length(Wall2$mergeid), 4),
Prop_incomplete = round(Incomplete / Total , 4)) %>%
  ungroup() %>% as.data.frame() %>% print() %>% filter(!is.na(i19)) %>%
  select(i19, Complete, Incomplete) %>%
  column_to_rownames("i19") %>% chisq.test()
Wall2 %>% group_by(i20) %>%
  summarize(Total = n(), Complete = sum(CompleteCase), Incomplete = n() -
sum(CompleteCase), Total_percent = round(Total / length(Wall2$mergeid), 4),
Prop_incomplete = round(Incomplete / Total , 4)) %>%
  ungroup() %>% as.data.frame() %>% print() %>% filter(!is.na(i20)) %>%
  select(i20, Complete, Incomplete) %>%
  column_to_rownames("i20") %>% chisq.test()
Wall2 %>% group_by(i21) %>%
  summarize(Total = n(), Complete = sum(CompleteCase), Incomplete = n() -
sum(CompleteCase), Total_percent = round(Total / length(Wall2$mergeid), 4),
Prop_incomplete = round(Incomplete / Total , 4)) %>%
  ungroup() %>% as.data.frame() %>% print() %>% filter(!is.na(i21)) %>%
  select(i21, Complete, Incomplete) %>%
  column_to_rownames("i21") %>% chisq.test()
Wall2 %>% group_by(i22) %>%
  summarize(Total = n(), Complete = sum(CompleteCase), Incomplete = n() -
sum(CompleteCase), Total_percent = round(Total / length(Wall2$mergeid), 4),
Prop_incomplete = round(Incomplete / Total , 4)) %>%
  ungroup() %>% as.data.frame() %>% print() %>% filter(!is.na(i22)) %>%
  select(i22, Complete, Incomplete) %>%
  column_to_rownames("i22") %>% chisq.test()
Wall2 %>% group_by(i23) %>%
  summarize(Total = n(), Complete = sum(CompleteCase), Incomplete = n() -
sum(CompleteCase), Total_percent = round(Total / length(Wall2$mergeid), 4),
Prop_incomplete = round(Incomplete / Total , 4)) %>%
```

```
  ungroup() %>% as.data.frame() %>% print() %>% filter(!is.na(i23)) %>%
  select(i23, Complete, Incomplete) %>%
  column_to_rownames("i23") %>% chisq.test()
Wall2 %>% group_by(i24) %>%
  summarize(Total = n(), Complete = sum(CompleteCase), Incomplete = n() -
sum(CompleteCase), Total_percent = round(Total / length(Wall2$mergeid), 4),
Prop_incomplete = round(Incomplete / Total , 4)) %>%
  ungroup() %>% as.data.frame() %>% print() %>% filter(!is.na(i24)) %>%
  select(i24, Complete, Incomplete) %>%
  column_to_rownames("i24") %>% chisq.test()
Wall2 %>% group_by(i25) %>%
  summarize(Total = n(), Complete = sum(CompleteCase), Incomplete = n() -
sum(CompleteCase), Total_percent = round(Total / length(Wall2$mergeid), 4),
Prop_incomplete = round(Incomplete / Total , 4)) %>%
  ungroup() %>% as.data.frame() %>% print() %>% filter(!is.na(i25)) %>%
  select(i25, Complete, Incomplete) %>%
  column_to_rownames("i25") %>% chisq.test()
Wall2 %>% group_by(i26) %>%
  summarize(Total = n(), Complete = sum(CompleteCase), Incomplete = n() -
sum(CompleteCase), Total_percent = round(Total / length(Wall2$mergeid), 4),
Prop_incomplete = round(Incomplete / Total , 4)) %>%
  ungroup() %>% as.data.frame() %>% print() %>% filter(!is.na(i26)) %>%
  select(i26, Complete, Incomplete) %>%
  column_to_rownames("i26") %>% chisq.test()
Wall2 %>% group_by(i27) %>%
  summarize(Total = n(), Complete = sum(CompleteCase), Incomplete = n() -
sum(CompleteCase), Total_percent = round(Total / length(Wall2$mergeid), 4),
Prop_incomplete = round(Incomplete / Total , 4)) %>%
  ungroup() %>% as.data.frame() %>% print() %>% filter(!is.na(i27)) %>%
  select(i27, Complete, Incomplete) %>%
  column_to_rownames("i27") %>% chisq.test()
Wall2 %>% group_by(i28) %>%
  summarize(Total = n(), Complete = sum(CompleteCase), Incomplete = n() -
sum(CompleteCase), Total_percent = round(Total / length(Wall2$mergeid), 4),
Prop_incomplete = round(Incomplete / Total , 4)) %>%
  ungroup() %>% as.data.frame() %>% print() %>% filter(!is.na(i28)) %>%
  select(i28, Complete, Incomplete) %>%
  column_to_rownames("i28") %>% chisq.test()
Wall2 %>% group_by(i29) %>%
  summarize(Total = n(), Complete = sum(CompleteCase), Incomplete = n() -
sum(CompleteCase), Total_percent = round(Total / length(Wall2$mergeid), 4),
Prop_incomplete = round(Incomplete / Total , 4)) %>%
  ungroup() %>% as.data.frame() %>% print() %>% filter(!is.na(i29)) %>%
  select(i29, Complete, Incomplete) %>%
  column_to_rownames("i29") %>% chisq.test()
Wall2 %>% group_by(i30) %>%
  summarize(Total = n(), Complete = sum(CompleteCase), Incomplete = n() -
sum(CompleteCase), Total_percent = round(Total / length(Wall2$mergeid), 4),
Prop_incomplete = round(Incomplete / Total , 4)) %>%
  ungroup() %>% as.data.frame() %>% print() %>% filter(!is.na(i30)) %>%
  select(i30, Complete, Incomplete) %>%
  column_to_rownames("i30") %>% chisq.test()
Wall2 %>% group_by(i31) %>%
  summarize(Total = n(), Complete = sum(CompleteCase), Incomplete = n() -
sum(CompleteCase), Total_percent = round(Total / length(Wall2$mergeid), 4),
Prop_incomplete = round(Incomplete / Total , 4)) %>%
  ungroup() %>% as.data.frame() %>% print() %>% filter(!is.na(i31)) %>%
  select(i31, Complete, Incomplete) %>%
  column_to_rownames("i31") %>% chisq.test()
```

```
Wall2 %>% group_by(i32) %>%
  summarize(Total = n(), Complete = sum(CompleteCase), Incomplete = n() -
sum(CompleteCase), Total_percent = round(Total / length(Wall2$mergeid), 4),
Prop_incomplete = round(Incomplete / Total , 4)) %>%
  ungroup() %>% as.data.frame() %>% print() %>% filter(!is.na(i32)) %>%
  select(i32, Complete, Incomplete) %>%
  column_to_rownames("i32") %>% chisq.test()
Wall2 %>% group_by(i33) %>%
  summarize(Total = n(), Complete = sum(CompleteCase), Incomplete = n() -
sum(CompleteCase), Total_percent = round(Total / length(Wall2$mergeid), 4),
Prop_incomplete = round(Incomplete / Total , 4)) %>%
  ungroup() %>% as.data.frame() %>% print() %>% filter(!is.na(i33)) %>%
  select(i33, Complete, Incomplete) %>%
  column_to_rownames("i33") %>% chisq.test()
Wall2 %>% group_by(i34) %>%
  summarize(Total = n(), Complete = sum(CompleteCase), Incomplete = n() -
sum(CompleteCase), Total_percent = round(Total / length(Wall2$mergeid), 4),
Prop_incomplete = round(Incomplete / Total , 4)) %>%
  ungroup() %>% as.data.frame() %>% print() %>% filter(!is.na(i34)) %>%
  select(i34, Complete, Incomplete) %>%
  column_to_rownames("i34") %>% chisq.test()
Wall2 %>% group_by(i35) %>%
  summarize(Total = n(), Complete = sum(CompleteCase), Incomplete = n() -
sum(CompleteCase), Total_percent = round(Total / length(Wall2$mergeid), 4),
Prop_incomplete = round(Incomplete / Total , 4)) %>%
  ungroup() %>% as.data.frame() %>% print() %>% filter(!is.na(i35)) %>%
  select(i35, Complete, Incomplete) %>%
  column_to_rownames("i35") %>% chisq.test()
Wall2 %>% group_by(i36) %>%
  summarize(Total = n(), Complete = sum(CompleteCase), Incomplete = n() -
sum(CompleteCase), Total_percent = round(Total / length(Wall2$mergeid), 4),
Prop_incomplete = round(Incomplete / Total , 4)) %>%
  ungroup() %>% as.data.frame() %>% print() %>% filter(!is.na(i36)) %>%
  select(i36, Complete, Incomplete) %>%
  column_to_rownames("i36") %>% chisq.test()
Wall2 %>% group_by(i37) %>%
  summarize(Total = n(), Complete = sum(CompleteCase), Incomplete = n() -
sum(CompleteCase), Total_percent = round(Total / length(Wall2$mergeid), 4),
Prop_incomplete = round(Incomplete / Total , 4)) %>%
  ungroup() %>% as.data.frame() %>% print() %>% filter(!is.na(i37)) %>%
  select(i37, Complete, Incomplete) %>%
  column_to_rownames("i37") %>% chisq.test()
Wall2 %>% group_by(i38) %>%
  summarize(Total = n(), Complete = sum(CompleteCase), Incomplete = n() -
sum(CompleteCase), Total_percent = round(Total / length(Wall2$mergeid), 4),
Prop_incomplete = round(Incomplete / Total , 4)) %>%
  ungroup() %>% as.data.frame() %>% print() %>% filter(!is.na(i38)) %>%
  select(i38, Complete, Incomplete) %>%
  column_to_rownames("i38") %>% chisq.test()
Wall2 %>% group_by(i39) %>%
  summarize(Total = n(), Complete = sum(CompleteCase), Incomplete = n() -
sum(CompleteCase), Total_percent = round(Total / length(Wall2$mergeid), 4),
Prop_incomplete = round(Incomplete / Total , 4)) %>%
  ungroup() %>% as.data.frame() %>% print() %>% filter(!is.na(i39)) %>%
  select(i39, Complete, Incomplete) %>%
  column_to_rownames("i39") %>% chisq.test()
Wall2 %>% group_by(i40) %>%
```

```
  summarize(Total = n(), Complete = sum(CompleteCase), Incomplete = n() -
sum(CompleteCase), Total_percent = round(Total / length(Wall2$mergeid), 4),
Prop_incomplete = round(Incomplete / Total , 4)) %>%
  ungroup() %>% as.data.frame() %>% print() %>% filter(!is.na(i40)) %>%
  select(i40, Complete, Incomplete) %>%
  column_to_rownames("i40") %>% chisq.test()
#######

# Table 1. Study population by countries (number of surveys / number of subjects)

T1c <-  Wall_i2 %>% filter(.imp >0) %>%
  group_by(country) %>%
  summarize(
    Full_surveys = n()/10,
    Full_subjects = length(unique(mergeid))) %>%
  ungroup() %>%
  as.data.frame()

T1d <- Wall_c %>%
  group_by(country) %>%
  summarize(
    Complete_surveys = n(),
    Complete_subjects = length(unique(mergeid))) %>%
  ungroup() %>%
  as.data.frame()

T1 <- T1c %>% left_join(T1d)
T1[length(T1$country)+1,] <- c("Total",
                               sum(T1$Full_surveys),
                               sum(T1$Full_subjects),
                               sum(T1$Complete_surveys, na.rm = T),
                               sum(T1$Complete_subjects, na.rm = T)) # data for
the TOTAL row

### Table 2. Study population characteristics
#Gender
table(Wall2$gender)
table(Wall2$gender[Wall2$CompleteCase == T])
#Age
summary(Wall2$Age); IQR(Wall2$Age)
summary(Wall2$Age[Wall2$CompleteCase == T]); IQR(Wall2$Age[Wall2$CompleteCase ==
T])
#Education
Wall2 %>% select(mergeid, Wave) %>% left_join(EDU_table2) %>% select(Edu_y_i) %>%
summary(na.rm = T)
Wall2 %>% select(mergeid, Wave, CompleteCase) %>% left_join(EDU_table2) %>%
filter(CompleteCase == T) %>% select(Edu_y_i) %>% summary(na.rm = T)
#Marital
table(Wall_i$MaritalStatus) / length(Wall_i$MaritalStatus)
table(Wall_i$MaritalStatus[Wall_i$CompleteCase == T]) /
length(Wall_i$MaritalStatus[Wall_i$CompleteCase == T])
#Self-perceived health
table(Wall_i$Health) / length(Wall_i$Health)
table(Wall_i$Health[Wall_i$CompleteCase == T]) /
length(Wall_i$Health[Wall_i$CompleteCase == T])

### Figure 2. Frailty rates by age and sex: complete cases vs all cases
Fig2data_index <- Summary_i %>% select(Agebands, gender, N_total, N_frail) %>%
group_by(Agebands, gender) %>%
```

```
  summarize(
    N_total = sum(N_total), N_frail = sum(N_frail), Prop_frail = N_frail /
N_total,
    Prop_frail_CI_lower = exactci(N_frail, N_total, 0.95)[[1]][1],
    Prop_frail_CI_upper = exactci(N_frail, N_total, 0.95)[[1]][2],
    Missingness = "Multiple imputation",
    Frailty_method = "Frailty Index") %>%
  ungroup %>% as.data.frame() %>% rbind(
    Summary_c %>% select(Agebands, gender, N_total, N_frail) %>%
group_by(Agebands, gender) %>%
      summarize(
        N_total = sum(N_total), N_frail = sum(N_frail), Prop_frail = N_frail /
N_total,
        Prop_frail_CI_lower = exactci(N_frail, N_total, 0.95)[[1]][1],
        Prop_frail_CI_upper = exactci(N_frail, N_total, 0.95)[[1]][2],
        Missingness = "Observed cases", Frailty_method = "Frailty Index") %>%
ungroup %>% as.data.frame())
write.csv(Fig2data_index, "Fig2data_index_20230927.csv")

### Figure 3. Frailty rates by age, gender, and GDP/capita kEUR
Fig3data_Index_cc <- Summary_c %>%
  select(country, Agebands, gender, GDP_cap_k, Prop_frail, Predict_prob, N_total)
%>%
  mutate(Analysis = "Complete cases", Frailty_method = "Frailty Index")
Fig3data_Index_imp <- Summary_i %>%
  select(country, Agebands, gender, GDP_cap_k, Prop_frail, Predict_prob, N_total)
%>%
  mutate(Analysis = "Full sample", Frailty_method = "Frailty Index")
Fig3data_Index <- rbind(Fig3data_Index_cc, Fig3data_Index_imp)
write.csv(Fig3data_Index, "Fig3data_Index_20230927.csv")


### Figure 4. Frailty rates by age, gender, and GDP/capita PPP
Fig4data_Index_cc <- Summary_c %>%
  select(country, Agebands, gender, GDP_cap_PPP_k, Prop_frail, Predict_prob_ppp,
N_total) %>%
  mutate(Analysis = "Complete cases", Frailty_method = "Frailty Index")
Fig4data_Index_imp <- Summary_i %>%
  select(country, Agebands, gender, GDP_cap_PPP_k, Prop_frail, Predict_prob_ppp,
N_total) %>%
  mutate(Analysis = "Full sample", Frailty_method = "Frailty Index")
Fig4data_Index <- rbind(Fig4data_Index_cc, Fig4data_Index_imp)
write.csv(Fig4data_Index, "Fig4data_Index_20230927.csv")
###############################
```

Statistical analysis script for the Frailty Instrument analyses

```
# Suppl material
# SHARE FRAILTY INSTRUMENT ANALYSES
# 0. Housekeeping
###############################
library(foreign)
library(dplyr)
library(lubridate)
library(stringr)
library(tidyr)
library(ggplot2)
library(ggthemes)
library(ggthemes)
```

```
library(naniar)
library(PropCIs)
library(lme4)
library(broom.mixed)
library(mice)
library(tibble)

setwd("C:/Users/janos.pitter/OneDrive - SYREON Kft/Safe Syreon/Munka/Frailty/2023Q1 SHARE
GDP dependence")
# set your working directory where the SHARE data tables are saved (downloaded as Stata files)

################################

#  1. Data load
################################
# Wave 1
W1_cv_r <- read.dta("sharew1_rel8-0-0_cv_r.dta", missing.type = T) %>% as.data.frame() %>%
select(mergeid, country, gender, yrbirth, mobirth, age2004, age_int, int_year, int_month)
W1_gv_health <- read.dta("sharew1_rel8-0-0_gv_health.dta", missing.type = T) %>% as.data.frame()
%>% select(mergeid, bmi, bmi2, maxgrip)
W1_ph <- read.dta("sharew1_rel8-0-0_ph.dta", missing.type = T) %>% as.data.frame() %>%
select(mergeid, ph048d1, ph048d5)
W1_br <- read.dta("sharew1_rel8-0-0_br.dta", missing.type = T) %>% as.data.frame() %>%
select(mergeid, br015_, br016_)
W1_mh <- read.dta("sharew1_rel8-0-0_mh.dta", missing.type = T) %>% as.data.frame() %>%
select(mergeid, mh011_, mh012_, mh013_)
W1 <- W1_cv_r %>% left_join(W1_gv_health) %>% left_join(W1_ph) %>% left_join(W1_br) %>%
left_join(W1_mh) %>% filter(int_year != -9)  # Interviews with int_year = -9 were not conducted,
these are not valid cases and are removed
rm(W1_br, W1_cv_r, W1_gv_health, W1_mh, W1_ph)
# define age
W1$mobirth_num <- match(W1$mobirth, month.name); table(W1$mobirth_num, W1$mobirth,
useNA = "always")
W1$int_month_num <- match(W1$int_month, month.name); table(W1$int_month_num,
W1$int_month, useNA = "always")
W1$Age <- ifelse(W1$age_int>0, W1$age_int, ifelse(
  W1$age2004>0, W1$age2004, ifelse(
    (is.na(W1$int_month_num)|is.na(W1$mobirth_num))& W1$yrbirth>0, 2004-W1$yrbirth, ifelse(
      W1$mobirth_num <= W1$int_month_num & W1$yrbirth>0, 2004-W1$yrbirth, ifelse(
        W1$mobirth_num > W1$int_month_num & W1$yrbirth>0, 2004 - W1$yrbirth - 1, NA)))))
summary(W1$Age)
W1 <- W1 %>% filter(W1$Age >= 50 & !is.na(W1$Age))
W1$Agebands <- paste0(floor(W1$Age/5)*5, " to ", floor(W1$Age/5)*5+4); W1$Agebands[W1$Age
>= 90] <- "90 or higher"
W1$Agebands <- W1$Agebands %>% factor(levels= sort(unique(W1$Agebands)));
table(W1$Agebands, useNA = "always")
W1$Wave <- "W1"
W1 <- W1 %>% data.frame() %>% select(mergeid, Wave, country, gender, Age, Agebands, int_year,
bmi, bmi2, maxgrip, ph048d1, ph048d5, br015_, br016_, mh011_, mh012_, mh013_)

# Wave 2
```

```
W2_cv_r <- read.dta("sharew2_rel8-0-0_cv_r.dta", missing.type = T) %>% as.data.frame() %>%
select(mergeid, country, gender, yrbirth, mobirth, age2007, age_int, int_year, int_month)
W2_gv_health <- read.dta("sharew2_rel8-0-0_gv_health.dta", missing.type = T) %>% as.data.frame()
%>% select(mergeid, bmi, bmi2, maxgrip)
W2_ph <- read.dta("sharew2_rel8-0-0_ph.dta", missing.type = T) %>% as.data.frame() %>%
select(mergeid, ph048d1, ph048d5)
W2_br <- read.dta("sharew2_rel8-0-0_br.dta", missing.type = T) %>% as.data.frame() %>%
select(mergeid, br015_, br016_)
W2_mh <- read.dta("sharew2_rel8-0-0_mh.dta", missing.type = T) %>% as.data.frame() %>%
select(mergeid, mh011_, mh012_, mh013_)
W2 <- W2_cv_r %>% left_join(W2_gv_health) %>% left_join(W2_ph) %>% left_join(W2_br) %>%
left_join(W2_mh) %>% filter(int_year != -9)  # Interviews with int_year = -9 were not conducted,
these are not valid cases and are removed
rm(W2_br, W2_cv_r, W2_gv_health, W2_mh, W2_ph)
# define age
W2$mobirth_num <- match(W2$mobirth, month.name); # table(W2$mobirth_num, W2$mobirth,
useNA = "always")
W2$int_month_num <- match(W2$int_month, month.name); # table(W2$int_month_num,
W2$int_month, useNA = "always")
W2$Age <- ifelse(W2$age_int>0, W2$age_int, ifelse(
  W2$age2007>0, W2$age2007, ifelse(
   (is.na(W2$int_month_num)|is.na(W2$mobirth_num))& W2$yrbirth>0, 2007-W2$yrbirth, ifelse(
    W2$mobirth_num <= W2$int_month_num & W2$yrbirth>0, 2007-W2$yrbirth, ifelse(
     W2$mobirth_num > W2$int_month_num & W2$yrbirth>0, 2007 - W2$yrbirth - 1, NA)))))
summary(W2$Age)
W2 <- W2 %>% filter(W2$Age >= 50 & !is.na(W2$Age))
W2$Agebands <- paste0(floor(W2$Age/5)*5, " to ", floor(W2$Age/5)*5+4); W2$Agebands[W2$Age
>= 90] <- "90 or higher"
W2$Agebands <- W2$Agebands %>% factor(levels= sort(unique(W2$Agebands)))
table(W2$Agebands, useNA = "always")
W2$hhid2 <- paste0("W2_", W2$hhid2); names(W2)[names(W2) == "hhid2"] <- "W_hhid"
W2$Wave <- "W2"
W2 <- W2 %>% data.frame() %>% select(names(W1))

# Wave 4
W4_cv_r <- read.dta("sharew4_rel8-0-0_cv_r.dta", missing.type = T) %>% as.data.frame() %>%
select(mergeid, country, gender, yrbirth, mobirth, age2011, age_int, int_year, int_month)
W4_gv_health <- read.dta("sharew4_rel8-0-0_gv_health.dta", missing.type = T) %>% as.data.frame()
%>% select(mergeid, bmi, bmi2, maxgrip)
W4_ph <- read.dta("sharew4_rel8-0-0_ph.dta", missing.type = T) %>% as.data.frame() %>%
select(mergeid, ph048d1, ph048d5)
W4_br <- read.dta("sharew4_rel8-0-0_br.dta", missing.type = T) %>% as.data.frame() %>%
select(mergeid, br015_, br016_)
W4_mh <- read.dta("sharew4_rel8-0-0_mh.dta", missing.type = T) %>% as.data.frame() %>%
select(mergeid, mh011_, mh012_, mh013_)
W4 <- W4_cv_r %>% left_join(W4_gv_health) %>% left_join(W4_ph) %>% left_join(W4_br) %>%
left_join(W4_mh) %>% filter(int_year != -9)  # Interviews with int_year = -9 were not conducted,
these are not valid cases and are removed
rm(W4_br, W4_cv_r, W4_gv_health, W4_mh, W4_ph)
# define age
```

```
W4$mobirth_num <- match(W4$mobirth, month.name); # table(W4$mobirth_num, W4$mobirth,
useNA = "always")
W4$int_month_num <- match(W4$int_month, month.name); # table(W4$int_month_num,
W4$int_month, useNA = "always")
W4$Age <- ifelse(W4$age_int>0, W4$age_int, ifelse(
  W4$age2011>0, W4$age2011, ifelse(
    (is.na(W4$int_month_num)|is.na(W4$mobirth_num))& W4$yrbirth>0, 2011-W4$yrbirth, ifelse(
      W4$mobirth_num <= W4$int_month_num & W4$yrbirth>0, 2011-W4$yrbirth, ifelse(
        W4$mobirth_num > W4$int_month_num & W4$yrbirth>0, 2011 - W4$yrbirth - 1, NA)))))
summary(W4$Age)
W4 <- W4 %>% filter(W4$Age >= 50 & !is.na(W4$Age))
W4$Agebands <- paste0(floor(W4$Age/5)*5, " to ", floor(W4$Age/5)*5+4); W4$Agebands[W4$Age
>= 90] <- "90 or higher"
W4$Agebands <- W4$Agebands %>% factor(levels= sort(unique(W4$Agebands)));
table(W4$Agebands, useNA = "always")
W4$Wave <- "W4"
W4 <- W4 %>% data.frame() %>% select(names(W1))

# Wave 5
W5_cv_r <- read.dta("sharew5_rel8-0-0_cv_r.dta", missing.type = T) %>% as.data.frame() %>%
select(mergeid, country, gender, yrbirth, mobirth, age2013, age_int, int_year, int_month)
W5_gv_health <- read.dta("sharew1_rel8-0-0_gv_health.dta", missing.type = T) %>% as.data.frame()
%>% select(mergeid, bmi, bmi2, maxgrip)
W5_ph <- read.dta("sharew5_rel8-0-0_ph.dta", missing.type = T) %>% as.data.frame() %>%
select(mergeid, ph048d1, ph048d5)
W5_br <- read.dta("sharew5_rel8-0-0_br.dta", missing.type = T) %>% as.data.frame() %>%
select(mergeid, br015_, br016_)
W5_mh <- read.dta("sharew5_rel8-0-0_mh.dta", missing.type = T) %>% as.data.frame() %>%
select(mergeid, mh011_, mh012_, mh013_)
W5 <- W5_cv_r %>% left_join(W5_gv_health) %>% left_join(W5_ph) %>% left_join(W5_br) %>%
left_join(W5_mh) %>% filter(int_year != -9)  # Interviews with int_year = -9 were not conducted,
these are not valid cases and are removed
rm(W5_br, W5_cv_r, W5_gv_health, W5_mh, W5_ph)
# define age
W5$mobirth_num <- match(W5$mobirth, month.name); # table(W5$mobirth_num, W5$mobirth,
useNA = "always")
W5$int_month_num <- match(W5$int_month, month.name); # table(W5$int_month_num,
W5$int_month, useNA = "always")
W5$Age <- ifelse(W5$age_int>0, W5$age_int, ifelse(
  W5$age2013>0, W5$age2013, ifelse(
    (is.na(W5$int_month_num)|is.na(W5$mobirth_num))& W5$yrbirth>0, 2013-W5$yrbirth, ifelse(
      W5$mobirth_num <= W5$int_month_num & W5$yrbirth>0, 2013-W5$yrbirth, ifelse(
        W5$mobirth_num > W5$int_month_num & W5$yrbirth>0, 2013 - W5$yrbirth - 1, NA)))))
summary(W5$Age)
W5 <- W5 %>% filter(W5$Age >= 50 & !is.na(W5$Age))
W5$Agebands <- paste0(floor(W5$Age/5)*5, " to ", floor(W5$Age/5)*5+4); W5$Agebands[W5$Age
>= 90] <- "90 or higher"
W5$Agebands <- W5$Agebands %>% factor(levels= sort(unique(W5$Agebands)));
table(W5$Agebands, useNA = "always")
W5$Wave <- "W5"
W5 <- W5 %>% data.frame() %>% select(names(W1))
```

```
# Wave 6
W6_cv_r <- read.dta("sharew6_rel8-0-0_cv_r.dta", missing.type = T) %>% as.data.frame() %>%
select(mergeid, country, gender, yrbirth, mobirth, age2015, age_int, int_year, int_month)
W6_gv_health <- read.dta("sharew6_rel8-0-0_gv_health.dta", missing.type = T) %>% as.data.frame()
%>% select(mergeid, bmi, bmi2, maxgrip)
W6_ph <- read.dta("sharew6_rel8-0-0_ph.dta", missing.type = T) %>% as.data.frame() %>%
select(mergeid, ph048d1, ph048d5)
W6_br <- read.dta("sharew6_rel8-0-0_br.dta", missing.type = T) %>% as.data.frame() %>%
select(mergeid, br015_, br016_)
W6_mh <- read.dta("sharew6_rel8-0-0_mh.dta", missing.type = T) %>% as.data.frame() %>%
select(mergeid, mh011_, mh012_, mh013_)
W6 <- W6_cv_r %>% left_join(W6_gv_health) %>% left_join(W6_ph) %>% left_join(W6_br) %>%
left_join(W6_mh) %>% filter(int_year != -9)  # Interviews with int_year = -9 were not conducted,
these are not valid cases and are removed
rm(W6_br, W6_cv_r, W6_gv_health, W6_mh, W6_ph)
# define age
W6$mobirth_num <- match(W6$mobirth, month.name); # table(W6$mobirth_num, W6$mobirth,
useNA = "always")
W6$int_month_num <- match(W6$int_month, month.name); # table(W6$int_month_num,
W6$int_month, useNA = "always")
W6$Age <- ifelse(W6$age_int>0, W6$age_int, ifelse(
  W6$age2015>0, W6$age2015, ifelse(
   (is.na(W6$int_month_num)|is.na(W6$mobirth_num))& W6$yrbirth>0, 2015-W6$yrbirth, ifelse(
    W6$mobirth_num <= W6$int_month_num & W6$yrbirth>0, 2015 - W6$yrbirth, ifelse(
     W6$mobirth_num > W6$int_month_num & W6$yrbirth>0, 2015 - W6$yrbirth - 1, NA)))))
summary(W6$Age)
W6 <- W6 %>% filter(W6$Age >= 50 & !is.na(W6$Age))
W6$Agebands <- paste0(floor(W6$Age/5)*5, " to ", floor(W6$Age/5)*5+4); W6$Agebands[W6$Age
>= 90] <- "90 or higher"
W6$Agebands <- W6$Agebands %>% factor(levels= sort(unique(W6$Agebands)));
table(W6$Agebands, useNA = "always")
W6$Wave <- "W6"
W6 <- W6 %>% data.frame() %>% select(names(W1))

# Wave 7
W7_cv_r <- read.dta("sharew7_rel8-0-0_cv_r.dta", missing.type = T) %>% as.data.frame() %>%
select(mergeid, country, gender, yrbirth, mobirth, age2017, age_int, int_year, int_month)
W7_gv_health <- read.dta("sharew7_rel8-0-0_gv_health.dta", missing.type = T) %>% as.data.frame()
%>% select(mergeid, bmi, bmi2, maxgrip)
W7_ph <- read.dta("sharew7_rel8-0-0_ph.dta", missing.type = T) %>% as.data.frame() %>%
select(mergeid, ph048d1, ph048d5)
W7_br <- read.dta("sharew7_rel8-0-0_br.dta", missing.type = T) %>% as.data.frame() %>%
select(mergeid, br015_, br016_)
W7_mh <- read.dta("sharew7_rel8-0-0_mh.dta", missing.type = T) %>% as.data.frame() %>%
select(mergeid, mh011_, mh012_, mh013_)
W7 <- W7_cv_r %>% left_join(W7_gv_health) %>% left_join(W7_ph) %>% left_join(W7_br) %>%
left_join(W7_mh) %>% filter(int_year != -9)  # Interviews with int_year = -9 were not conducted,
these are not valid cases and are removed
rm(W7_br, W7_cv_r, W7_gv_health, W7_mh, W7_ph)
# define age
```

```
W7$mobirth_num <- match(W7$mobirth, month.name); # table(W7$mobirth_num, W7$mobirth,
useNA = "always")
W7$int_month_num <- match(W7$int_month, month.name); # table(W7$int_month_num,
W7$int_month, useNA = "always")
W7$Age <- ifelse(W7$age_int>0, W7$age_int, ifelse(
  W7$age2017>0, W7$age2017, ifelse(
    (is.na(W7$int_month_num)|is.na(W7$mobirth_num))& W7$yrbirth>0, 2017-W7$yrbirth, ifelse(
      W7$mobirth_num <= W7$int_month_num & W7$yrbirth>0, 2017-W7$yrbirth, ifelse(
        W7$mobirth_num > W7$int_month_num & W7$yrbirth>0, 2017 - W7$yrbirth - 1, NA)))))
summary(W7$Age)
W7 <- W7 %>% filter(W7$Age >= 50 & !is.na(W7$Age))
W7$Agebands <- paste0(floor(W7$Age/5)*5, " to ", floor(W7$Age/5)*5+4); W7$Agebands[W7$Age
>= 90] <- "90 or higher"
W7$Agebands <- W7$Agebands %>% factor(levels= sort(unique(W7$Agebands)));
table(W7$Agebands, useNA = "always")
W7$Wave <- "W7"
W7 <- W7 %>% data.frame() %>% select(names(W1))

# Wave 8
W8_cv_r <- read.dta("sharew8_rel8-0-0_cv_r.dta", missing.type = T) %>% as.data.frame() %>%
select(mergeid, country, gender, yrbirth, mobirth, age2020, age_int, int_year, int_month)
W8_gv_health <- read.dta("sharew8_rel8-0-0_gv_health.dta", missing.type = T) %>% as.data.frame()
%>% select(mergeid, bmi, bmi2, maxgrip)
W8_ph <- read.dta("sharew8_rel8-0-0_ph.dta", missing.type = T) %>% as.data.frame() %>%
select(mergeid, ph048d1, ph048d5)
W8_br <- read.dta("sharew8_rel8-0-0_br.dta", missing.type = T) %>% as.data.frame() %>%
select(mergeid, br015_, br016_)
W8_mh <- read.dta("sharew8_rel8-0-0_mh.dta", missing.type = T) %>% as.data.frame() %>%
select(mergeid, mh011_, mh012_, mh013_)
W8 <- W8_cv_r %>% left_join(W8_gv_health) %>% left_join(W8_ph) %>% left_join(W8_br) %>%
left_join(W8_mh) %>% filter(int_year != -9)  # Interviews with int_year = -9 were not conducted,
these are not valid cases and are removed
rm(W8_br, W8_cv_r, W8_gv_health, W8_mh, W8_ph)
# define age
W8$mobirth_num <- match(W8$mobirth, month.name); # table(W8$mobirth_num, W8$mobirth,
useNA = "always")
W8$int_month_num <- match(W8$int_month, month.name); # table(W8$int_month_num,
W8$int_month, useNA = "always")
W8$Age <- ifelse(W8$age_int>0, W8$age_int, ifelse(
  W8$age2020>0, W8$age2020, ifelse(
    (is.na(W8$int_month_num)|is.na(W8$mobirth_num))& W8$yrbirth>0, 2020-W8$yrbirth, ifelse(
      W8$mobirth_num <= W8$int_month_num & W8$yrbirth>0, 2020-W8$yrbirth, ifelse(
        W8$mobirth_num > W8$int_month_num & W8$yrbirth>0, 2020 - W8$yrbirth - 1, NA)))))
summary(W8$Age)
W8 <- W8 %>% filter(W8$Age >= 50 & !is.na(W8$Age))
W8$Agebands <- paste0(floor(W8$Age/5)*5, " to ", floor(W8$Age/5)*5+4); W8$Agebands[W8$Age
>= 90] <- "90 or higher"
W8$Agebands <- W8$Agebands %>% factor(levels= sort(unique(W8$Agebands)));
table(W8$Agebands, useNA = "always")
W8$Wave <- "W8"
W8 <- W8 %>% data.frame() %>% select(names(W1))
```

```
# Merge data from various waves
Wall <- rbind(W1, W2, W4, W5, W6, W7, W8); rm(W1, W2, W4, W5, W6, W7, W8) # all Waves in a merged dataframe

# GDP per capita PPP data from World Bank. First download the file to working directory from:
#
https://data.worldbank.org/indicator/NY.GDP.PCAP.PP.KD?locations=EU&type=shaded&view=map&year=1997
# delete non-European country rows and save the second sheet as csv file.

GDP <- read.csv("GDP_cap_EUR_2000_2004.csv", stringsAsFactors = F) %>%
  select(country, GDP_cap) %>%
  data.frame()
GDP$country[GDP$country == "Czechia"] <- "Czech Republic"
GDP$GDP_cap <- as.numeric(GDP$GDP_cap)
GDP$GDP_cap_k <- GDP$GDP_cap / 1000
GDP <- GDP %>% select(country, GDP_cap_k) %>%
  filter(country %in% c("Turkiye", "T\xfcrkiye", "Turkey", "Liechtenstein") == F)

GDP_PPP <-  read.csv("GDP_cap_EUR_PPP_2000_2004.csv", stringsAsFactors = F) %>%
rename(country = Country) %>% data.frame() # GDP is expressed in Euro purchasoing power parity values in the EU-27: equivalent customer value to 1 EUR in the EU
GDP_PPP$country[GDP_PPP$country == "Czechia"] <- "Czech Republic"
GDP_PPP$GDP_cap_PPP <- as.numeric(GDP_PPP$GDP_cap_PPP)
GDP_PPP$GDP_cap_PPP_k <- GDP_PPP$GDP_cap_PPP / 1000
GDP_PPP <- GDP_PPP %>% select(country, GDP_cap_PPP_k) %>%
  filter(country %in% c("Japan", "Turkiye", "T\xfcrkiye", "Turkey", "Liechtenstein", "United States") == F)


Wall <- Wall %>% mutate(Year = int_year) %>% left_join(GDP) %>% left_join(GDP_PPP)

################################

#  2. Frailty Instrument scores in complete cases
################################
#    as defined by Romero-Ortuno et al. BMC Geriatrics 2010, 10:57.

#    Exhaustion was identified as a positive response to the question: “In the last month, have you had too little energy to do the things you wanted to do?”. A positive answer (Yes) was re-coded as 1, and No was re-coded as 0.
Wall$Fatigue <- ifelse(!is.na(Wall$mh013_) & Wall$mh013_ == "Yes", 1, ifelse(!is.na(Wall$mh013_) & Wall$mh013_ == "No",0, NA))
table(Wall$mh013_, Wall$Fatigue, useNA = "always")

#    The weight loss criterion was fulfilled by reporting a “Diminution in desire for food” in response to the question: “What has your appetite been like?” or, in the case of a non-specific or uncodeable response to this question, by responding “Less” to the question:
#    “So, have you been eating more or less than usual?”. The presence of the criterion was coded as 1 and its absence as 0.
```

Wall$Loss_of_appetite <- ifelse(
  (is.na(Wall$mh011_)|Wall$mh011_ == "Refusal"|Wall$mh011_ == "Don't know"), NA, ifelse(
    Wall$mh011_ == "Diminution in desire for food"|(Wall$mh011_ == "Non-specific or uncodeable response" & !is.na(Wall$mh012_) & Wall$mh012_ == "Less"), 1,ifelse(
      Wall$mh011_ == "Non-specific or uncodeable response" & (is.na(Wall$mh012_)|Wall$mh012_ == "Refusal"|Wall$mh012_ == "Don't know"), NA, 0)))
table(Wall$mh011_, Wall$mh012_, Wall$Loss_of_appetite, useNA = "always")

Weakness was assessed by handgrip strength (Kg) using a dynamometer. Two consecutive measurements were taken from the left and right hands. The highest of the four was selected. This variable was kept continuous.
Wall$Gripstrength <- ifelse(!is.na(Wall$maxgrip) & Wall$maxgrip>0, Wall$maxgrip, NA)
summary(Wall$Gripstrength)

Slowness was defined as a positive answer to either of the following two items: "Because of a health problem, do you have difficulty [expected to last more than 3 months] walking 100 metres?" or "... climbing one flight of stairs without resting?". One or two positive answers received the score of 1, and two negative answers received the score of 0.
Wall$walking_difficulty <- ifelse(!is.na(Wall$ph048d1) & Wall$ph048d1 == "Selected", 1, ifelse(!is.na(Wall$ph048d1) & Wall$ph048d1 == "Not selected",0, NA)); table(Wall$ph048d1, Wall$walking_difficulty, useNA = "always")
Wall$stairs_difficulty <- ifelse(!is.na(Wall$ph048d5) & Wall$ph048d5 == "Selected", 1, ifelse(!is.na(Wall$ph048d5) & Wall$ph048d5 == "Not selected",0, NA)); table(Wall$ph048d5, Wall$stairs_difficulty, useNA = "always")

Wall$Slowness <- ifelse((!is.na(Wall$walking_difficulty) & Wall$walking_difficulty ==1) | (!is.na(Wall$stairs_difficulty) & Wall$stairs_difficulty == 1), 1, ifelse(is.na(Wall$walking_difficulty)|is.na(Wall$stairs_difficulty), NA, 0))
table(Wall$Slowness, useNA = "always")

The low activity criterion was assessed by the question: "How often do you engage in activities that require a low or moderate level of energy such as gardening, cleaning the car, or doing a walk?".
This variable was kept ordinal: 1 = "More than once a week"; 2= "Once a week"; 3 = One to three times a month" and 4 = "Hardly ever or never".
Wall$Low_activity <- as.numeric(factor(Wall$br016_, levels = c("More than once a week", "Once a week", "One to three times a month", "Hardly ever, or never"))); table(Wall$Low_activity, useNA = "always")

Wall$CompleteCase <- !is.na(Wall$Fatigue) & !is.na(Wall$Loss_of_appetite) & !is.na(Wall$Gripstrength) & !is.na(Wall$Slowness) & !is.na(Wall$Low_activity)
table(Wall$CompleteCase, Wall$Wave)

################################

3. Missingness patterns
################################
vis_miss(Wall %>% select(Fatigue, Loss_of_appetite, Gripstrength, Slowness, Low_activity), warn_large_data = F)
# high rate of missingness with clusters. Check by waves:
gg_miss_fct(Wall %>% select(Wave, country, Fatigue, Loss_of_appetite, Gripstrength, Slowness, Low_activity), fct = Wave)

```
# ~80% missing: gripstrength (W5), low activity and fatigue (W7).

# High rate of incomplete cases in Wave 5 and Wave 7. Check for details:
table(Wall$Wave[Wall$Wave %in% c("W5", "W7")], Wall$Fatigue[Wall$Wave %in% c("W5", "W7")],
useNA = "always")
table(Wall$Wave[Wall$Wave %in% c("W5", "W7")], Wall$Loss_of_appetite[Wall$Wave %in%
c("W5", "W7")], useNA = "always")
table(Wall$Wave[Wall$Wave %in% c("W5", "W7")], is.na(Wall$Gripstrength[Wall$Wave %in%
c("W5", "W7")]), useNA = "always")
table(Wall$Wave[Wall$Wave %in% c("W5", "W7")], Wall$Slowness[Wall$Wave %in% c("W5",
"W7")], useNA = "always")
table(Wall$Wave[Wall$Wave %in% c("W5", "W7")], Wall$Low_activity[Wall$Wave %in% c("W5",
"W7")], useNA = "always")
# in Wave 5, high rate of missing gripstrength data.
# In Wave 7, high rate of missing data on Fatigue, Loss of appetite, and low activity.
# These missingness patterns are considered to reflect survey design or practice changes across
waves.
# Removing complete waves will result in missingness at random, no impact on analysis results.
# These waves are omitted from further Frailty Instrument analyses
Wall2 <- Wall %>% filter(Wave %in% c("W5", "W7") == F)

vis_miss(Wall2 %>% select(Fatigue, Loss_of_appetite, Gripstrength, Slowness, Low_activity),
warn_large_data = F)
# missingness is mostly affecting gripstrength data, and many cases have combined missingness
across all frailty instrument domains.
gg_miss_upset(Wall2 %>% select(Fatigue, Loss_of_appetite, Gripstrength, Slowness, Low_activity))
# In Waves 1-2-4-6-8, the most typical missingness related to frailty instrument was the isolated lack
of gripstrength data (N=15,557), followed by complete data lack on the five dimensions of frailty
instrument (N=10,129), and lack of data on loss of appetite, fatigue, gripstrength (N=4415). Other
combinations of data missingness were rare (<= 500 of the 244,834 observations)).

# Is missingness in Waves 1-2-4-6-8 at random?
gg_miss_var(Wall2 %>% select(Agebands, Fatigue, Loss_of_appetite, Gripstrength, Slowness,
Low_activity) , facet = Agebands, show_pct = T)
gg_miss_fct(Wall2 %>% select(Agebands, Fatigue, Loss_of_appetite, Gripstrength, Slowness,
Low_activity), fct = Agebands)
# Missingness of all instrument components is associated with higher ages - where frailty is most
prevalent.

gg_miss_fct(Wall2 %>% select(Fatigue, Loss_of_appetite, Gripstrength, Slowness, Low_activity), fct =
Low_activity)
# Missingness increases with lower activity (visual check)

Wall2 %>% select(Fatigue, Loss_of_appetite, Gripstrength, Slowness, Low_activity) %>%
  group_by(Low_activity) %>% miss_var_summary() %>% arrange(variable, Low_activity) %>%
  data.frame() %>% print()
  # Low activity: the higher the score, the more missing data in other dimensions.

Wall2 %>% select(Fatigue, Loss_of_appetite, Gripstrength, Slowness, Low_activity) %>%
  group_by(Fatigue) %>% miss_var_summary() %>% arrange(variable, Fatigue) %>%
  data.frame() %>% print()
```

```
  # Fatigue: more missing data in gripstrength; possibly also in other dimensions.

Wall2 %>% select(Fatigue, Loss_of_appetite, Gripstrength, Slowness, Low_activity) %>%
  group_by(Loss_of_appetite) %>% miss_var_summary() %>% arrange(variable, Loss_of_appetite)
%>%
  data.frame() %>% print()
  # Loss of appetite: more missing data in gripstrength; possibly also in other dimensions.

Wall2 %>% select(Fatigue, Loss_of_appetite, Gripstrength, Slowness, Low_activity) %>%
  group_by(Slowness) %>% miss_var_summary() %>% arrange(variable, Slowness) %>%
  data.frame() %>% print()
  # Loss of appetite: more missing data in gripstrength and also in other dimensions.

Wall2 %>% select(Fatigue, Loss_of_appetite, Gripstrength, Slowness, Low_activity) %>%
  group_by(is.na(Gripstrength)) %>% miss_var_summary() %>% arrange(variable) %>%
  data.frame() %>% print() # Loss of gripstrength data: 33% to 48% missingness in other frailty
instrument data dimensions.

# Missingness of frailty instrument components are not at random:
# Missingness increases with age and apparently also with frailty signals in other components.
# Missing data in one frailty instrument dimension predisposes for missingness in other dimensions
as well.

# Hence, listwise deletion of surveys with missing data (complete case analysis) may be biased
# and is conducted only as sensitivity analysis.
# The primary analysis will include all surveys of the included waves using multiple imputation.
################################

#  4. Complete case analysis
################################
# sensitivity analysis using only complete data for assessing frailty, as it was done in Romero-Ortuno
et al. BMC Geriatrics 2010, 10:57

Wall_c <- Wall2 %>% filter(CompleteCase == T) %>%
  select(mergeid, Wave, country, Age, Agebands, gender, int_year, Fatigue, Loss_of_appetite,
       Gripstrength, Slowness, Low_activity, GDP_cap_k, GDP_cap_PPP_k)
summary(Wall_c)

# merge highest agebands
levels(Wall_c$Agebands) <- c(levels(Wall_c$Agebands), "85 or higher")
Wall_c$Agebands[Wall_c$Agebands %in% c("85 to 89", "90 or higher")] <- "85 or higher"


Wall_c$Wave <- factor(Wall_c$Wave)
Wall_c$gender <- as.character(factor(Wall_c$gender, levels = c("Male", "Female")))
Wall_c$int_year <- factor(Wall_c$int_year)
Wall_c$Fatigue <- as.numeric(as.character(Wall_c$Fatigue))
Wall_c$Loss_of_appetite <- as.numeric(as.character(Wall_c$Loss_of_appetite))
Wall_c$Slowness <- as.numeric(as.character(Wall_c$Slowness))
Wall_c$Low_activity <- as.numeric(as.character(Wall_c$Low_activity))
Wall_c$GDP_cap_k <- as.numeric(Wall_c$GDP_cap_k)
```

```
Wall_c$GDP_cap_PPP_k <- as.numeric(Wall_c$GDP_cap_PPP_k)
summary(Wall_c)

### calculate the frailty instrument outputs
Wall_c$DFactorScore <- ifelse(is.na(Wall_c$gender), NA, ifelse(
  Wall_c$gender == "Male", ((2.280336*Wall_c$Fatigue - 0.592393)* 0.3762  + (4.058274 *
Wall_c$Loss_of_appetite - 0.263501)*0.3130 + (0.092326*Wall_c$Gripstrength-3.986646)*(-0.4653)
+ (3.098226*Wall_c$Slowness - 0.365971)*0.6146 + (1.005942*Wall_c$Low_activity -
1.571803)*0.4680), ifelse(
    Wall_c$gender == "Female", ((2.077707*Wall_c$Fatigue - 0.757295)* 0.4088  + (3.341539 *
Wall_c$Loss_of_appetite - 0.332289)*0.3325 + (0.132827*Wall_c$Gripstrength-3.534515)*(-0.4910)
+ (2.627085*Wall_c$Slowness - 0.461808)*0.6012 + (0.918866*Wall_c$Low_activity -
1.523633)*0.4818), NA)))
# the calculation was corrected for males regarding grip strength multiplier:
# in the text it was -0.4910 but "minus" sign was omitted in the final calculation formula on page 7 of
the paper.
# However, checking the online calculator (electronic suppl material to the paper) it became clear
that
# a higher gripstrength should decrease the DFScore, not increase it. Therefore, the minus correction
# has been fixed above for gripstrength in males.

hist(Wall_c$DFactorScore) # the distribution is resembling the distribution on Figure 2 of the original
publication
Wall_c$Frailty_Instrument_Category <- ifelse(is.na(Wall_c$DFactorScore)|is.na(Wall_c$gender), NA,
ifelse(
  Wall_c$gender == "Male" & Wall_c$DFactorScore < 1.211878526, "NON-FRAIL", ifelse(
    Wall_c$gender == "Male" & Wall_c$DFactorScore < 3.0052612772, "PRE-FRAIL", ifelse(
      Wall_c$gender == "Male" & Wall_c$DFactorScore >= 3.0052612772, "FRAIL", ifelse(
        Wall_c$gender == "Female" & Wall_c$DFactorScore <  0.3151361243, "NON-FRAIL", ifelse(
          Wall_c$gender == "Female" & Wall_c$DFactorScore < 2.1301121973, "PRE-FRAIL", ifelse(
            Wall_c$gender == "Female" & Wall_c$DFactorScore >= 2.1301121973, "FRAIL", "???")))))))
table(Wall_c$Frailty_Instrument_Category, Wall_c$gender)
Wall_c$Frailty_Instrument_Category <- factor(Wall_c$Frailty_Instrument_Category, levels = c("NON-
FRAIL", "PRE-FRAIL", "FRAIL"))

library(ggplot2)
ggplot(Wall_c, aes(x=DFactorScore, color = country)) + stat_ecdf(geom = "step") +
geom_vline(xintercept = 2.1301121973) + geom_vline(xintercept = 3.0052612772) +
facet_grid(Agebands ~ gender)
#Check the cumulative distribution of DFactorScore by age bands and sexes by country.
# The vertical lines are frailty thresholds for females and males, respectively.

Summary_c <- Wall_c %>% group_by(country, gender, Agebands) %>% summarize(
  N_total = n(), N_frail =  sum(Frailty_Instrument_Category == "FRAIL"),
  Prop_frail = N_frail / N_total, GDP_cap_k = mean(GDP_cap_k), GDP_cap_PPP_k =
mean(GDP_cap_PPP_k)) %>% ungroup()
# add exact confidence intervals to the proportions
Summary_c$Prop_frail_CI_lower <- 99
Summary_c$Prop_frail_CI_upper <- 99
library(PropCIs)
for(i in 1:length(Summary_c$Prop_frail)){
```

```
  Summary_c$Prop_frail_CI_lower[i] <- exactci(Summary_c$N_frail[i],Summary_c$N_total[i],
0.95)[[1]][1]
  Summary_c$Prop_frail_CI_upper[i] <- exactci(Summary_c$N_frail[i],Summary_c$N_total[i],
0.95)[[1]][2]}

# Visual check of observed data
ggplot(Summary_c) + geom_point(aes(x=GDP_cap_k, y=Prop_frail, color = Agebands, size = N_total))
+
  facet_grid(gender ~ Agebands) + theme_clean() + theme(axis.text.x = element_text(angle = 45))
ggplot(Summary_c) + geom_point(aes(x=GDP_cap_PPP_k, y=Prop_frail, color = Agebands, size =
N_total)) +
  facet_grid(gender ~ Agebands) + theme_clean() + theme(axis.text.x = element_text(angle = 45))

### Mixed model regression to set up predictive function by age, gender and GDP per capita PPP
# two observed interactions at visual check: gender difference is age dependent; GDP effect is age
dependent.
# fixed factors (predictors) include ageband, gender; and GDP_cap
# random factors to reflect lack of independence in the data: same country:same mergeid
# random intercept by mergeid: to control for repeated survey in many participants in different
waves
# random intercept by country: to control for other country effects beyond GDP per capita
# age is not numeric but categorical as non-linear and non loglinear associations are possible

Wall_c$Frail <- Wall_c$Frailty_Instrument_Category == "FRAIL"
table(Wall_c$Frailty_Instrument_Category, Wall_c$Frail, useNA = "always")

model_c <- glmer(Frail ~ gender * Agebands + Agebands * GDP_cap_k +
             (1|country) + (1|country:mergeid), family = binomial(link = "logit"), data = Wall_c, nAGQ =
0)
summary(model_c) # this is the complete case model

# predictions using this model for extrapolation to other European countries
# visual check of predictions
Summary_c$Predict_prob <- predict(model_c, newdata = Summary_c, re.form = NA, type =
"response")
ggplot(Summary_c) + geom_point(aes(x=GDP_cap_k, y=Prop_frail, color = Agebands, size = N_total))
+
  geom_line(aes(x=GDP_cap_k, y=Predict_prob), linetype = "dashed", color = "black", size = 1) +
  facet_grid(gender ~ Agebands) + theme_clean() + theme(axis.text.x = element_text(angle = 45))

model_c_ppp <- glmer(Frail ~ gender * Agebands + Agebands * GDP_cap_PPP_k +
             (1|country) + (1|country:mergeid), family = binomial(link = "logit"), data = Wall_c, nAGQ =
0)
summary(model_c_ppp, digits = 4) # this is the complete case model

# predictions using this model for extrapolation to other European countries
# visual check of predictions
Summary_c$Predict_prob_ppp <- predict(model_c_ppp, newdata = Summary_c, re.form = NA, type
= "response")
ggplot(Summary_c) + geom_point(aes(x=GDP_cap_PPP_k, y=Prop_frail, color = Agebands, size =
N_total)) +
```

```
  geom_line(aes(x=GDP_cap_PPP_k, y=Predict_prob_ppp), linetype = "dashed", color = "black", size = 1) +
  facet_grid(gender ~ Agebands) + theme_clean() + theme(axis.text.x = element_text(angle = 45))

################################

#  5. Multiple imputation and analysis of the full sample
################################
# Missingness within waves is not at random; a complete case analysis would systematically undersample patients
# with higher risk for frailty. Data imputations need to take into account ageband, gender,
# data dimensions of frailty instrument, and some auxiliary variables proposed by SHARE for hot deck imputations:
# an indicator for people living with a spouse/partner,
# five groups for years of education, and two groups for self-reported good/bad health.

# Collect data on education years: Waves 2-8, dn041_ How many years have you been in full time education?
# + Wave 1: isced1997y_r, Respondent: years of education derived from ISCED-97, -7	Not yet coded (temporary); -2	Refusal; -1	Don't know; 0	None; 95	Still in school; 97	Other
EDU <- rbind(
  read.dta("sharew1_rel8-0-0_gv_isced.dta", missing.type = T) %>% as.data.frame() %>% select(mergeid, isced1997y_r) %>% mutate(Wave = "W1", isced1997y_r = ifelse(isced1997y_r<0|isced1997y_r>90, NA, isced1997y_r)) %>% rename(Edu_y = isced1997y_r),
  read.dta("sharew2_rel8-0-0_dn.dta", missing.type = T) %>% as.data.frame() %>% select(mergeid, dn041_) %>% mutate(Wave = "W2", dn041_ = ifelse(dn041_<0, NA, dn041_)) %>% rename(Edu_y = dn041_),
  read.dta("sharew4_rel8-0-0_dn.dta", missing.type = T) %>% as.data.frame() %>% select(mergeid, dn041_) %>% mutate(Wave = "W4", dn041_ = ifelse(dn041_<0, NA, dn041_)) %>% rename(Edu_y = dn041_),
  read.dta("sharew5_rel8-0-0_dn.dta", missing.type = T) %>% as.data.frame() %>% select(mergeid, dn041_) %>% mutate(Wave = "W5", dn041_ = ifelse(dn041_<0, NA, dn041_)) %>% rename(Edu_y = dn041_),
  read.dta("sharew6_rel8-0-0_dn.dta", missing.type = T) %>% as.data.frame() %>% select(mergeid, dn041_) %>% mutate(Wave = "W6", dn041_ = ifelse(dn041_<0, NA, dn041_)) %>% rename(Edu_y = dn041_),
  read.dta("sharew7_rel8-0-0_dn.dta", missing.type = T) %>% as.data.frame() %>% select(mergeid, dn041_) %>% mutate(Wave = "W7", dn041_ = ifelse(dn041_<0, NA, dn041_)) %>% rename(Edu_y = dn041_),
  read.dta("sharew8_rel8-0-0_dn.dta", missing.type = T) %>% as.data.frame() %>% select(mergeid, dn041_) %>% mutate(Wave = "W8", dn041_ = ifelse(dn041_<0, NA, dn041_)) %>% rename(Edu_y = dn041_))
gg_miss_fct(EDU %>% select(Wave, Edu_y), fct = Wave)
# missing education years data especially in later waves. Imputed using observations across all waves, assuming no changes in full time education years in the 50+ years of age, when data is missing:
EDU <- EDU %>% mutate(Edu_y = ifelse(Edu_y <0 |Edu_y>90, NA, Edu_y)) %>% group_by(mergeid) %>% mutate(Edu_y_i = max(Edu_y, na.rm = T)) %>% ungroup() %>% select(mergeid, Edu_y_i) %>% distinct()
# generate EDU categories
```

```
hist(EDU$Edu_y_i)
table(as.numeric(EDU$Edu_y_i), useNA = "always")
EDU$Edu_y_i[EDU$Edu_y_i < 0] <- NA
table(as.numeric(EDU$Edu_y_i), useNA = "always")
EDU$Edu_cat <- ifelse(is.na(EDU$Edu_y_i), "Missing", ntile(EDU$Edu_y_i, 4)) %>% factor()
table(EDU$Edu_cat, useNA = "always")
EDU_table2 <- EDU %>% select (mergeid, Edu_y_i, Edu_cat)
EDU <- EDU %>% select (mergeid, Edu_cat)
EDU$Edu_cat[is.na(EDU$Edu_cat)] <- "Missing"

# Collect data on living with a spouse/partner: dn014_ Marital status: What is your marital status?
# 1.     Married and living together with spouse; 2.     Registered partnership; 3.     Married,
living separated from spouse; 4.     Never married; 5.     Divorced; 6.     Widowed
SPP <- rbind(
  read.dta("sharew1_rel8-0-0_dn.dta", missing.type = T) %>% as.data.frame() %>% select(mergeid,
dn014_) %>% mutate(Wave = "W1"),
  read.dta("sharew2_rel8-0-0_dn.dta", missing.type = T) %>% as.data.frame() %>% select(mergeid,
dn014_) %>% mutate(Wave = "W2"),
  read.dta("sharew4_rel8-0-0_dn.dta", missing.type = T) %>% as.data.frame() %>% select(mergeid,
dn014_) %>% mutate(Wave = "W4"),
  read.dta("sharew6_rel8-0-0_dn.dta", missing.type = T) %>% as.data.frame() %>% select(mergeid,
dn014_) %>% mutate(Wave = "W6"),
  read.dta("sharew8_rel8-0-0_dn.dta", missing.type = T) %>% as.data.frame() %>% select(mergeid,
dn014_) %>% mutate(Wave = "W8"))
gg_miss_fct(SPP %>% select(Wave, dn014_), fct = Wave)
SPP$MaritalStatus <- ifelse(is.na(SPP$dn014_)|SPP$dn014_ %in% c("Refusal", "Don't know"),
"Missing", ifelse(SPP$dn014_ %in% c("Married and living together with spouse", "Registered
partnership"), "Together", "Alone")) %>% factor(levels = c("Alone", "Together", "Missing"))
SPP <- SPP %>% select(mergeid, Wave, MaritalStatus)
table(SPP$Wave, SPP$MaritalStatus, useNA = "always")

# Collect data on self-reported good/bad health: ph003_ Would you say your health is...1. Excellent
2. Very good 3. Good 4. Fair 5. Poor
SRH <- rbind(
  read.dta("sharew1_rel8-0-0_ph.dta", missing.type = T) %>% as.data.frame() %>% select(mergeid,
ph003_) %>% mutate(Wave = "W1"),
  read.dta("sharew2_rel8-0-0_ph.dta", missing.type = T) %>% as.data.frame() %>% select(mergeid,
ph003_) %>% mutate(Wave = "W2"),
  read.dta("sharew4_rel8-0-0_ph.dta", missing.type = T) %>% as.data.frame() %>% select(mergeid,
ph003_) %>% mutate(Wave = "W4"),
  read.dta("sharew6_rel8-0-0_ph.dta", missing.type = T) %>% as.data.frame() %>% select(mergeid,
ph003_) %>% mutate(Wave = "W6"),
  read.dta("sharew8_rel8-0-0_ph.dta", missing.type = T) %>% as.data.frame() %>% select(mergeid,
ph003_) %>% mutate(Wave = "W8"))
gg_miss_fct(SRH %>% select(Wave, ph003_), fct = Wave)
SRH$Health <- ifelse(is.na(SRH$ph003_)|SRH$ph003_ %in% c("Refusal", "Don't know"), "Missing",
ifelse(SRH$ph003_ %in% c("Excellent", "Very good", "Good"), "Good", "Not good")) %>%
factor(levels = c("Good", "Not good", "Missing"))
table(SRH$ph003_, SRH$Health, useNA = "always")
table(SRH$Wave, SRH$Health, useNA = "always")
SRH <- SRH %>% select(mergeid, Wave, Health)
```

```
###### combine Wall2 with auxiliary data for missing data imputations
Wall_i <- Wall2 %>% select(mergeid, Wave, country, Agebands, gender, Fatigue, Loss_of_appetite,
Gripstrength, Slowness, Low_activity) %>% left_join(EDU) %>% left_join(SPP) %>% left_join(SRH)
summary(Wall_i)
Wall_i$Edu_cat[is.na(Wall_i$Edu_cat)] <- "Missing"
Wall_i$MaritalStatus[is.na(Wall_i$MaritalStatus)] <- "Missing"
Wall_i$Health[is.na(Wall_i$Health)] <- "Missing"
Wall_i$gender <- factor(Wall_i$gender, levels = c("Male", "Female"))

###### check extent of missingness in frailty instrument dimensions
Wall_i$FI_missingdim <- is.na(Wall_i$Fatigue) + is.na(Wall_i$Loss_of_appetite) +
is.na(Wall_i$Gripstrength) + is.na(Wall_i$Slowness) + is.na(Wall_i$Low_activity)
table(Wall_i$Edu_cat, Wall_i$FI_missingdim, useNA = "always")

# adjust parameter types before imputation
Wall_i$Fatigue <- factor(Wall_i$Fatigue); table(Wall_i$Fatigue, useNA = "always")
Wall_i$Loss_of_appetite <- factor(Wall_i$Loss_of_appetite); table(Wall_i$Loss_of_appetite, useNA
= "always")
Wall_i$Gripstrength <- as.numeric(Wall_i$Gripstrength); summary(Wall_i$Gripstrength)
Wall_i$Slowness <- factor(Wall_i$Slowness); table(Wall_i$Slowness, useNA = "always")
Wall_i$Low_activity <- factor(Wall_i$Low_activity); table(Wall_i$Low_activity, useNA = "always")

library(mice)
init = mice(Wall_i, maxit=0)
meth = init$method
predM = init$predictorMatrix

predM[, c("mergeid", "FI_missingdim")]=0
# remove these variable as predictors but these still will be imputed.

meth[c("mergeid", "Wave", "country", "Edu_cat", "MaritalStatus", "Health", "FI_missingdim")]=""
# Columns that need not be imputed have the empty method "".

# Now let specify the methods for imputing the missing values.
meth[c("Gripstrength")]="pmm"
meth[c("Fatigue", "Slowness", "Loss_of_appetite")]="logreg"
meth[c("Low_activity")]="pmm"

# Now it is time to run the multiple (m=10) imputation.
set.seed(2023)
imputed = mice(Wall_i, method=meth, predictorMatrix=predM, m=10)

# check imputations
imp_long <- complete(imputed, action = "long", include = T)
table(imp_long$.imp, imp_long$Fatigue, useNA = "always")
# fatigue imputations are complete for each imputation rounds.
table(imp_long$.imp, imp_long$Loss_of_appetite, useNA = "always")
# loss of appetite imputations are complete for each imputation rounds.
table(imp_long$.imp, is.na(imp_long$Gripstrength), useNA = "always")
# gripstrength imputations are complete for each imputation rounds.
```

```
table(imp_long$.imp, imp_long$Slowness, useNA = "always")
# slowness imputations are complete for each imputation rounds.
table(imp_long$.imp, imp_long$Low_activity, useNA = "always")
# low activity imputations are complete for each imputation rounds.

# Inspect the convergence of the algorithm (iterations tend to the same imputed value ranges):
plot(imputed) # convergence achieved after 2-3 iterations

# Inspect the distribution of original and imputed data. Are the imputed points plausible values?
densityplot(imputed)
# the slight shift in gripstrength to lower values is consistent with
# the observed non-random missingness patterns (missingness of gripstrength data is
# more frequently observed at higher ages and in cases with lower activity levels).

# check categorical parameters (observed vs. imputed):
miss_Fat <- imp_long %>% filter(is.na(Fatigue)) %>% select(.id) %>% pull()
table(imp_long$.id %in% miss_Fat, imp_long$Fatigue, imp_long$.imp, useNA = "always")
# higher rate of fatigue is appearing in the inputed data, this is plausible
rm(miss_Fat)
miss_App <- imp_long %>% filter(is.na(Loss_of_appetite)) %>% select(.id) %>% pull()
table(imp_long$.id %in% miss_App, imp_long$Loss_of_appetite, imp_long$.imp, useNA = "always")
# slightly higher rate of lost appetite is appearing in the inputed data, this is plausible
rm(miss_App)
miss_Slo <- imp_long %>% filter(is.na(Slowness)) %>% select(.id) %>% pull()
table(imp_long$.id %in% miss_Slo, imp_long$Slowness, imp_long$.imp, useNA = "always")
# higher rate of slowness is appearing in the inputed data, this is plausible
rm(miss_Slo)
miss_Loa <- imp_long %>% filter(is.na(Low_activity)) %>% select(.id) %>% pull()
table(imp_long$.id %in% miss_Loa, imp_long$Low_activity, imp_long$.imp, useNA = "always")
# a trend for lower activity is appearing in the inputed data, this is plausible (non-random
missingness)
rm(miss_Loa)
# imputed patterns seem to be OK, plausible values.

# continue with frailty calculation and modelling on the imputed data:

Wall_i_plus <- Wall2 %>% select(mergeid, Wave, int_year, CompleteCase, Year, GDP_cap_k,
GDP_cap_PPP_k) # these are the paarameters to add to the imputed dataset
imp_long <- imp_long %>% mutate(
  Wave = factor(Wave),
  gender = as.character(factor(gender, levels = c("Male", "Female"))),
  Fatigue = as.numeric(as.character(Fatigue)),
  Loss_of_appetite = as.numeric(as.character(Loss_of_appetite)),
  Slowness = as.numeric(as.character(Slowness)),
  Low_activity = as.numeric(as.character(Low_activity))) %>% left_join(Wall_i_plus) %>%
mutate(DFactorScore = ifelse(is.na(gender), NA, ifelse(
    gender == "Male", ((2.280336*Fatigue - 0.592393)* 0.3762  + (4.058274 * Loss_of_appetite -
0.263501)*0.3130 + (0.092326*Gripstrength-3.986646)*(-0.4653) + (3.098226*Slowness -
0.365971)*0.6146 + (1.005942*Low_activity - 1.571803)*0.4680), ifelse(
```

```
    gender == "Female", ((2.077707*Fatigue - 0.757295)* 0.4088  + (3.341539 * Loss_of_appetite - 
0.332289)*0.3325 + (0.132827*Gripstrength-3.534515)*(-0.4910) + (2.627085*Slowness - 
0.461808)*0.6012 + (0.918866*Low_activity - 1.523633)*0.4818), NA))))

hist(imp_long$DFactorScore) # very similar distribution to Figure 2 of the original publication

imp_long <- imp_long %>% rowwise() %>% mutate(Frailty_Instrument_Category = 
ifelse(is.na(DFactorScore)|is.na(gender), NA, ifelse(
  gender == "Male" & DFactorScore < 1.211878526, "NON-FRAIL", ifelse(
    gender == "Male" & DFactorScore < 3.0052612772, "PRE-FRAIL", ifelse(
      gender == "Male" & DFactorScore >= 3.0052612772, "FRAIL", ifelse(
        gender == "Female" & DFactorScore <  0.3151361243, "NON-FRAIL", ifelse(
          gender == "Female" & DFactorScore < 2.1301121973, "PRE-FRAIL", ifelse(
            gender == "Female" & DFactorScore >= 2.1301121973, "FRAIL", "???")))))))) %>% ungroup()
imp_long$Frailty_Instrument_Category <- factor(imp_long$Frailty_Instrument_Category, levels = 
c("NON-FRAIL", "PRE-FRAIL", "FRAIL"))
table(imp_long$Frailty_Instrument_Category, imp_long$gender, imp_long$.imp, useNA = "always")

imp_long$GDP_cap_k <- as.numeric(imp_long$GDP_cap_k)
imp_long$GDP_cap_PPP_k <- as.numeric(imp_long$GDP_cap_PPP_k)

# densityplot(as.mids(imp_long))
# distribution of numeric parameters in complete (blue) and imputed(purple) cases - visual summary
# imputed patterns seem to be OK, plausible values (trends for higher frailty in imputed data was 
# expected as missingness was higher at high ages)


# merge highest agebands
levels(imp_long$Agebands) <- c(levels(imp_long$Agebands), "85 or higher")
imp_long$Agebands[imp_long$Agebands %in% c("85 to 89", "90 or higher")] <- "85 or higher"


library(ggplot2)
ggplot(imp_long, aes(x=DFactorScore, color = country)) + stat_ecdf(geom = "step") + 
geom_vline(xintercept = 2.1301121973) + geom_vline(xintercept = 3.0052612772) + 
facet_grid(Agebands ~ gender)
#Check the cumulative distribution of DFactorScore by age bands and sexes by country.
# The vertical lines are the frailty thresholds for females and males, respectively.

Summary_i <- imp_long %>% filter(.imp > 0) %>%
  mutate(imp = .imp) %>% group_by(country, gender, Agebands) %>%
  summarize(N_total = sum(!is.na(Frailty_Instrument_Category))/10,
         N_frail =  round(sum(Frailty_Instrument_Category == "FRAIL", na.rm = T)/10, 0),
         Prop_frail = N_frail / N_total,
         GDP_cap_k = mean(GDP_cap_k),
         GDP_cap_PPP_k = mean(GDP_cap_PPP_k)) %>%
  ungroup()
summary(Summary_i$Prop_frail) # average of 10 multiple imputation sets

# add exact confidence intervals to these proportions
Summary_i$Prop_frail_CI_lower <- 99; Summary_i$Prop_frail_CI_upper <- 99
```

```
for(i in 1:length(Summary_i$Prop_frail)){
  Summary_i$Prop_frail_CI_lower[i] <- exactci(Summary_i$N_frail[i],Summary_i$N_total[i],
0.95)[[1]][1]
  Summary_i$Prop_frail_CI_upper[i] <- exactci(Summary_i$N_frail[i],Summary_i$N_total[i],
0.95)[[1]][2]}

# Visual check of observed data
ggplot(Summary_i) + geom_point(aes(x=GDP_cap_k, y=Prop_frail, color = Agebands, size = N_total))
+
  facet_grid(gender ~ Agebands) + theme_clean() + theme(axis.text.x = element_text(angle = 45))

### Mixed model regression to set up predictive function by age, gender and GDP per capita PPP
# two observed interactions at visual check: gender difference is age dependent; GDP effect is age
dependent.
# fixed factors (predictors) include ageband, gender; and GDP_cap
# random factors to reflect lack of independence in the data: same country:same mergeid
# random intercept by mergeid: to control for repeated survey in many participants in different
waves
# random intercept by country: to control for other country effects beyond GDP per capita
# age is not numeric but categorical as non-linear and non-loglinear associations are possible

imp_long$Frail <- imp_long$Frailty_Instrument_Category == "FRAIL"
imp_data <- as.mids(imp_long)

model_i <- with(imp_data,
             glmer(Frail ~ gender * Agebands + Agebands * GDP_cap_k +
               (1|country) + (1|country:mergeid), family = binomial(link = "logit"), nAGQ = 0))

# The pool() function combines the estimates from m repeated complete data analyses.
# The typical sequence of steps to perform a multiple imputation analysis is:
# Impute the missing data by the mice() function, resulting in a multiple imputed data set (class
mids);
# Fit the model of interest (scientific model) on each imputed data set by the with() function,
# resulting an object of class mira; Pool the estimates from each model into a single set of estimates
# and standard errors, resulting in an object of class mipo; Optionally, compare pooled estimates
# from different scientific models by the D1() or D3() functions. A common error is to reverse steps 2
and 3,
# i.e., to pool the multiply-imputed data instead of the estimates. Doing so may severely bias the
estimates
# of scientific interest and yield incorrect statistical intervals and p-values.

model_i_pooled <- summary(pool(model_i), dfcom = model_i$analyses[[1]]@devcomp$dims[4], rule
= "rubin1987")
# predict from model (mira class)
model_i_predict <- model_i$analyses[[1]] # a model to be adjusted with pooled parameter
estimates
model_i_predict@beta <- model_i_pooled$estimate # model for prediction
imp_long$predict_prob <- predict(model_i_predict, newdata = imp_long, re.form = NA, type =
"response")
# predictions using this model for extrapolation to other European countries:
# visual check of predictions
```

```
Summary_i$Predict_prob <- predict(model_i_predict, newdata = Summary_i, re.form = NA, type =
"response")
ggplot(Summary_i) + geom_point(aes(x=GDP_cap_k, y=Prop_frail, color = Agebands, size = N_total))
+
  geom_line(aes(x=GDP_cap_k, y=Predict_prob), linetype = "dashed", color = "black", size = 1) +
  facet_grid(gender ~ Agebands) + theme_clean() + theme(axis.text.x = element_text(angle = 45))

# PPP analysis
model_i_ppp <- with(imp_data,
            glmer(Frail ~ gender * Agebands + Agebands * GDP_cap_PPP_k +
                  (1|country) + (1|country:mergeid), family = binomial(link = "logit"), nAGQ = 0))
model_i_ppp_pooled <- summary(pool(model_i_ppp), dfcom =
model_i_ppp$analyses[[1]]@devcomp$dims[4], rule = "rubin1987")
# predict from model (mira class)
model_i_predict_ppp <- model_i_ppp$analyses[[1]] # a model to be adjusted with pooled parameter
estimates
model_i_predict_ppp@beta <- model_i_ppp_pooled$estimate # model for prediction
imp_long$predict_prob_ppp <- predict(model_i_predict_ppp, newdata = imp_long, re.form = NA,
type = "response")
# predictions using this model for extrapolation to other European countries:
# visual check of predictions
Summary_i$Predict_prob_ppp <- predict(model_i_predict_ppp, newdata = Summary_i, re.form =
NA, type = "response")
ggplot(Summary_i) + geom_point(aes(x=GDP_cap_PPP_k, y=Prop_frail, color = Agebands, size =
N_total)) +
  geom_line(aes(x=GDP_cap_PPP_k, y=Predict_prob_ppp), linetype = "dashed", color = "black", size =
1) +
  facet_grid(gender ~ Agebands) + theme_clean() + theme(axis.text.x = element_text(angle = 45))

################################

#  6. Derive data for the Frailty Atlas
################################
# The comprehensive list of frailty estimates by country, gender, and age bands with 95% confidence
intervals
# will be provided in tabular format as part of the SHARE Frailty Atlas (online suppl. materials)

Country_list <- data.frame(crossing(country = GDP$country, Agebands = Wall_c$Agebands,
                          gender = c("Male", "Female"))) %>%
  filter(Agebands %in% c("85 to 89", "90 or higher") == F) %>%
  droplevels()

Atlas_Instr_obs_cc <-  Summary_c %>%
  select(country, gender, Agebands, Prop_frail, Prop_frail_CI_lower, Prop_frail_CI_upper) %>%
  right_join(Country_list) %>% select(country, gender, Agebands, Prop_frail,
  Prop_frail_CI_lower, Prop_frail_CI_upper)  %>%
  mutate(datatype = "Observed values", Missingness = "Complete cases",
       Frailty_method = "SHARE Frailty Instrument")

Atlas_Instr_obs_imp <-  Summary_i %>%
  select(country, gender, Agebands, Prop_frail, Prop_frail_CI_lower, Prop_frail_CI_upper) %>%
```

```
  right_join(Country_list) %>% mutate(datatype = "Observed values", Missingness = "Multiple
imputation",
        Frailty_method = "SHARE Frailty Instrument") %>%select(names(Atlas_Instr_obs_cc))

Atlas_Instr_predicted_cc_GDP_kEUR <- Country_list %>%
  left_join(GDP %>% select(country, GDP_cap_k)) %>%
  mutate(GDP_cap_k = as.numeric(GDP_cap_k),
        datatype = "Prediction_GDP_kEUR",
        Prop_frail_CI_lower = NA, Prop_frail_CI_upper = NA,
        Missingness = "Complete cases",
        Frailty_method = "SHARE Frailty Instrument")
Atlas_Instr_predicted_cc_GDP_kEUR$Prop_frail <- predict(
  model_c, newdata = Atlas_Instr_predicted_cc_GDP_kEUR, re.form = NA, type = "response")
Atlas_Instr_predicted_cc_GDP_kEUR <- Atlas_Instr_predicted_cc_GDP_kEUR %>%
select(names(Atlas_Instr_obs_cc))

Atlas_Instr_predicted_cc_GDP_PPP <- Country_list %>%
  left_join(GDP_PPP %>% select(country, GDP_cap_PPP_k)) %>%
  mutate(GDP_cap_PPP_k = as.numeric(GDP_cap_PPP_k),
        datatype = "Prediction_GDP_PPP",
        Prop_frail_CI_lower = NA, Prop_frail_CI_upper = NA,
        Missingness = "Complete cases",
        Frailty_method = "SHARE Frailty Instrument")
Atlas_Instr_predicted_cc_GDP_PPP$Prop_frail <- predict(
  model_c_ppp, newdata = Atlas_Instr_predicted_cc_GDP_PPP, re.form = NA, type = "response")
Atlas_Instr_predicted_cc_GDP_PPP <- Atlas_Instr_predicted_cc_GDP_PPP %>%
select(names(Atlas_Instr_obs_cc))

Atlas_Instr_predicted_imp_GDP_kEUR <- Country_list %>%
  left_join(GDP %>% select(country, GDP_cap_k)) %>%
  mutate(GDP_cap_k = as.numeric(GDP_cap_k),
        datatype = "Prediction_GDP_kEUR",
        Prop_frail_CI_lower = NA, Prop_frail_CI_upper = NA,
        Missingness = "Multiple imputation",
        Frailty_method = "SHARE Frailty Instrument")
Atlas_Instr_predicted_imp_GDP_kEUR$Prop_frail <- predict(
  model_i_predict, newdata = Atlas_Instr_predicted_imp_GDP_kEUR, re.form = NA, type =
"response")
Atlas_Instr_predicted_imp_GDP_kEUR <- Atlas_Instr_predicted_imp_GDP_kEUR %>%
select(names(Atlas_Instr_obs_cc))

Atlas_Instr_predicted_imp_GDP_PPP <- Country_list %>%
  left_join(GDP_PPP %>% select(country, GDP_cap_PPP_k)) %>%
  mutate(GDP_cap_PPP_k = as.numeric(GDP_cap_PPP_k),
        datatype = "Prediction_GDP_PPP",
        Prop_frail_CI_lower = NA, Prop_frail_CI_upper = NA,
        Missingness = "Multiple imputation",
        Frailty_method = "SHARE Frailty Instrument")
Atlas_Instr_predicted_imp_GDP_PPP$Prop_frail <- predict(
  model_i_predict_ppp, newdata = Atlas_Instr_predicted_imp_GDP_PPP, re.form = NA, type =
"response")
```

```
Atlas_Instr_predicted_imp_GDP_PPP <- Atlas_Instr_predicted_imp_GDP_PPP %>%
select(names(Atlas_Instr_obs_cc))

Atlas_Instrument <- rbind(Atlas_Instr_obs_cc,
                    Atlas_Instr_obs_imp,
                    Atlas_Instr_predicted_cc_GDP_kEUR,
                    Atlas_Instr_predicted_cc_GDP_PPP,
                    Atlas_Instr_predicted_imp_GDP_kEUR,
                    Atlas_Instr_predicted_imp_GDP_PPP)
write.csv(as.data.frame(Atlas_Instrument), "Atlas_Instrument_20230927.csv")




################################

#  7. Tables and figures for the manuscript
################################
# Figure 1. Flowchart / data on sample size in Frailty Instrument analyses
# exclusion of waves 5 & 7
table(Wall$Wave %in% c("W5", "W7"))
# descriptive, complete case / multiple imputation
length(Wall_c$mergeid)
length(Wall_i$mergeid)
# regression analysis, complete case / multiple imputation
length(Wall_c$mergeid[Wall_c$country != "Luxembourg"])
length(Wall_i$mergeid[Wall_i$country != "Luxembourg"])

#######
# Supplementary Table 1. Data missingness on frailty assessment components
# by agebands
levels(Wall2$Agebands) <- c(levels(Wall2$Agebands), "85 or higher")
Wall2$Agebands[Wall2$Agebands %in% c("85 to 89", "90 or higher")] <- "85 or higher"

Wall2 %>% group_by(Agebands) %>%
  summarize(Total = n(), Complete = sum(CompleteCase), Incomplete = n() - sum(CompleteCase),
        Total_percent = round(Total / length(Wall2$mergeid), 4),
        Complete_agedistr = round(Complete / length(Wall2$mergeid[Wall2$CompleteCase == T]),
4),
        Prop_incomplete = round(Incomplete  / Total, 4)) %>%
  ungroup() %>% as.data.frame() %>% print() %>% filter(!is.na(Agebands)) %>%
  select(Agebands, Complete, Incomplete) %>%
    column_to_rownames("Agebands") %>% chisq.test()

# by gender
Wall2 %>% group_by(gender) %>% summarize(
  Total = n(), Complete = sum(CompleteCase), Incomplete = n() - sum(CompleteCase),
  Total_percent = round(Total / length(Wall2$mergeid), 4),
  Prop_incomplete = round(Incomplete  / Total, 4)) %>% ungroup() %>% as.data.frame() %>% print()
%>%
  filter(!is.na(gender)) %>% select(gender, Complete, Incomplete) %>%
```

```
  column_to_rownames("gender") %>% chisq.test()
# by Fatigue
Wall2 %>% group_by(Fatigue) %>% summarize(Total = n(), Complete = sum(CompleteCase),
          Incomplete = n() - sum(CompleteCase), Total_percent = round(Total / length(Wall2$mergeid),
4),
          Prop_incomplete = round(Incomplete  / Total, 4)) %>% ungroup() %>% as.data.frame() %>%
print() %>%
  filter(!is.na(Fatigue)) %>% select(Fatigue, Complete, Incomplete) %>%
  column_to_rownames("Fatigue") %>% chisq.test()
# by Loss of appetite
Wall2 %>% group_by(Loss_of_appetite) %>%
  summarize(Total = n(),
          Complete = sum(CompleteCase),
          Incomplete = n() - sum(CompleteCase),
          Total_percent = round(Total / length(Wall2$mergeid), 4),
          Prop_incomplete = round(Incomplete  / Total, 4)) %>% ungroup() %>% as.data.frame() %>%
print() %>%
  filter(!is.na(Loss_of_appetite)) %>% select(Loss_of_appetite, Complete, Incomplete) %>%
  column_to_rownames("Loss_of_appetite") %>% chisq.test()
# by Grip srength
Wall2 %>% group_by(is.na(Gripstrength)) %>%
  summarize(Total = n(), Complete = sum(CompleteCase), Incomplete = n() - sum(CompleteCase),
          Total_percent = round(Total / length(Wall2$mergeid), 4),
          Prop_incomplete = round(Incomplete  / Total, 4)) %>% ungroup() %>% as.data.frame() %>%
print() %>%
  select(3,4) %>% chisq.test()
# by Slowness
Wall2 %>% group_by(Slowness) %>% summarize(Total = n(), Complete = sum(CompleteCase),
    Incomplete = n() - sum(CompleteCase), Total_percent = round(Total / length(Wall2$mergeid), 4),
    Prop_incomplete = round(Incomplete  / Total, 4)) %>% ungroup() %>% as.data.frame() %>% print()
%>%
  filter(!is.na(Slowness)) %>% select(Slowness, Complete, Incomplete) %>%
  column_to_rownames("Slowness") %>% chisq.test()
# by Low physical activity
Wall2 %>% group_by(Low_activity) %>% summarize(Total = n(), Complete = sum(CompleteCase),
                              Incomplete = n() - sum(CompleteCase), Total_percent = round(Total /
length(Wall2$mergeid), 4),
                              Prop_incomplete = round(Incomplete  / Total, 4)) %>% ungroup() %>%
as.data.frame() %>% print() %>%
  filter(!is.na(Low_activity)) %>% select(Low_activity, Complete, Incomplete) %>%
  column_to_rownames("Low_activity") %>% chisq.test()
#######

# Table 1. Study population by countries (number of surveys / number of subjects)

T1a <- imp_long %>%
  filter(.imp > 0) %>%
  group_by(country) %>%
  summarize(
    Full_surveys = n()/10,
    Full_subjects = length(unique(mergeid))) %>%
```

```
  ungroup() %>%
  as.data.frame()

T1b <- Wall_c %>%
  group_by(country) %>%
  summarize(
    Complete_surveys = n(),
    Complete_subjects = length(unique(mergeid))) %>%
  ungroup() %>%
  as.data.frame()
T1 <- T1a %>% left_join(T1b)
T1[length(T1$country)+1,] <- c("Total",
                        sum(T1$Full_surveys),
                        sum(T1$Full_subjects),
                        sum(T1$Complete_surveys),
                        sum(T1$Complete_subjects)) # data for the TOTAL row

### Table 2. Study population characteristics
#Gender
table(Wall2$gender)
table(Wall2$gender[Wall2$CompleteCase == T])
#Age
summary(Wall2$Age); IQR(Wall2$Age)
summary(Wall2$Age[Wall2$CompleteCase == T]); IQR(Wall2$Age[Wall2$CompleteCase == T])
#Education
Wall2 %>% select(mergeid, Wave) %>% left_join(EDU_table2) %>% select(Edu_y_i) %>%
summary(na.rm = T)
Wall2 %>% select(mergeid, Wave, CompleteCase) %>% left_join(EDU_table2) %>%
filter(CompleteCase == T) %>% select(Edu_y_i) %>% summary(na.rm = T)
#Marital
table(Wall_i$MaritalStatus) / length(Wall_i$MaritalStatus)
table(Wall_i$MaritalStatus[Wall_i$FI_missingdim == 0]) /
length(Wall_i$MaritalStatus[Wall_i$FI_missingdim == 0])
#Self-perceived health
table(Wall_i$Health) / length(Wall_i$Health)
table(Wall_i$Health[Wall_i$FI_missingdim == 0]) / length(Wall_i$Health[Wall_i$FI_missingdim ==
0])


### Figure 2. Frailty rates by age and sex: complete vs incomplete cases
Fig2data_Instrument <-
  Summary_i %>% select(Agebands, gender, N_total, N_frail) %>% group_by(Agebands, gender) %>%
  summarize(
    N_total = sum(N_total), N_frail = sum(N_frail), Prop_frail = N_frail / N_total,
    Prop_frail_CI_lower = exactci(N_frail, N_total, 0.95)[[1]][1],
    Prop_frail_CI_upper = exactci(N_frail, N_total, 0.95)[[1]][2],
    Analysis = "Full sample",
    Frailty_method = "Frailty Instrument") %>%
  ungroup %>% as.data.frame() %>% rbind(
    Summary_c %>% select(Agebands, gender, N_total, N_frail) %>% group_by(Agebands, gender)
%>%
```

```
    summarize(
      N_total = sum(N_total), N_frail = sum(N_frail), Prop_frail = N_frail / N_total,
      Prop_frail_CI_lower = exactci(N_frail, N_total, 0.95)[[1]][1],
      Prop_frail_CI_upper = exactci(N_frail, N_total, 0.95)[[1]][2],
      Analysis = "Complete cases", Frailty_method = "Frailty Instrument") %>% ungroup %>%
as.data.frame())

Fig2data_Index <- read.csv("Fig2data_index_20230927.csv", stringsAsFactors = F) %>%
rename(Analysis = Missingness)
Fig2data_Index$Analysis[Fig2data_Index$Analysis == "Multiple imputation"] <- "Full sample"
Fig2data_Index$Analysis[Fig2data_Index$Analysis == "Observed cases"] <- "Complete cases"
Fig2data_Index <- Fig2data_Index %>% select(-1)

Fig2data <- rbind(Fig2data_Index, Fig2data_Instrument)
rm(Fig2data_Index, Fig2data_Instrument)
Fig2 <- ggplot(data = Fig2data)  +
  geom_errorbar(aes(x = reorder(Analysis, desc(Analysis)), ymax = Prop_frail_CI_upper, ymin =
Prop_frail_CI_lower),
        size = 0.8, width = 0.6) +
  geom_col(aes(x = reorder(Analysis, desc(Analysis)), y = Prop_frail, fill = Analysis), colour = "black") +
   facet_grid(Frailty_method + gender ~ Agebands) + ylab("Proportion frail") + xlab("Ageband") +
  theme_minimal() +
  theme(axis.text.x=element_blank(),
     axis.ticks.x=element_blank()) + scale_x_discrete(position = "top") +
  scale_fill_grey(start = 0.7, end = 0.3) + scale_color_grey(start = 0.7, end = 0.3)

### Figure 3. Frailty rates by age, gender, and GDP per capita (in kEUR).
Fig3data_Instrument_cc <- Summary_c %>%
  select(country, Agebands, gender, GDP_cap_k, Prop_frail, Predict_prob, N_total) %>%
  mutate(Analysis = "Complete cases", Frailty_method = "Frailty Instrument")
Fig3data_Instrument_imp <- Summary_i %>%
  select(country, Agebands, gender, GDP_cap_k, Prop_frail, Predict_prob, N_total) %>%
  mutate(Analysis = "Full sample", Frailty_method = "Frailty Instrument")
Fig3data_Index <- read.csv("Fig3data_index_20230927.csv", stringsAsFactors = F) %>% select(-1)
Fig3data <- rbind(Fig3data_Instrument_cc, Fig3data_Instrument_imp, Fig3data_Index)

### Figure S1. Frailty rates by age, gender, and GDP per capita (PPP, in thousand EUR).
Fig4data_Instrument_cc <- Summary_c %>%
  select(country, Agebands, gender, GDP_cap_PPP_k, Prop_frail, Predict_prob_ppp, N_total) %>%
  mutate(Analysis = "Complete cases", Frailty_method = "Frailty Instrument")
Fig4data_Instrument_imp <- Summary_i %>%
  select(country, Agebands, gender, GDP_cap_PPP_k, Prop_frail, Predict_prob_ppp, N_total) %>%
  mutate(Analysis = "Full sample", Frailty_method = "Frailty Instrument")
Fig4data_Index <- read.csv("Fig4data_index_20230927.csv", stringsAsFactors = F) %>% select(-1)
Fig4data <- rbind(Fig4data_Instrument_cc, Fig4data_Instrument_imp, Fig4data_Index)

#Figure3A Females
Fig3A <- ggplot(Fig4data %>% filter(gender == "Female")) +
  geom_point(aes(x=GDP_cap_PPP_k, y=Prop_frail, size = N_total), shape = 21, color = "black", fill =
NA) +
  geom_line(aes(x=GDP_cap_PPP_k, y=Predict_prob_ppp), linetype = "solid", color = "red", size = 1) +
```

```
  facet_grid(Frailty_method + Analysis ~ Agebands) + theme_clean() +
  ylab("Proportion frail") + xlab("Annual GDP per capita (PPP) in 2000-2004, thousand EUR") +
  theme_minimal() + theme(axis.text.x = element_text(angle = 90)) +
  scale_size(name = "Number of surveys")

#Figure3B Males
Fig3B <- ggplot(Fig4data %>% filter(gender == "Male")) +
  geom_point(aes(x=GDP_cap_PPP_k, y=Prop_frail, size = N_total), shape = 21, color = "black", fill =
NA) +
  geom_line(aes(x=GDP_cap_PPP_k, y=Predict_prob_ppp), linetype = "solid", color = "red", size = 1) +
  facet_grid(Frailty_method + Analysis ~ Agebands) + theme_clean() +
  ylab("Proportion frail") + xlab("Annual GDP per capita (PPP) in 2000-2004, thousand EUR") +
  theme_minimal() + theme(axis.text.x = element_text(angle = 90)) +
  scale_size(name = "Number of surveys")

#Figure S1A Females
FigS1A <- ggplot(Fig3data %>% filter(gender == "Female")) +
  geom_point(aes(x=GDP_cap_k, y=Prop_frail, size = N_total), shape = 21, color = "black", fill = NA) +
  geom_line(aes(x=GDP_cap_k, y=Predict_prob), linetype = "solid", color = "red", size = 1) +
  facet_grid(Frailty_method + Analysis ~ Agebands) + theme_clean() +
  ylab("Proportion frail") + xlab("Annual GDP per capita in 2000-2004, in thousand EUR") +
  theme_minimal() + theme(axis.text.x = element_text(angle = 90)) +
  scale_size(name = "Number of surveys")

#Figure S1B Males
FigS1B <- ggplot(Fig3data %>% filter(gender == "Male")) +
  geom_point(aes(x=GDP_cap_k, y=Prop_frail, size = N_total), shape = 21, color = "black", fill = NA) +
  geom_line(aes(x=GDP_cap_k, y=Predict_prob), linetype = "solid", color = "red", size = 1) +
  facet_grid(Frailty_method + Analysis ~ Agebands) + theme_clean() +
  ylab("Proportion frail") + xlab("Annual GDP per capita in 2000-2004, in thousand EUR") +
  theme_minimal() + theme(axis.text.x = element_text(angle = 90)) +
  scale_size(name = "Number of surveys")


################################
```
